# Epidemiology of herpes simplex virus type 2 in Europe: systematic review, meta-analyses, and meta-regressions

**DOI:** 10.1016/j.lanepe.2022.100558

**Published:** 2022-12-12

**Authors:** Asalah Alareeki, Aisha M.M. Osman, Mohannad N. Khandakji, Katharine J. Looker, Manale Harfouche, Laith J. Abu-Raddad

**Affiliations:** a Infectious Disease Epidemiology Group, Weill Cornell Medicine-Qatar, Cornell University, Doha, Qatar; b World Health Organization Collaborating Centre for Disease Epidemiology Analytics on HIV/AIDS, Sexually Transmitted Infections, and Viral Hepatitis, Weill Cornell Medicine–Qatar, Cornell University, Qatar Foundation–Education City, Doha, Qatar; c Population Health Sciences, Bristol Medical School, University of Bristol, Bristol, United Kingdom; d Department of Population Health Sciences, Weill Cornell Medicine, Cornell University, New York, USA; e Department of Public Health, College of Health Sciences, Member of QU Health, Qatar University, Doha, Qatar

**Keywords:** Epidemiology, Herpes simplex virus, HSV-2, Genital herpes, Genital ulcer, Sexually transmitted infection, Prevalence, Europe

## Abstract

**Background:**

Herpes simplex virus type 2 (HSV-2) infection is a globally prevalent, life-long, sexually transmitted infection. This study characterized HSV-2 seroprevalence in Europe for various at-risk populations and proportions of HSV-2 detection in genital ulcer disease (GUD) and in genital herpes. Data on neonatal herpes and HSV-2's contribution to HIV transmission were also reviewed.

**Methods:**

Cochrane and PRISMA guidelines were followed to systematically review, synthesize, and report HSV-2 related findings. The search was conducted in PubMed and Embase databases up to February 20, 2022. Any publication reporting data on the outcome measures was included. Meta-analyses and meta-regressions were conducted.

**Findings:**

211 relevant reports were identified, including 12 overall incidence measures, 294 overall (813 stratified by factors such as age and sex) seroprevalence measures, 13 overall (15 stratified by sex) proportions of HSV-2 detection in clinically diagnosed GUD, and 70 overall (183 stratified by factors such as age and sex) proportions of HSV-2 detection in laboratory-confirmed genital herpes. Pooled mean seroprevalence was 12.4% (95% CI: 11.5–13.3%) among general populations, 27.8% (95% CI: 17.5–39.4%) among men who have sex with men, 46.0% (95% CI: 40.1–51.8%) among people living with HIV and people in HIV discordant couples, and 63.2% (95% CI: 55.5–70.6%) among female sex workers. Most measures showed heterogeneity in HSV-2 seroprevalence. The pooled mean seroprevalence among general populations increased with age and was 0.65-fold (95% CI: 0.58–0.74) lower in men than women. Seroprevalence decreased by 1% per calendar year. Pooled mean proportions of HSV-2 detection in GUD and in genital herpes were 22.0% (95% CI: 15.3–29.6%) and 66.0% (95% CI: 62.9–69.1%), respectively. HSV-2 detection in genital herpes cases was 1.21-fold (95% CI: 1.10–1.32) higher in men compared to women and decreased by 1% per calendar year. Incidence of neonatal herpes indicated an increasing trend.

**Interpretation:**

Although seroprevalence is declining, a significant proportion of Europe's population is infected with HSV-2. HSV-2 accounts for approximately one-fifth of GUD cases and two-thirds of genital herpes cases. Findings support the need to invest in HSV-2 vaccine development, and sexual and reproductive health services.

**Funding:**

10.13039/100008982Qatar National Research Fund [NPRP 9-040-3-008] and pilot funding from the Biomedical Research Program at 10.13039/100019460Weill Cornell Medicine in Qatar supported this study.

**Research in context**

**Evidence before this study**

Herpes simplex virus type 2 (HSV-2) infection is a globally prevalent sexually transmitted infection that causes mild to severe disease. Despite over 30 years of active research, the global epidemiology of this infection remains inadequately characterized. A study by Yousuf et al^1^ investigated epidemiology of HSV-1 in Europe, a closely related infection to HSV-2. The study found that although HSV-1 seroprevalence is declining, it is becoming the leading cause of genital herpes in Europe.^1^ Global estimates for HSV-2 and HSV-1 infections have been published by James et al^2^ using mathematical modelling. These indicated that, in Europe in 2016, 10.7% of females and 5.3% of males 15–49 years of age were infected with HSV-2, and 60.6% of females and 40.1% of males 0–49 years of age were infected with HSV-1.^2^ Prior to starting this study, a PubMed search was conducted using a broad search strategy (using the keywords “Herpes Simplex”[MeSH] AND “Review”[Publication Type]) with no language or time limitations. No systematic review and meta-analytics investigating epidemiology of HSV-2 infection in Europe were identified.

**Added value of this study**

By implementing rigorous methodologies, this study provided an analytical assessment of the epidemiology of HSV-2 infection in Europe. After searching international electronic databases, a large body of data was identified, facilitating a range of analyses. Approximately 12% of the adult population of Europe is chronically infected with HSV-2, but seroprevalence is declining by 1% per calendar year. Sexual risk behaviour, age, sex, and subregion within Europe explained a large proportion of the variation in seroprevalence in this continent. HSV-2 was the aetiological cause of 22% of clinically diagnosed genital ulcer disease cases and 66% of laboratory-confirmed genital herpes. The contribution of HSV-2 infection (as opposed to HSV-1 infection) to genital herpes cases was declining by 1% per calendar year.

**Implications of all the available evidence**

Though slowly declining, HSV-2 disease burden remains sizable in Europe, against a background of non-specific, and no targeted, public health interventions to prevent and control transmission of this infection. In the context of serious HSV-2 disease sequelae affecting reproductive, sexual, and psychosocial health, these findings highlight the public health value of accelerating HSV-2 vaccine development as a fundamental solution to tackle this infection in Europe and elsewhere. HSV-2 research and surveillance should be expanded by conducting national population-based seroprevalence surveys and monitoring aetiology of genital ulcer disease and genital herpes.

## Introduction

Herpes simplex virus type 2 (HSV-2) infection is a life-long, incurable, sexually (or vertically) transmitted infection (STI) that is prevalent worldwide.^2^ Although asymptomatic in most people, HSV-2 infection can cause a range of symptoms and adverse health outcomes such as recurrent genital lesions and serious neonatal infections.^3^, ^4^, ^5^ HSV-2 infection is characterized by frequent subclinical shedding and reactivations^6^ that are linked to increased sexual transmission and acquisition of HIV,^7^^,^^8^ perhaps causing epidemiological synergy between these two infections.^7^^,^^9^^,^^10^

Inadequate understanding of HSV-2 epidemiology and its consequences for sexual and reproductive health and the HIV epidemic, call for further research and for preventive and control measures.^3^^,^^4^^,^^11^ The World Health Organization (WHO) issued Global Health Sector Strategies on STIs^12^^,^^13^ to eliminate STIs as a main public health concern by 2030, through integration of preventive and control measures. WHO and global partners are prioritizing efforts to develop an HSV vaccine.^2^^,^^14^^,^^15^

Global estimates published by James et al^2^ using mathematical modelling indicated that, in Europe in 2016, 10.7% of females and 5.3% of males 15–49 years of age were infected with HSV-2, and 60.6% of females and 40.1% of males 0–49 years of age were infected with HSV-1. However, despite over three decades of active HSV-2 epidemiology research, no specific systematic review and meta-analytics investigating epidemiology of HSV-2 infection in Europe were published.

Accordingly, we conducted a systematic review and meta-analytics to characterize HSV-2 epidemiology in Europe. Pooled mean HSV-2 antibody prevalence (seroprevalence) was estimated for various at-risk populations, including general populations (such as antenatal clinic attendees, blood donors, and pregnant women), intermediate-risk populations (such as prisoners, people who inject drugs, and truck drivers), higher-risk populations (such as female sex workers (FSWs), men who have sex with men (MSM), male sex workers, and transgender people), STI clinic attendees and symptomatic populations, people living with HIV and people in HIV discordant couples, infertility clinic attendees and women with ectopic pregnancies, and other populations (such as cervical cancer patients and their spouses) (Box S1). Pooled mean proportions of HSV-2 virus detection in clinically diagnosed genital ulcer disease (GUD) cases and in laboratory-confirmed genital herpes cases were also estimated. Data on neonatal herpes and HSV-2's contribution to HIV transmission were also reviewed.

Both overall measures and stratified measures were extracted from relevant studies included in this review. Since our aim was to understand the natural heterogeneity that exists in HSV-2 epidemiology, such as the variation in seroprevalence by age, measures were extracted and stratified by key epidemiological factors known to affect the natural epidemiology of this infection.^16^, ^17^, ^18^, ^19^ Meta-regression analyses were conducted on these stratified measures to estimate effects of these epidemiological factors on both HSV-2 seroprevalence and proportion of HSV-2 detection in genital herpes. The meta-regression analyses were also conducted to investigate temporal trends and sources of between-study heterogeneity. This analytical approach combining meta-regressions with meta-analyses allows the generation of fundamental inferences about the epidemiology of this infection based on understanding the sources of variations that exist in available measures.

## Methods

The methodology used in this study was based on that developed in a series of published systematic reviews and meta-analyses characterizing the epidemiology of HSV-1 and HSV-2 infections in other regions and countries.^1^^,^^16^, ^17^, ^18^, ^19^, ^20^, ^21^, ^22^, ^23^, ^24^ Therefore, no study protocol was registered in PROSPERO for this specific study. Study methods are described in Box 1 and summarized below.

Box 1 **Description of the methodology of this study.**

**Table:** 

Methodology	Description
Data source and search strategy	- Search was first conducted on January 12, 2021, and then updated on February 20, 2022, in PubMed and Embase. - Search strategies included exploded MeSH/Emtree terms and broad terms with no language or time restrictions. - The definition of Europe included 53 countries classified by European subregion/country: ○ Eastern Europe: Belarus, Bulgaria, Czech Republic, Hungary, Poland, Republic of Moldova, Romania, Russian Federation, Slovakia, and Ukraine. ○ Northern Europe: Denmark, Estonia, Finland, Iceland, Ireland, Latvia, Lithuania, Norway, Sweden, and United Kingdom of Great Britain and Northern Ireland. ○ Southern Europe: Albania, Andorra, Bosnia and Herzegovina, Croatia, Greece, Italy, Malta, Montenegro, Portugal, the former Yugoslav Republic of Macedonia, San Marino, Serbia, Slovenia, and Spain. ○ Western Europe: Austria, Belgium, France, Germany, Luxembourg, Monaco, Netherlands, and Switzerland. ○ Intersection of Europe and Asia: Armenia, Azerbaijan, Cyprus, Georgia, Kazakhstan, Kyrgyzstan, Tajikistan, Turkmenistan, and Uzbekistan. ○ Israel. ○ Turkey.
Study selection and inclusion and exclusion criteria	- Search results were imported into the reference manager Endnote (Thomson Reuters, USA). - Screening was performed in four stages: 1. Duplicate publications were identified and excluded. 2. Titles and abstracts were screened for relevant and potentially relevant publications. 3. Full texts of relevant and potentially relevant publications were retrieved and screened for relevance. 4. Bibliographies of relevant publications and reviews were checked for additional potentially relevant publications. - Inclusion criteria were any publication, including a study with a minimum sample size of 10, reporting primary data on any of the following outcome measures: 1. HSV-2 antibody incidence as detected by a type-specific diagnostic assay. 2. HSV-2 antibody seroprevalence as detected by a type-specific diagnostic assay. 3. Proportion of HSV-2 detection in clinically diagnosed GUD as detected by standard viral detection and subtyping methods. 4. Proportion of HSV-2 detection in laboratory-confirmed genital herpes (as opposed to HSV-1) as detected by standard viral detection and subtyping methods. - Exclusion criteria were: ○ Case reports, case series, reviews, editorials, commentaries, and qualitative studies. ○ Measures reporting seroprevalence in infants aged <6 months as their antibodies can be maternal in origin. - In this study, the term “publication” refers to a document reporting one or several outcome measures. “Study” or “measure” refers to a specific outcome measure and its details.
Data extraction and data synthesis	- Extracted variables included: author(s), publication title, year of publication, year(s) of data collection, subregion, country of origin, country of survey, city, study site, study design, study sampling procedure, study population and its characteristics (e.g., sex and age), sample size, response rate, HSV-2 outcome measures, and diagnostic assay (Box S2). - Overall outcome measures and their stratified measures were extracted, provided sample size in each stratum is ≥10. - For studies including overall sample size, but no individual strata sample sizes, the sample size of each stratum was assumed equal to overall sample size divided by the number of strata in the study. - Stratification hierarchy for incidence and seroprevalence measures in descending order of preference were: 1. Population type as defined in Box S1. 2. Sex. 3. Age group classified as (groups optimized to best fit reported data): ○ <20 years old. ○ 20–29 years old. ○ 30–39 years old. ○ 40–49 years old. ○ 50–59 years old ○ ≥60 years old. ○ Mixed ages. - Stratification hierarchy for GUD and genital herpes included genital herpes episode status and study site: 1. Genital herpes episode status classified as: ○ First episode genital herpes. ○ Recurrent genital herpes. 2. Study site stratification classified as: ○ Hospital. ○ STI clinic. - Measures reporting any HSV-2 outcome among children <15 years old were only reported but not included in the analyses.
Quality assessments	The Cochrane-informed approach for risk of bias assessment included: - Study's precision classification into low *versus* high was based on the sample size (<200 *versus* ≥200). - Study's appraisal into low *versus* high risk of bias was determined using two quality domains: ○ Sampling method: probability-based *versus* non-probability-based. ○ Response rate: ≥80% *versus* <80% or unclear.
Meta-analyses	- Meta-analyses were conducted using DerSimonian-Laird random-effects models with inverse variance weighting. The variance of each outcome measure was stabilized using the Freeman-Tukey arcsine square-root transformation. - Pooled mean HSV-2 seroprevalence was estimated for each population type by sex, and for general populations by European country, European subregion, age group, year of data collection range, and year of publication range. - Pooled proportions of HSV-2 detection in GUD and in genital herpes were estimated. - Heterogeneity assessment was based on three complementary metrics: ○ Cochran's Q statistic to assess existence of heterogeneity in effect size (p-value < 0.1 indicated heterogeneity). ○ I^2^ heterogeneity measure to assess the percentage of between-study variation in effect size that is due to actual differences in effect size rather than sampling variation. ○ Prediction interval to describe the distribution of true outcome measures around the pooled mean.
Meta-regressions	- Univariable and multivariable random-effects meta-regression analyses using log-transformed proportions were carried out to identify predictors of HSV-2 seroprevalence and proportion of HSV-2 detection in genital herpes. - Factors in the univariable analyses with a p-value <0.1 were included in the multivariable analysis. - Factors in the multivariable analyses with a p-value ≤0.05 were deemed to be significant predictors. - Variables included in the univariable meta-regression models for HSV-2 seroprevalence were: ○ Population type. ○ Age group. ○ Sex. ○ European subregion/country. ○ Country's income. ○ Assay type (western blot, ELISA, and monoclonal antibody). ○ Sample size. ○ Sampling method. ○ Response rate. ○ Year of data collection. ○ Year of publication. ○ Year of data collection category (<1995, 1995–2005, >2005). ○ Year of publication category (<2000, 2000–2010, >2010). - Variables included in the univariable meta-regression models for proportion of HSV-2 detection in genital herpes were: ○ Age group. ○ Sex. ○ Genital herpes episode status. ○ European subregion/country. ○ Sample size. ○ Year of data collection category (<1995, 1995–2005, >2005). ○ Year of publication category (<2000, 2000–2010, >2010). - The year of data collection had missing variables that were imputed by adjusting the year of publication using the median difference with the year of data collection in studies with reported year of data collection.

Abbreviations: ELISA = Enzyme-linked immunosorbent type-specific assay, GUD = Genital ulcer disease, HSV-1 = Herpes simplex virus type 1, HSV-2 = Herpes simplex virus type 2, STI = Sexually transmitted infection.

### Data sources and search strategy

This systematic review was informed by the Cochrane Collaboration Handbook^25^ and its findings are reported as per the Preferred Reporting Items for Systematic Reviews and Meta-analyses (PRISMA) guidelines (Table S1).^26^^,^^27^ The search was first conducted in PubMed and Embase databases on January 12, 2021, with no language or time limitations, and then updated on February 20, 2022. The definition of Europe included 53 countries classified into subregions as informed by the WHO and United Nations Geoscheme definitions for Europe (Box 1).^28^^,^^29^ Search strategies are indicated in Box 1 and explicitly included in Table S2.

### Study selection and inclusion and exclusion criteria

Screening steps and inclusion and exclusion criteria are described in Box 1. Using the reference manager Endnote (Thomson Reuters, USA), citations were imported from PubMed and Embase databases and duplicate citations were excluded. Title and abstract screening was performed to identify relevant and potentially relevant publications. Full texts of relevant and potentially relevant publications were retrieved. Bibliography screening of relevant publications and reviews was conducted to identify additional potentially relevant publications.

Inclusion criteria involved any publication with a minimum sample size of 10, reporting primary data on HSV-2 antibody incidence and/or seroprevalence, proportion of HSV-2 detection in clinically diagnosed GUD, and proportion of HSV-2 detection in laboratory-confirmed genital herpes. Exclusion criteria included case reports, case series, reviews, editorials, commentaries, and qualitative studies. Measures reporting seroprevalence in infants aged <6 months were excluded as their antibodies can be maternal in origin.

### Data extraction and data synthesis

Screening of retrieved citations and articles was conducted by A.A., A.M.M.O., M.N.K., and M.H. All citations and articles retrieved were first independently screened by M.N.K., then, following the updated search, citations and articles retrieved were divided among A.A., A.M.M.O., and M.H. and once more independently screened. Data extraction was performed by A.M.M.O., A.A., and M.N.K. Double extraction was performed by M.H. Discrepancies were discussed in consultation with L.J.A.-R. to reach consensus. The extracted variables are indicated in Box 1 and listed in Box S2.

Overall outcome measures and their stratified measures were extracted, provided sample size in each stratum is ≥10. In occasions in which the subsample sizes for specific strata were not available, this subsample size was set at the total sample size of the study divided by the number of strata. Data from included studies were collected between 1968 and 2019 and were published between 1977 and 2021. The year of data collection was missing for a small proportion of studies, but it was imputed by adjusting the year of publication using the median difference with the year of data collection in studies with reported year of data collection.

Stratification hierarchy for incidence and seroprevalence measures in descending order of priority was population type, sex, and age group (<20 years old, 20–29 years old, 30–39 years old, 40–49 years old, 50–59 years old, ≥60 years old, and mixed ages). Stratification hierarchy for GUD and genital herpes included genital herpes episode status (first episode *versus* recurrent episodes) and study site (hospital *versus* STI clinic). All relevant data are presented in the manuscript and its Supplementary Material file. Detailed database of extracted variables can be obtained by contacting the authors.

### Quality assessments

Due to known limitations of HSV-2 assays,^30^, ^31^, ^32^ a quality assessment of the assay in each relevant study was conducted with the assistance of Professor Rhoda Ashley–Morrow of the University of Washington – a leading expert in HSV-2 serological assays who has investigated and evaluated the validity and reliability of different assays for three decades. Professor Ashley–Morrow contributed as a compensated consultant. Details of each assay in each study were shared with Professor Ashley–Morrow. Validity and reliability of each assay was based on her expert judgment. Only studies with valid and reliable assays were included in the systematic review.

Each study was then assessed for precision and risk of bias (ROB) as informed by the Cochrane approach.^25^ Precision of a study was classified as low *versus* high based on the sample size (<200 *versus* ≥200). ROB of a study was classified as low *versus* high based on the sampling method (probability-based *versus* non-probability-based) and response rate (≥80% *versus* <80% or unclear) (Box 1). Information on precision and ROB were utilized to provide summary statistics of the precision and quality of studies. These variables were also included in the meta-regressions to investigate their effect on observed seroprevalence.

### Meta-analyses and meta-regressions

All meta-analyses were conducted in R version 4.41.3^33^ using the “meta” package,^34^ as described in Box 1. To account for both sampling variation and heterogeneity in effect size, meta-analyses were conducted using the DerSimonian-Laird random-effects model,^35^ with the Freeman-Tukey double arcsine transformation to stabilize the variance,^36^ after factoring applicability of this transformation.^37^

Heterogeneity was assessed based on Cochran's Q statistic, I^2^ heterogeneity measure, and prediction interval. Extracted measures were described using summary statistics, including medians, ranges, and pooled mean estimates and their 95% confidence intervals (CI) for HSV-2 seroprevalence and for proportions of HSV-2 detection in GUD and in genital herpes. Forest plots of all meta-analyses were generated by population type classification and by subregion for the general populations.

Univariable and multivariable regressions of log-transformed seroprevalence measures were conducted in Stata/SE version 16^38^ using the “metareg” package^39^ (Box 1). All stratified seroprevalence measures were utilized in all population types. Variables included in the univariable meta-regressions for HSV-2 seroprevalence were population type, age group, sex, European subregion/country, country's income, assay type (Western blot, ELISA, and monoclonal antibody), sample size, sampling method, response rate, year of data collection, year of publication, year of data collection category (<1995, 1995–2005, >2005), and year of publication category (<2000, 2000–2010, >2010). Variables included in the univariable meta-regressions for proportion of HSV-2 detection in genital herpes were age group, sex, genital herpes episode status, European subregion/country, sample size, year of data collection category (<1995, 1995–2005, >2005), and year of publication category (<2000, 2000–2010, >2010). Variables in the univariable analyses with a p-value <0.1 were included in the multivariable meta-regression models.

Four multivariable meta-regression models were implemented for each of HSV-2 seroprevalence and proportion of HSV-2 detection in genital herpes. The first and second models included the year of data collection as categorical and continuous linear variables, respectively. The third and fourth models included the year of publication as categorical and continuous linear variables, respectively. Variables in the multivariable analyses with a p-value ≤0.05 were considered significant predictors. These four models were implemented to account for the collinearity between year of data collection and year of publication, and to investigate whether the trends may have been appropriately described using an average linear trend. Although the year of data collection is more relevant than the year of publication, we added analyses using the year of publication because it has no missing entries.

Subgroup analyses were also conducted for HSV-2 seroprevalence including only studies among general populations, as well as for proportion of HSV-2 detection in genital herpes, including only studies with reported genital herpes episode status.

### Neonatal herpes and role of HSV-2 in HIV transmission

A broad search of the literature was conducted to identify and extract epidemiological data on neonatal herpes in Europe (Table S3). PubMed and Embase were searched using the keywords “Neonatal herpes[MeSH] OR Neonatal herpes[Text])” and “(exp neonatal herpes) OR (neonatal herpes.mp.)”, respectively, with no language or time limitations. This search was conducted on August 16, 2022. Moreover, based on search terms used in a recent systematic review^8^ and as informed by two decades of investigating the overlapping epidemiology of HSV-2 and HIV,^7^, ^8^, ^9^, ^10^^,^^20^^,^^40^, ^41^, ^42^ a narrative review was conducted of estimates for the contribution of HSV-2 to HIV transmission in Europe.

### Role of the funding source

The funder of the study had no role in study design, data collection, data analysis, data interpretation, or writing of the article. The corresponding author had full access to all the data in the study and had the final responsibility for the decision to submit for publication.

## Results

### Search results and scope of evidence

**Fig. 1:**
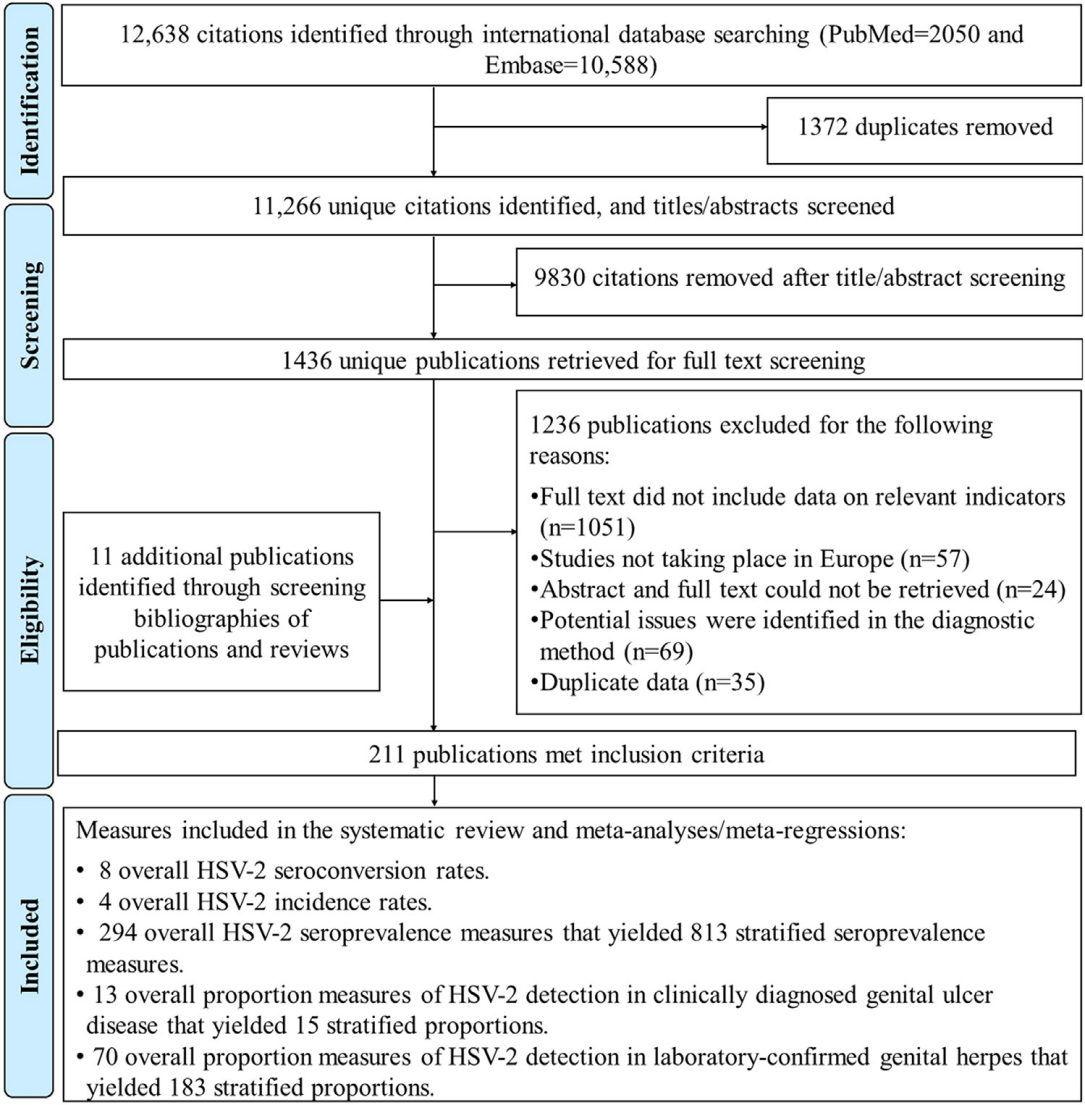
Flow chart of article selection for this systematic review of HSV-2 epidemiology in Europe, per PRISMA guidelines.^27^

Fig. 1 describes the study selection process following PRISMA guidelines. Overall, 12,638 citations were identified (PubMed = 2050 and Embase = 10,588). After de-duplication and title and abstract screening, 1436 unique citations were identified as relevant or potentially relevant for further screening. Full text screening of these citations identified 200 relevant publications. Eleven additional relevant publications were identified through bibliography screening, including one thesis.

In total, data were extracted from 211 publications that met the inclusion criteria. Extracted outcome measures included: 8 overall HSV-2 seroconversion rates, 4 overall HSV-2 incidence rates, 294 overall and 813 stratified HSV-2 seroprevalence measures, 13 overall and 15 stratified proportion measures of HSV-2 detection in GUD, and 70 overall and 183 stratified proportion measures of HSV-2 detection in genital herpes.

### HSV-2 incidence overview

Table S4 summarizes extracted seroconversion rates (n (number of measures) = 8) and incidence rates (n = 4). These measures were extracted from cohort studies with follow-up durations ranging between 42 days and 6 years. The studies were conducted in pregnant women, healthy adolescents, soldiers, STI clinic attendees, MSM, and MSM living with HIV. Across all populations, seroconversion rate ranged between 0.3 and 17.0% and incidence rate ranged between 0.1 and 1.3 per 100 person-years.

### HSV-2 seroprevalence overview

Tables S5 and S6 list overall extracted seroprevalence measures (n = 294). The earliest study was published in 1984, but the majority of studies (n = 162, 55.1%) were published between 2000 and 2010. Most studies (n = 251, 85.4%) used convenience sampling.

Descriptive statistics of all stratified seroprevalence measures by population type classification are presented in Table 1. HSV-2 seroprevalence ranged between 0.0 and 95.0% with a median of 11.3% among general populations (n = 626), between 2.0 and 32.8% with a median of 11.9% among intermediate-risk populations (n = 12), between 33.3 and 89.3% with a median of 59.6% among FSWs (n = 16), between 5.9 and 56.0% with a median of 34.5% among MSM (n = 12), between 2.0 and 84.9% with a median of 25.0% among STI clinic attendees and symptomatic populations (n = 74), between 9.0 and 94.0% with a median of 43.9% among people living with HIV and people in HIV discordant couples (n = 35), and between 2.0 and 24.0% with a median of 12.0% among infertility clinic attendees and women with ectopic pregnancies (n = 7). Descriptive statistics of stratified seroprevalence measures among only general populations are presented in Table 2.

**Table 1:** Pooled mean estimates for HSV-2 seroprevalence among different at-risk populations in Europe by sex.

Population type	Outcome measures	Sample size	HSV-2 seroprevalence (%)		Pooled mean HSV-2 seroprevalence	Heterogeneity measures		
	Total n	Total N	Range	Median	Mean (%) (95% CI)	Q^a^ (p-value)	I^2^^b^ (%) (95% CI)	Prediction interval^c^ (%)
General populations	626	154,957	0.0–95.0	11.3	12.4 (11.5–13.3)	13127.0 (p < 0.001)	95.2 (95.0–95.5)	0.0–38.8
Women	359	88,907	0.0–85.7	12.5	14.0 (12.8–15.3)	7690.7 (p < 0.001)	95.3 (95.0–95.6)	0.2–41.8
Men	186	38,478	0.0–40.8	8.5	8.2 (7.2–9.2)	2272.7 (p < 0.001)	91.9 (91.0–92.6)	0.1–24.9
Mixed	81	27,572	0.0–95.0	14.7	15.8 (12.8–19.2)	1964.4 (p < 0.001)	95.9 (95.4–96.4)	0.0–50.4
Intermediate-risk populations^d^	12	3119	2.0–32.8	11.9	12.3 (7.3–18.4)	241.4 (p < 0.001)	95.4 (93.5–96.8)	0.0–40.7
Women^e^	1	67	-	-	9.0 (3.4–18.5)	-	-	-
Men	6	1558	8.1–28.1	17.1	17.0 (11.5–23.3)	41.8 (p < 0.001)	88.0 (76.4–93.9)	2.1–41.4
Mixed	5	1494	2.0–32.9	5.1	8.2 (1.6–19.1)	93.7 (p < 0.001)	95.7 (92.5–97.6)	0.0–63.5
Higher-risk populations	28	9058	5.9–89.3	50.7	47.2 (37.6–57.0)	2276.4 (p < 0.001)	98.8 (98.6–99.0)	4.4–93.0
FSWs	16	2047	33.3–89.3	59.6	63.2 (55.5–70.6)	149.4 (p < 0.001)	90.0 (85.3–93.1)	30.7–90.3
MSM	12	7011	5.9–56.0	34.5	27.8 (17.5–39.4)	1177.8 (p < 0.001)	99.1 (98.8–99.2)	0.2–75.8
STI clinic attendees and symptomatic populations^f^	74	19,487	2.0–84.9	25.0	26.9 (23.1–30.8)	1725.8 (p < 0.001)	95.8 (95.2–96.3)	2.8–62.8
Women	29	6136	4.8–60.0	23.0	25.5 (20.8–30.5)	422.5 (p < 0.001)	93.4 (91.5–94.8)	5.0–54.5
Men	21	6144	2.0–35.0	18.5	17.4 (13.6–21.6)	340.4 (p < 0.001)	94.1 (92.2–95.6)	3.2–39.3
Mixed	24	7207	8.0–84.9	31.0	38.3 (29.9–47.0)	667.8 (p < 0.001)	96.6 (95.7–97.2)	4.2–81.2
People living with HIV and people in HIV discordant couples	35	3660	9.0–94.0	43.9	46.0 (40.1–51.8)	386.3 (p < 0.001)	91.2 (88.8–93.1)	15.0–78.8
Women	12	784	17.4–94.0	56.5	55.3 (41.4–68.8)	138.3 (p < 0.001)	92.0 (88.0–94.7)	7.8–97.1
Men	9	1370	31.0–62.0	50.0	46.4 (39.5–53.3)	64.9 (p < 0.001)	87.7 (78.7–92.9)	24.1–69.5
Mixed	14	1506	9.0–59.6	39.6	38.5 (31.7–45.5)	126.0 (p < 0.001)	89.7 (84.5–93.1)	14.3–66.1
Infertility clinic attendees and women with ectopic pregnancies	7	766	2.0–24.0	12.0	11.7 (5.8–19.2)	49.8 (p < 0.001)	87.9 (77.5–93.5)	0.0–43.0
Women	7	766	2.0–24.0	12.0	11.7 (5.8–19.2)	49.8 (p < 0.001)	87.9 (77.5–93.5)	0.0–43.0
Other populations	31	27,929	6.7–44.4	17.5	21.2 (17.4–25.2)	508.0 (p < 0.001)	94.1 (92.6–95.3)	4.4–45.7
Women	13	2052	11.4–42.1	16.7	20.1 (15.4–25.3)	93.3 (p < 0.001)	87.1 (79.8–91.8)	5.2–41.2
Men	4	535	6.7–44.4	24.6	23.1 (7.8–43.4)	78.8 (p < 0.001)	96.2 (92.9–98.0)	0.0–100
Mixed	14	25,342	9.3–43.9	21.8	21.8 (16.1–28.0)	296.5 (p < 0.001)	95.6 (94.0–96.8)	3.4–49.5

Abbreviations: CI = Confidence interval, FSWs = Female sex workers, HIV = Human immunodeficiency virus, HSV-2 = Herpes simplex virus type 2, MSM = Men who have sex with men, STI = Sexually transmitted infection.

a Q: The Cochran's Q statistic is a measure assessing the existence of heterogeneity in seroprevalence.

b I^2^: A measure that assesses the magnitude of between-study variation that is due to actual differences in seroprevalence across studies, rather than sampling variation.

c Prediction interval: A measure that estimates the distribution (95% interval) of true seroprevalence around the estimated mean.

d Intermediate-risk populations include populations who presumably have frequent sexual contacts with populations engaging in high sexual risk behaviour and have therefore a higher risk of exposure to HSV-2 than the general population. These comprise prisoners, people who inject drugs, and truck drivers, among others.

e No meta-analysis was conducted due to the small number of studies (n < 3).

f Symptomatic populations include patients with STI clinical manifestations.

**Table 2:** Pooled mean estimates for HSV-2 seroprevalence among general populations in Europe.

Population classification	Outcome measures	Sample size	HSV-2 seroprevalence (%)		Pooled mean HSV-2 seroprevalence	Heterogeneity measures		
	Total n	Total N	Range	Median	Mean (%) (95% CI)	Q^a^ (p-value)	I^2^^b^ (%) (95% CI)	Prediction interval^c^ (%)
European countries								
Belgium	18	3477	7.0–27.7	12.5	13.3 (10.8–15.9)	74.7 (p < 0.001)	77.2 (64.4–85.5)	4.7–25.2
Bulgaria	12	2508	2.0–40.0	20.0	19.1 (12.7–26.4)	182.1 (p < 0.001)	94.0 (91.2–95.9)	0.9–51.3
Croatia	17	2651	0.0–32.0	8.0	9.7 (6.1–14.0)	153.2 (p < 0.001)	89.6 (84.9–92.8)	0.0–32.2
Czech Republic	12	2570	2.0–8.5	4.0	4.8 (3.5–6.4)	30.9 (p = 0.001)	64.4 (33.9–80.8)	1.2–10.5
Estonia	20	3049	1.8–34.0	17.9	16.5 (12.1–21.4)	222.2 (p < 0.001)	91.4 (88.2–93.8)	1.4–42.4
Finland	32	8089	0.5–51.7	16.5	16.0 (12.3–20.0)	556.0 (p < 0.001)	94.4 (93.0–95.5)	1.0–42.6
France	12	9389	12.5–28.3	17.1	17.3 (15.4–19.2)	35.0 (p < 0.001)	68.6 (42.8–82.7)	11.2–24.4
Germany	65	23,609	0.0–25.0	11.5	11.5 (10.1–13.0)	673.1 (p < 0.001)	90.5 (88.6–92.1)	2.7–25.2
Hungary	17	3012	0.0–12.2	2.7	3.6 (2.0–5.5)	103.5 (p < 0.001)	84.5 (76.6–89.8)	0.0–14.2
Israel	29	4126	0.0–33.1	7.7	9.5 (7.1–12.2)	218.2 (p < 0.001)	87.2 (82.7–90.5)	0.6–26.1
Italy	60	4706	0.0–95.0	13.0	17.3 (12.1–23.1)	2333.4 (p < 0.001)	97.5 (97.1–97.8)	0.0–67.7
Netherlands	59	10,596	1.0–77.0	11.2	14.0 (10.9–17.4)	652.5 (p < 0.001)	91.1 (89.3–92.6)	0.0–43.5
Norway	22	2775	0.0–37.0	13.4	12.4 (8.5–16.9)	165.7 (p < 0.001)	87.3 (82.1–91.0)	0.1–36.7
Poland	18	2871	4.0–12.9	8.0	8.3 (7.0–9.7)	28.9 (p = 0.035)	41.2 (0.0–66.4)	4.5–13.0
Romania	12	1070	0.0–38.3	14.6	13.1 (7.4–20.1)	73.7 (p < 0.001)	85.1 (75.6–90.9)	0.0–44.7
Russian Federation	18	4829	1.4–41.3	16.7	14.8 (9.6–20.8)	461.5 (p < 0.001)	96.3 (95.2–97.2)	0.0–46.8
Spain	24	5519	0.0–40.8	4.0	6.3 (3.2–10.3)	532.5 (p < 0.001)	95.7 (94.5–96.6)	0.0–34.9
Sweden	53	12,103	0.9–40.0	18.0	18.2 (15.4–21.3)	967.2 (p < 0.001)	94.6 (93.6–95.5)	2.6–43.1
Switzerland	13	4150	10.6–31.8	18.1	19.1 (16.4–21.9)	43.0 (p < 0.001)	72.1 (51.3–84.1)	10.2–29.9
Turkey	32	4674	2.1–62.9	8.2	13.5 (8.2–19.8)	1130.1 (p < 0.001)	97.3 (96.7–97.7)	0.0–58.0
UK	46	25,381	0.0–33.9	6.2	6.7 (5.3–8.2)	614.8 (p < 0.001)	92.7 (91.1–94.0)	0.4–19.0
Other^d^	27	13,128	3.4–26.0	11.3	10.8 (8.6–13.1)	344.1 (p < 0.001)	92.4 (90.1–94.2)	2.1–24.8
European subregion/country								
Eastern Europe	90	16,897	0.0–41.3	8.5	9.6 (7.8–11.5)	1546.0 (p < 0.001)	94.2 (93.4–95.0)	0.0–32.2
Southern Europe	121	22,635	0.0–95.0	9.4	12.2 (9.6–15.1)	3559.1 (p < 0.001)	96.6 (96.3–96.9)	0.0–50.8
Western Europe	174	51,372	0.0–77.0	12.5	13.2 (12.0–14.4)	1764.9 (p < 0.001)	90.2 (89.0–91.2)	2.2–30.8
Northern Europe	176	52,064	0.0–51.7	14.0	13.5 (12.0–15.1)	4421.1 (p < 0.001)	96.0 (95.7–96.4)	0.5–37.9
Israel, Turkey, and mixed countries	65	11,989	0.0–62.9	8.1	11.3 (8.5–14.4)	1433.3 (p < 0.001)	95.5 (94.8–96.1)	0.0–42.5
Age group								
<20 years	45	7346	0.0–78.6	4.5	5.9 (3.5–8.8)	842.6 (p < 0.001)	94.8 (93.7–95.6)	0.0–32.6
20–29 years	109	23,057	1.4–67.8	9.0	10.7 (9.2–12.4)	1407.1 (p < 0.001)	92.3 (91.2–93.3)	0.4–30.7
30–39 years	92	13,291	0.0–68.4	14.2	15.0 (12.6–17.5)	1410.2 (p < 0.001)	93.5 (92.6–94.4)	0.4–42.2
40–49 years	34	5939	2.6–85.7	15.0	15.8 (11.3–20.8)	815.8 (p < 0.001)	96.0 (95.1–96.7)	0.0–50.7
50–59 years	19	3814	4.6–29.6	14.2	13.3 (10.5–16.3)	117.1 (p < 0.001)	84.6 (77.3–89.6)	3.3–28.2
≥60 years	21	3192	0.0–60.9	12.3	15.5 (9.7–22.3)	255.8 (p < 0.001)	92.2 (89.4–94.2)	0.0–53.0
Mixed	306	98,318	0.0–95.0	11.9	12.7 (11.4–14.0)	7650.8 (p < 0.001)	96.0 (95.8–96.3)	0.0–39.3
Year of data collection category								
<1995	166	46,557	0.0–85.7	11.2	12.6 (10.7–14.6)	5487.4 (p < 0.001)	97.0 (96.7–97.2)	0.0–44.8
1995–2005	373	98,318	0.0–95.0	11.9	12.9 (11.8–14.0)	6746.2 (p < 0.001)	94.5 (94.1–94.8)	0.2–37.7
>2005	87	16,750	0.0–41.3	8.8	9.8 (8.2–11.6)	884.8 (p < 0.001)	90.3 (88.6–91.7)	0.2–29.2
Year of publication category								
<2000	91	24,236	0.0–85.7	14.0	15.5 (12.3–18.9)	4109.4 (p < 0.001)	97.8 (97.6–98.0)	0.0–54.8
2000–2010	383	94,471	0.0–95.0	11.0	12.0 (11.0–13.1)	6442.2 (p < 0.001)	94.1 (93.7–94.4)	0.1–36.4
>2010	152	36,250	0.0–43.6	10.9	11.3 (9.9–12.9)	2328.2 (p < 0.001)	93.5 (92.8–94.2)	0.3–32.0
All studies	626	154,957	0.0–95.0	11.3	12.4 (11.5–13.3)	13,127.0 (p < 0.001)	95.2 (95.0–95.5)	0.0–38.8

Abbreviations: CI = Confidence interval, HSV-2 = Herpes simplex virus type 2, UK = United Kingdom of Great Britain and Northern Ireland.

a Q: The Cochran's Q statistic is a measure assessing the existence of heterogeneity in seroprevalence.

b I^2^: A measure that assesses the magnitude of between-study variation that is due to actual differences in seroprevalence across studies, rather than sampling variation.

c Prediction interval: A measure that estimates the distribution (95% interval) of true seroprevalence around the estimated mean.

d Other countries: Denmark, Greece, Serbia, Slovakia, Slovenia, and multi-country studies.

### Quality assessments

The quality assessments of diagnostic methods excluded 69 publications due to potential validity issues in diagnostic assays (Fig. 1). Results of the quality assessments of included seroprevalence measures are summarized in Table S7. Out of 294 studies, 210 (71.4%) were of high precision, whereas 43 (14.6%) were of low ROB in the sampling method domain, and 18 (6.1%) were of low ROB in the response rate domain. Eighty-four (28.6%) studies were of low precision, whereas 251 studies (85.4%) were of high ROB in the sampling method domain, and 24 (8.2%) were of high ROB in the response rate domain. Six (2.0%) studies were of low ROB in both quality domains, while 15 (5.1%) studies were of high ROB in both quality domains. For 252 (85.7%) studies, the ROB assessment for the response rate domain was “unclear.” Notably, in the meta-regressions for HSV-2 seroprevalence (note below), only the ROB in the sampling method domain was statistically significantly associated with HSV-2 seroprevalence whereas precision and ROB response rate domain were not. The effect of sampling method was also relatively small.

### Estimates of pooled mean HSV-2 seroprevalence

Table 1 summarizes the pooled mean HSV-2 seroprevalence by sex across at-risk populations. Among general populations, the pooled mean was 14.0% (95% CI: 12.8–15.3%) in women and 8.2% (95% CI: 7.2–9.2%) in men. The pooled mean was highest at 63.2% (95% CI: 55.5–70.6%) among FSWs, 46.0% (95% CI: 40.1–51.8%) among people living with HIV and people in HIV discordant couples, and 27.8% (95% CI: 17.5–39.4%) among MSM. Among STI clinic attendees and symptomatic populations, the pooled mean was 25.5% (95% CI: 20.8–30.5%) in women and 17.4% (95% CI: 13.6–21.6%) in men.

Table 2 summarizes the pooled mean HSV-2 seroprevalence among different subpopulations within the general populations. Across age groups, the pooled mean increased from 5.9% (95% CI: 3.5–8.8%) among <20 years old individuals to 15.5% (95% CI: 9.7–22.3%) among ≥60 years old individuals. By country, the pooled mean was lowest in Hungary at 3.6% (95% CI: 2.0–5.5%) and highest in Bulgaria and Switzerland at 19.1% (95% CI: 12.7–26.4%) and 19.1% (95% CI: 16.4–21.9%), respectively.

Heterogeneity was evident in all meta-analyses (p-value < 0.01) with wide prediction intervals (Tables 1 and 2). The prediction interval estimates the distribution (95% interval) of true seroprevalence around the estimated mean. The 95% CI quantifies the uncertainty in the mean seroprevalence. Since I^2^ measures the magnitude of between-study variation that is due to true differences in seroprevalence across studies rather than sampling variation, an I^2^ >50% indicates that heterogeneity was due to true differences in seroprevalence rather than sampling variation. Forest plots confirmed substantial heterogeneity in stratified seroprevalence measures (Fig. S1).

### Predictors of HSV-2 seroprevalence and sources of between-study heterogeneity

Tables 3 and S8 show results of univariable and multivariable meta-regression analyses for HSV-2 seroprevalence. All four multivariable regression models generated similar results and explained about 40% of seroprevalence variation.

**Table 3:** Univariable and multivariable meta-regression analyses for HSV-2 seroprevalence in Europe, using the year of data collection as the temporal variable instead of the year of publication.

			Outcome measures	Sample size	Univariable analysis				Multivariable analysis^a^			
									Model 1^b^: Time as a categorical variable model		Model 2^c^: Time as a linear variable model	
			Total n	Total N	*RR* (95% CI)	p-value	LR test p-value^d^	Adjusted R^2^ (%)	*ARR* (95% CI)	p-value	*ARR* (95% CI)	p-value
Population characteristics	Population type	General populations	626	154,957	1.00	-	<0.001	27.72	1.00	-	1.00	-
		Intermediate-risk populations^e^	12	3119	0.93 (0.61–1.44)	0.751			1.06 (0.71–1.59)	0.768	1.06 (0.71–1.60)	0.776
		FSWs	16	2047	5.13 (3.58–7.33)	<0.001			4.20 (3.01–5.86)	<0.001	4.55 (3.25–6.36)	<0.001
		MSM	12	7011	1.99 (1.32–3.00)	<0.001			2.66 (1.80–3.93)	<0.001	2.52 (1.70–3.73)	<0.001
		STI clinic attendees and symptomatic populations^f^	74	19,487	2.07 (1.73–2.47)	0.001			1.92 (1.61–2.29)	<0.001	1.90 (1.59–2.27)	<0.001
		People living with HIV and people in HIV discordant couples	35	3660	3.69 (2.88–4.72)	<0.001			3.41 (2.68–4.33)	<0.001	3.37 (2.65–4.30)	<0.001
		Infertility clinic attendees and women with ectopic pregnancies	7	766	0.96 (0.53–1.72)	0.884			0.93 (0.53–1.61)	0.791	0.99 (0.57–1.73)	0.972
		Other populations	31	27,929	1.70 (1.30–2.22)	<0.001			1.71 (1.32–2.22)	<0.001	1.49 (1.16–1.92)	0.002
	Age group	<20 years	48	7799	1.00	-	<0.001	8.41	1.00	-	1.00	-
		20–29 years	117	28,019	1.77 (1.29–2.41)	<0.001			1.80 (1.38–2.34)	<0.001	1.78 (1.37–2.32)	<0.001
		30–39 years	98	19,576	2.55 (1.85–3.51)	<0.001			2.70 (2.06–3.54)	<0.001	2.67 (2.03-3.51)	<0.001
		40–49 years	35	13,743	2.35 (1.59–3.46)	<0.001			2.79 (2.01–3.89)	<0.001	2.76 (1.98–3.84)	<0.001
		50–59 years	19	3814	2.14 (1.35–3.39)	0.001			2.58 (1.75–3.81)	<0.001	2.66 (1.80–3.93)	<0.001
		≥60 years	22	9220	2.57 (1.63–4.03)	<0.001			2.60 (1.76–3.83)	<0.001	2.62 (1.77–3.87)	<0.001
		Mixed	474	136,805	2.73 (2.06–3.61)	<0.001			2.01 (1.57–2.57)	<0.001	2.05 (1.60–2.62)	<0.001
	Sex	Women	437	100,759	1.00	-	<0.001	6.10	1.00	-	1.00	-
		Men	238	55,096	0.69 (0.60–0.79)	<0.001			0.65 (0.57–0.73)	<0.001	0.65 (0.58–0.74)	<0.001
		Mixed	138	63,121	1.26 (1.07–1.49)	0.005			0.98 (0.84–1.14)	0.777	0.98 (0.84–1.14)	0.798
	European subregion	Eastern Europe	101	17,863	1.00	-	<0.001	3.19	1.00	-	1.00	-
		Southern Europe	164	31,963	1.52 (1.22–1.89)	<0.001			1.22 (1.01-1.48)	0.039	1.18 (0.98–1.43)	0.086
		Western Europe	225	85,317	1.68 (1.37–2.07)	<0.001			1.37 (1.14–1.63)	0.001	1.33 (1.12–1.59)	0.002
		Northern Europe	233	67,636	1.51 (1.23–1.85)	<0.001			1.23 (1.03–1.48)	0.023	1.16 (0.96–1.39)	0.121
		Mixed regions	90	16,197	1.26 (0.98–1.62)	0.076			0.91 (0.74–1.13)	0.400	0.90 (0.73–1.12)	0.344
	Country's income	UMIC	87	15,429	1.00	-	0.326	0.03	-	-	-	-
		HIC	721	202,919	1.03 (0.84–1.26)	0.769			-	-	-	-
		Mixed	5	628	1.80 (0.84–3.89)	0.132			-	-	-	-
Study methodology characteristics	Assay type	Western Blot	80	23,545	1.00	-	0.541	0.00	-	-	-	-
		ELISA	725	190,632	0.99 (0.80–1.24)	0.940			-	-	-	-
		Monoclonal antibody	8	4799	1.39 (0.74–2.60)	0.306			-	-	-	-
	Sample size^g^	<200	106	6947	1.00	-	0.013	1.24	1.00	-	1.00	-
		≥200	707	212,029	0.78 (0.65–0.95)	0.013			1.00 (0.85–1.18)	0.979	0.98 (0.83–1.16)	0.854
	Sampling method	Probability-based	210	62,803	1.00	-	<0.001	3.04	1.00	-	1.00	-
		Non-probability-based	603	156,173	1.31 (1.15–1.51)	<0.001			1.25 (1.09–1.44)	0.001	1.20 (1.05–1.38)	0.008
	Response rate	≥80%	41	13,331	1.00	-	0.009	1.09	1.00	-	1.00	-
		<80%	102	28,738	1.06 (0.78–1.44)	0.716			1.01 (0.78–1.3)	0.944	1.02 (0.79–1.32)	0.869
		Unclear	670	176,907	0.82 (0.62–1.07)	0.135			0.80 (0.64–1.00)	0.053	0.82 (0.65–1.02)	0.078
Temporal variables	Year of data collection category	<1995	226	61,250	1.00	-	0.268	0.09	1.00	-	-	-
		1995–2005	465	111,095	1.07 (0.93–1.23)	0.362			1.12 (1.00–1.27)	0.056	-	-
		>2005	122	46,631	0.93 (0.76–1.13)	0.454			0.82 (0.69–0.97)	0.021	-	-
	Year of data collection		813	218,976	0.99 (0.99–1.00)	0.181	0.181	0.13	-	-	0.99 (0.98–1.00)	0.013

Abbreviations: *ARR* = Adjusted risk ratio, CI = Confidence interval, ELISA = Enzyme-linked immunosorbent type-specific assay, FSWs = Female sex workers, HIC = High-income countries, HIV = Human immunodeficiency virus, HSV-2 = Herpes simplex virus type 2, LR = Likelihood ratio, MSM = Men who have sex with men, *RR* = Risk ratio, STI = Sexually transmitted infection, UMIC = Upper-middle-income countries.

a Models 1 and 2 used only year of data collection as the temporal variable due to collinearity between year of publication and year of data collection. The same analyses using year of publication are presented in Table S8.

b Variance explained by multivariable model 1 (adjusted *R*^*2*^) = 41.28%.

c Variance explained by multivariable model 2 (adjusted *R*^*2*^) = 40.51%.

d Factors in the univariable analyses with a p-value <0.1 were included in the multivariable analysis.

e Intermediate-risk populations include populations who presumably have frequent sexual contacts with populations engaging in high sexual risk behaviour and have therefore a higher risk of exposure to HSV-2 than the general population. These comprise prisoners, people who inject drugs, and truck drivers, among others.

f Symptomatic populations include patients with STI clinical manifestations.

g Sample size denotes the sample size of each study population found in the original publication.

The model including year of data collection as a continuous linear variable explained 40.5% of seroprevalence variation (Table 3). Population type variable alone explained most variation (27.7%), followed by age group variable (8.4%). Compared to general populations, HSV-2 seroprevalence was 4.55-fold (95% CI: 3.25–6.36) higher among FSWs, 3.37-fold (95% CI: 2.65–4.30) higher among people living with HIV and people in HIV discordant couples, 2.52-fold (95% CI: 1.70–3.73) higher among MSM, and 1.90-fold (95% CI: 1.59–2.27) higher among STI clinic attendees and symptomatic populations.

Seroprevalence increased rapidly for young persons, but plateaued by 30–39 years of age. Seroprevalence in men was 0.65-fold (95% CI: 0.58–0.74) lower compared to women. Compared to Eastern Europe, HSV-2 seroprevalence was highest in Western Europe (1.33-fold (95% CI: 1.12–1.59) higher). HSV-2 seroprevalence decreased by 0.99-fold (95% CI: 0.98–1.00) per calendar year, indicating that seroprevalence has been declining at a rate of 1% per calendar year.

Among study methodology characteristics, assay type, sample size, and response rate were not associated with HSV-2 seroprevalence; however, sampling method was. Compared to studies using probability-based sampling, seroprevalence was 1.20-fold (95% CI: 1.05–1.38) higher in studies using non-probability-based sampling.

Subgroup meta-regression analyses were conducted including only studies among general populations (Table S9). The analyses confirmed similar findings to those of the main analyses including all population types.

### Overview and meta-analyses of HSV-2 detection in genital ulcer disease and in genital herpes

Tables 4 and S10 summarize extracted proportions of HSV-2 detection in clinically diagnosed GUD and in laboratory-confirmed genital herpes. The proportion of HSV-2 detection in GUD (n = 15) ranged from 2.0 to 48.1% with a median of 20.9% and a pooled mean of 22.0% (95% CI: 15.3–29.6%). The proportion of HSV-2 detection in genital herpes (n = 183) ranged from 12.0 to 100.0% with a median of 66.7% and a pooled mean of 66.0% (95% CI: 62.9–69.1%). The meta-analyses showed heterogeneity in proportions and wide prediction intervals (Table 4). Forest plots confirmed substantial heterogeneity in the stratified proportions of HSV-2 detection in GUD and in genital herpes (Fig. S2).

**Table 4:** Pooled proportions of HSV-2 virus detection in clinically diagnosed GUD and in laboratory-confirmed genital herpes in Europe.

Population type	Outcome measures	Sample size	Proportion of HSV-2 detection (%)		Pooled proportion of HSV-2 detection (%)	Heterogeneity measures		
	Total n	Total N	Range	Median	Mean (95% CI)	Q^a^ (p-value)	I^2^^b^ (%) (95% CI)	Prediction Interval^c^ (%)
Patients with clinically diagnosed GUD								
Sex								
Women^d^	2	201	-	-	23.9 (17.4–31.1)	-	-	-
Men^d^	2	153	-	-	17.6 (11.8–24.1)	-	-	-
Mixed	11	3819	2.0–48.1	22.3	22.2 (13.3–32.6)	282.9 (p < 0.001)	96.5 (95.0–97.5)	0.0–66.0
All patients with GUD	15	4173	2.0–48.1	20.9	22.0 (15.3–29.6)	300.1 (p < 0.001)	95.3 (93.6–96.6)	1.2–57.2
Patients with laboratory-confirmed genital herpes								
Sex								
Women	51	7368	12.0–92.0	54.5	55.4 (50.5–60.2)	692.0 (p < 0.001)	92.8 (91.3–94.0)	24.3–84.4
Men	48	5190	25.0–96.0	70.7	69.8 (65.2–74.2)	580.7 (p < 0.001)	91.9 (90.1–93.4)	38.6–93.5
Mixed	84	10,765	16.7–100	71.4	69.7 (64.6–74.6)	2227.9 (p < 0.001)	96.3 (95.8–96.7)	23.0–99.7
Age								
<25 years	7	1638	32.8–70.0	42.5	42.4 (35.3–49.7)	25.3 (p < 0.001)	76.3 (50.2–88.7)	22.0–64.2
≥25 years	15	1865	44.3–90.3	60.0	62.1 (56.0–68.0)	57.5 (p < 0.001)	75.7 (59.8–85.3)	38.7–83.0
Mixed	161	19,820	12.0–100	68.2	67.2 (63.8–70.6)	3317.0 (p < 0.001)	95.2 (94.7–95.6)	25.3–97.5
Genital herpes episode status								
First episode	57	9135	12.0–92.8	52.0	52.3 (47.7–56.8)	643.6 (p < 0.001)	91.3 (89.5–92.8)	21.7–82.0
Recurrent episode	13	1907	22.7–96.0	85.0	83.2 (73.2–91.2)	134.9 (p < 0.001)	91.1 (86.6–94.1)	37.9–100
Unspecified status	113	12,281	24.8–100	71.0	70.6 (66.9–74.1)	1863.1 (p < 0.001)	94.0 (93.2–94.7)	32.7–97.1
European subregion/country								
Southern Europe^e^	20	1881	24.8–100	77.6	75.3 (64.4–84.9)	636.3 (p < 0.001)	97.0 (96.2–97.6)	23.4–100
Western Europe	11	1016	45.6–94.2	77.3	74.2 (63.3–83.9)	96.9 (p < 0.001)	89.7 (83.6–93.5)	32.5–99.6
Northern Europe	142	20,141	12.0–96.0	67.1	66.1 (62.8–69.4)	2776.4 (p < 0.001)	94.9 (94.4–95.4)	27.7–95.4
Israel	10	285	25.0–52.4	35.0	33.5 (27.9–39.2)	6.5 (p = 0.689)	0.0 (0.0–62.4)	27.0–40.2
Sample size								
<200	49	2032	12.0–94.3	58.7	57.1 (50.1–64.0)	512.6 (p < 0.001)	90.6 (88.5–92.4)	13.6–95.0
≥200	134	21,291	22.7–100	68.3	68.8 (65.5–72.1)	3423.9 (p < 0.001)	96.1 (95.7–96.5)	30.2–96.6
Year of publication category								
<2000	50	4179	25.0–100	71.0	71.6 (65.9–76.9)	754.2 (p < 0.001)	93.5 (92.2–94.6)	30.4–98.6
2000–2010	87	13,125	12.0–100	64.5	64.8 (59.6–69.8)	1818.2 (p < 0.001)	95.3 (94.6–95.8)	19.2–98.3
>2010	46	6019	16.7–92.8	63.8	61.7 (57.1–66.2)	805.8 (p < 0.001)	94.4 (93.3–95.4)	32.2–87.3
Year of data collection category								
<1995	69	8285	25.0–100	68.2	68.2 (63.0–73.2)	1728.6 (p < 0.001)	96.1 (95.5–96.6)	25.2–98.2
1995–2005	68	9692	12.0–100	72.7	67.6 (61.8–73.2)	1355.0 (p < 0.001)	95.1 (94.3–95.7)	21.5–99.2
>2005	46	5346	16.7–92.8	62.2	59.7 (55.1–64.2)	456.1 (p < 0.001)	90.1 (87.7–92.1)	31.0–85.3
All patients with genital herpes	183	23,323	12.0–100.0	66.7	66.0 (62.9–69.1)	3944.1 (p < 0.001)	95.4 (95.0–95.8)	25.2–96.5

Abbreviations: CI = Confidence interval, GUD = Genital ulcer disease, HSV-2 = Herpes simplex virus type 2.

a Q: The Cochran's Q statistic is a measure assessing the existence of heterogeneity in proportion of HSV-2 virus detection.

b I^2^: A measure assessing the magnitude of between-study variation that is due to true differences in proportion of HSV-2 virus detection across studies, rather than sampling variation.

c Prediction interval: A measure quantifying the distribution 95% interval of true proportions of HSV-2 virus detection around the estimated pooled mean.

d No meta-analysis was done due to the small number of studies (n < 3).

e Southern Europe includes one measure from Eastern Europe.

### Predictors of HSV-2 detection in genital herpes and sources of between-study heterogeneity

Tables 5 and S11 show results of univariable and multivariable meta-regression analyses for the proportion of HSV-2 detection in genital herpes. All four multivariable regression models generated similar results and explained over 50% of the variation in this proportion.

**Table 5:** Univariable and multivariable meta-regression analyses for proportion of HSV-2 virus detection in laboratory-confirmed genital herpes in Europe, using the year of data collection as the temporal variable instead of the year of publication.

		Outcome measures	Samples	Univariable analysis				Multivariable analysis^a^			
								Model 1^b^: Time as a categorical variable model		Model 2^c^: Time as a linear variable model	
		Total n	Total N	*RR* (95%CI)	p-value	LR test p-value^d^	Adjusted R^2^ (%)	*ARR* (95%CI)	p-value	*ARR* (95%CI)	p-value
Age group	<25 years	7	1638	1.00	-	0.005	10.09	1.00	-	1.00	-
	≥25 years	15	1865	1.41 (1.05–1.88)	0.021			1.53 (1.23–1.89)	<0.001	1.54 (1.25–1.89)	<0.001
	Mixed	161	19,820	1.50 (1.18–1.93)	0.001			1.15 (0.94–1.40)	0.180	1.18 (0.98–1.42)	0.087
Sex	Women	51	7368	1.00	-	0.001	10.39	1.00	-	1.00	-
	Men	48	5190	1.23 (1.08–1.39)	0.001			1.21 (1.10–1.33)	<0.001	1.21 (1.10–1.32)	<0.001
	Mixed	84	10,765	1.21 (1.08–1.35)	0.001			1.12 (1.01–1.23)	0.028	1.07 (0.97–1.18)	0.180
Genital herpes episode status	First episode	57	9135	1.00	-	<0.001	27.34	1.00	-	1.00	-
	Recurrent episode	13	1907	1.54 (1.30–1.82)	<0.001			1.51 (1.30–1.75)	<0.001	1.52 (1.32–1.75)	<0.001
	Unspecified status	113	12,281	1.31 (1.19–1.44)	<0.001			1.39 (1.26–1.53)	<0.001	1.33 (1.21–1.46)	<0.001
European subregion/country	Southern Europe^e^	20	1881	1.00	-	<0.001	13.30	1.00	-	1.00	-
	Western Europe	11	1016	1.01 (0.81–1.26)	0.909			1.13 (0.95–1.35)	0.164	1.08 (0.91–1.28)	0.373
	Northern Europe	142	20,141	0.91 (0.79–1.05)	0.185			0.94 (0.83–1.06)	0.311	0.88 (0.78–0.99)	0.030
	Israel	10	285	0.49 (0.37–0.64)	<0.001			0.44 (0.35–0.56)	<0.001	0.44 (0.35–0.56)	<0.001
Sample size^f^	<200	49	2032	1.00	-	0.014	0.53	1.00	-	1.00	-
	≥200	134	21,291	1.15 (1.03–1.29)	0.014			1.09 (1.00–1.19)	0.054	1.14 (1.04–1.24)	0.004
Year of data collection category	<1995	69	8285	1.00	-	0.104	5.03	1.00	-	-	-
	1995–2005	68	9692	1.01 (0.90–1.13)	0.863			0.97 (0.88–1.06)	0.507	-	-
	>2005	46	5346	0.89 (0.79–1.01)	0.067			0.86 (0.77–0.96)	0.010	-	-
Year of data collection		183	23,323	0.99 (0.99–1.00)	0.001	0.001	11.12	-	-	0.99 (0.99–1.00)	0.001

Abbreviations: ARR = Adjusted risk ratio, CI = Confidence interval, HSV-2 = Herpes simplex virus type 2, LR = Likelihood ratio, RR = Risk ratio.

a Models 1 and 2 used only year of data collection as the temporal variable due to collinearity between year of publication and year of data collection. The same analyses using year of publication are presented in Table S11.

b Variance explained by the final multivariable model 1 (adjusted *R*^*2*^) = 53.89%.

c Variance explained by the final multivariable model 2 (adjusted *R*^*2*^) = 58.15%.

d Factors in the univariable analyses with a p-value <0.1 were included in the multivariable analysis.

e Southern Europe includes one measure from Eastern Europe.

f Sample size denotes the sample size of the study population found in the original publication.

The model including year of data collection as a continuous linear variable explained 58.2% of the genital herpes proportion variation (Table 5). Genital herpes episode status (first *versus* recurrent episodes) explained most variation (27.3%), followed by European subregion, sex, and age, each at just over 10% of the variation.

Compared to those with first episode genital herpes, the proportion of HSV-2 detection was 1.52-fold (95% CI: 1.32–1.75) higher in those with recurrent genital herpes (Table 5). Compared to <25 years old individuals, the proportion of HSV-2 detection in genital herpes was 1.54-fold (95% CI: 1.25–1.89) higher in ≥25 years old individuals. Men had a 1.21-fold (95% CI: 1.10–1.32) higher proportion of HSV-2 detection compared to women. Israel had the lowest proportion of HSV-2 detection. The proportion of HSV-2 detection decreased by 0.99-fold (95% CI: 0.99–1.00) per calendar year, indicating that seroprevalence has been declining at a rate of 1% per calendar year.

Subgroup meta-regression analyses were conducted for each of studies of first episode genital herpes and studies of recurrent genital herpes (not shown). Both analyses suggested declining HSV-2 contribution to both, but the effects did not reach statistical significance, perhaps due to the smaller number of studies included in the analyses.

### Neonatal herpes

Neonatal herpes is a rare condition but results in significant morbidity and mortality.^43^ Incidence of neonatal herpes infection worldwide during 2010–2015 was estimated using mathematical modelling at 10.3 cases per 100,000 live births.^44^ During the same duration, incidence in Europe was estimated at 8.9 cases per 100,000 live births, just below the global incidence rate.^44^ By type of infection, incidence in Europe was estimated at 5.2 cases per 100,000 live births for HSV-1 and at 3.8 cases per 100,000 live births for HSV-2.^44^

Tens of studies have documented occurrence of neonatal herpes for over 4 decades in Europe in countries such as Denmark,^45^ France,^43^ Germany,^46^^,^^47^ Ireland,^48^ Israel,^49^, ^50^, ^51^ the Netherlands,^52^, ^53^, ^54^, ^55^, ^56^, ^57^, ^58^ Poland,^59^^,^^60^ Russia,^61^ Spain,^62^ Sweden,^63^, ^64^, ^65^ Switzerland,^66^ Turkey,^67^ and the United Kingdom (UK).^48^^,^^68^, ^69^, ^70^, ^71^, ^72^, ^73^, ^74^, ^75^, ^76^, ^77^, ^78^ However, surveillance for this condition remains overall limited with no data being reported for the majority of countries in Europe. Reporting quality also varies widely from reports indicating sporadic cases to well-conducted epidemiologic studies based on surveillance systems and reporting estimates for incidence at a national level, such as in the Netherlands^52^, ^53^, ^54^, ^55^, ^56^, ^57^ and the UK.^48^^,^^69^^,^^71^, ^72^, ^73^, ^74^, ^75^

In the Netherlands, incidence of neonatal herpes during 1981–1985, 1987–1991, 1992–1998, 1999–2005, 2006–2011, and 2012–2015 was reported at 2.9,^52^ 2.0,^53^ 2.4,^54^ 3.2,^55^ 4.7,^56^ and 4.8^57^ cases, respectively, per 100,000 live births. The studies suggested a trend of slowly increasing neonatal herpes over time. Of 179 cases reported during 1981–2015, 117 (65.4%) were due to HSV-1, 34 (19.0%) were due to HSV-2, and the remaining 28 (15.6%) had unknown HSV type.^52^, ^53^, ^54^, ^55^, ^56^, ^57^

In the UK, incidence of neonatal herpes during 1986–1991, 2004–2015, 2006–2012, and 2012, was reported at 1.7,^75^ 11.9,^70^ 17.5,^68^ and 9.6^69^ cases, respectively, per 100,000 live births. In the UK and Ireland during 2019–2021, incidence was estimated at 6.9 cases per 100,000 live births.^48^ Overall, studies suggested an increasing trend. Of 59 reported cases in the UK during 2019–2021, 29 (49.2%) were due to HSV-1, 25 (42.4%) were due to HSV-2, and the remaining 5 (8.5%) had unknown HSV type.^48^

In Denmark, incidence of neonatal herpes was reported during 1977–1984 and 1984–1991 at 2.4^45^ and 4.6^45^ cases, respectively, per 100,000 live births. In Germany, it was reported during 2017–2018 at 2.4 cases per 100,000 live births.^46^ In Israel, it was reported during 2001–2007 at 8.4 cases per 100,000 live births.^51^ In Poland, it was reported during 2014–2019 at 69.0 cases per 100,000 live births.^59^ Poland also reported an outbreak of neonatal herpes in a hospital setting that affected 11 new-borns.^60^ In Sweden, it was reported during 1989–2000 at 7.1 cases per 100,000 live births.^63^ In Switzerland, it was reported during 2002–2008 at 1.6 cases per 100,000 live births.^66^ Examination of the trend in all available incidence measures suggested an increasing trend of neonatal herpes in Europe (Fig. S3).

### Role of HSV-2 in HIV transmission

Direct, observational studies of the contribution of HSV-2 to HIV infections in Europe are lacking. A systematic review done in 2017^8^ of the effect of HSV-2 infection on HIV acquisition (i.e., susceptibility) identified only one such study, a nested case–control study in MSM in the Netherlands.^79^ This study reported data from which the unadjusted odds ratio (OR) for HIV incidence in those with HSV-2 infection compared to those without could be calculated. The unadjusted OR was calculated to be 4.3 (95% CI: 1.9–9.9) for prevalent (existing) HSV-2 infection, and 2.2 (95% CI: 0.4–12.2) for incident (recently acquired) HSV-2 infection. However, for the OR for incident HSV-2 on HIV, it was not known whether HSV-2 infection preceded HIV or not.

By pooling all adjusted risk ratio (ARR) study estimates, which met certain quality criteria (including: HSV-2 infection known to have preceded HIV infection), from any setting worldwide, the 2017 systematic review found an ARR of 2.7 (95% CI: 2.2–3.4; n = 22) for prevalent HSV-2 on HIV acquisition in general populations, and an ARR of 4.7 (95% CI: 2.2–10.1; n = 6) for incident HSV-2 on HIV acquisition in general populations. The RR for HIV transmission (i.e., infectiousness) due to HSV-2 (any setting) has been estimated to be 1.3 (range: 1.0–1.9).^80^ Studies (done in settings outside of Europe) suggest that during ulcerative episodes the risks of HIV acquisition and transmission are even higher.^81^^,^^82^

A recent epidemiological analysis of the population attributable fraction (PAF) of sexually-acquired incident HIV attributable to HSV-2 among 15–49 years old in the general populations was recently completed for all six WHO regions,^9^ including Europe. Here, the classic epidemiological formula for the PAF was used to apply the pooled ARRs of HSV-2 on HIV acquisition from the 2017 systematic review^8^ to HSV-2 infection data for Europe for 2016. An overall PAF of 11.6% (95% uncertainty interval (UI): 7.0–19.4%) was calculated for Europe, of which 10.6% (95% UI: 5.9–18.3%) was estimated to be due to prevalent HSV-2 and 1.0% (95% UI: 0.4–2.2%) due to incident HSV-2. These estimates were similar to those for other WHO regions, with the exception of the WHO African and Americas regions, which had higher estimated PAFs due to higher HSV-2 infection rates. There is the potential for residual confounding in the pooled ARR estimates, which may have inflated PAF estimates, however only the contribution of HSV-2 to increased HIV susceptibility was considered (and not also transmissibility), potentially under-estimating the PAF.

A subsequent model-based analysis of the PAF, which included the WHO European region,^80^ found similar PAF estimates to those obtained from the classic formula (11.2% [95% UI: 7.9–13.8%] for Europe 2009–2018), and even higher (26.8% [95% UI: 16.2–35.8%] for Europe for 2009–2018) PAF estimates when additionally considering the contribution of HSV-2 to HIV transmissibility.

## Discussion

HSV-2 seroprevalence in the adult general population of Europe averaged at about 12% over the last three decades, similar to that found in Asia,^17^ but lower than that in Africa at 37%,^18^ in Latin America and the Caribbean (LAC) at 21%,^19^ and in the United States of America (USA), at about 15%.^83^, ^84^, ^85^, ^86^ HSV-2 seroprevalence has been declining in Europe at a rate of 1% per calendar year over the last three decades. Similar declines were observed, but at a higher rate of 2% per calendar year in Africa,^18^ Asia,^17^ LAC,^19^ and the USA.^83^, ^84^, ^85^, ^86^ Since HSV-2 seroprevalence is a proxy for population sexual risk behaviour,^20^^,^^41^^,^^42^^,^^87^, ^88^, ^89^ these declines may be explained by less risky sex following recognition of the HIV epidemic,^90^, ^91^, ^92^, ^93^ improved STI awareness,^94^ increasing access to HIV/STI services,^12^^,^^95^ and/or changes in the structure of sexual networks following changes in socio-economic conditions.^17^ Use of antiviral suppressive therapy is unlikely to be substantial in Europe and such therapy, if used, is not likely to be used in the long-term. Therefore, use of such therapy may not have appreciably affected HSV-2 transmission to explain the declining trends.

These results demonstrated generic patterns in HSV-2 epidemiology that are also observed in other world regions.^17^, ^18^, ^19^^,^^83^, ^84^, ^85^, ^86^ There was strong hierarchy in seroprevalence based on sexual risk behaviour classification, with the highest seroprevalence observed in FSWs and MSM and the lowest in the general populations, similar to what is observed for other STIs.^96^^,^^97^ Men had 35% lower seroprevalence than women, attesting to a higher bio-anatomical susceptibility to this infection among women.^98^^,^^99^ Seroprevalence increased rapidly with age after sexual debut, but plateaued by the mid-30s. There was also evidence for some variability by subregion and country, with Western Europe having the highest seroprevalence.

HSV-2 was the aetiological cause of 22% of clinically diagnosed GUD and 66% of laboratory-confirmed genital herpes. While these rates are considerable and similar to those observed in North America,^100^, ^101^, ^102^ they are lower than those found in Africa,^18^ Asia,^17^ and LAC.^19^ The contribution of HSV-2 infection (as opposed to HSV-1 infection) to genital herpes is also declining at a rate of 1% per calendar year. This decline may reflect not only declining HSV-2 seroprevalence, but also increasing transmission of HSV-1 through oral sex.^103^ Indeed, findings of this study are consistent with findings of a study of similar design for HSV-1 infection that estimated HSV-1's contribution to genital herpes at 34%, increasing by 1% per calendar year.^1^

HSV-2 had a higher detection rate in recurrent genital herpes than in first-episode genital herpes, consistent with more persistent reactivations in the genital tract for HSV-2 than for HSV-1 infection.^104^ Consequently, and since our estimates are for the proportion of HSV-1 versus HSV-2 among people who present to care and receive a diagnosis of symptomatic genital herpes, not among all people with genital HSV-2 or HSV-1 infection, this finding will underestimate the proportion of genital herpes due to HSV-1. HSV-2 was found more common in genital herpes among older individuals and men than among younger individuals and women. This finding may reflect decreasing childhood HSV-1 infection and increasing transmission of HSV-1 through oral sex among younger persons, and especially young women.^101^^,^^103^

Findings suggested an increasing trend of incidence of neonatal herpes in Europe, opposite to the decreasing trend of HSV-2 seroprevalence, perhaps reflecting the increasing trend of HSV-1-caused genital herpes in this region,^1^ and increasing maternal age associated with higher HSV-2 seroprevalence.^44^ Incident HSV-1 appears more readily transmissible to the neonate than HSV-2.^65^ HSV-2 infection appears to facilitate a considerable number of HIV transmissions in Europe, particularly in higher-risk populations where HIV is concentrated, but not as much as its contribution to increasing HIV transmission in Africa.^7^^,^^9^

This study has limitations. No data were available for 27 of the 53 European countries. However, data were available for all large countries. The 26 European countries for which data were available constituted 80.2% of the total population of the European region. Despite the availability of a large number of seroprevalence measures in Europe, larger than those available in other regions,^16^^,^^17^^,^^19^ there are still significant gaps in available data. Most seroprevalence measures were in general populations, but there were not sufficient measures in key populations such as FSWs and MSM. Among the general populations, 23.2% (n = 42) of studies were on pregnant women and 7.7% (n = 14) were on blood donors, but these populations may not be representative of the wider general population.^105^

The majority of studies were conducted between 1995 and 2005, with a declining trend in availability of studies in recent years. A significant proportion of studies was conducted on relatively small samples; 28.6% of studies had a sample size of <200. More studies were conducted in Western and Northern Europe than in Eastern and Southern Europe. Most studies did not use probability-based sampling. Use of diagnostic assays varied over time.

While studies differed by assay type, sample size, sampling method, and response rate, none of these study characteristics, apart from sampling method, had an effect on observed seroprevalence. The effect of sampling method was also relatively small–studies using non-probability sampling reported on average 1.20-fold higher seroprevalence. There was heterogeneity in effect sizes of included studies, but much of this heterogeneity was subsequently explained by the meta-regressions. Publication bias was not assessed due to methodological issues in assessing it for studies pooling proportions.^106^

Most studies of HSV-2 virus detection in genital herpes did not differentiate between first episode and recurrent episodes. This distinction is important considering the different contributions of HSV-2 *versus* HSV-1 infections to first episode *versus* recurrent episodes.^1^^,^^17^^,^^24^ Most studies were conducted before or in 2005 with decreasing number of studies in recent years. Most studies were conducted in Northern Europe with only small number of studies conducted elsewhere in Europe.

Much of the studies of neonatal herpes were conducted in the Netherlands and the UK, with only small number of studies from other countries. More systematic research needs to be conducted to better understand epidemiology of neonatal herpes in Europe. Neonatal herpes is a serious condition with significant mortality and long-term sequela, which necessitates the recontinued surveillance and monitoring of new cases to inform prevention efforts of herpes infections, including HSV-2. Surveillance for neonatal herpes remains limited in Europe with no data being reported for the majority of countries. Only a few countries have estimates for incidence of neonatal herpes based on large national studies.

A strength of this study is the availability of a large volume of HSV-2 data, larger than that found in other regions,^16^^,^^17^^,^^19^ which facilitated an array of analyses. Building on these compiled data, a future direction of this work is to conduct mathematical modelling studies to quantitatively characterize the transitioning epidemiology of HSV-2 in Europe and to estimate its epidemiologic indicators such as incidence, past, present, and future, similar to that done recently for the USA.^83^

## Conclusions

HSV-2 seroprevalence is declining in Europe, but at a relatively slow rate of 1% per calendar year, lower than that in other world regions. A significant proportion of Europe's population is chronically infected with this virus, which causes over 20% of GUD cases and nearly 70% of genital herpes cases in this part of the world. HSV-2 infection appears to facilitate the transmission of a considerable number of HIV infections. Meanwhile, there is an increasing trend of neonatal herpes in Europe, but mostly driven by HSV-1 infection. These findings demonstrate the need to accelerate HSV-2 vaccine development and universal access to sexual and reproductive health services. HSV-2 research and surveillance need also to be expanded by conducting national population-based seroprevalence surveys and monitoring incidence of neonatal herpes and aetiology of GUD and genital herpes cases.

## Contributors

A.A., A.M.M.O., M.N.K., and M.H. conducted the systematic search, data extraction, data analysis, and interpretation of the results. A.A. and A.M.M.O. wrote the first draft of the paper with L.J.A.-R. L.J.A.-R. conceived the study and led the data extraction and analysis and interpretation of the results. K.J.L. contributed to the neonatal herpes review and drafted the contribution of HSV-2 to HIV transmission. All authors contributed to drafting and revising the manuscript.

## Data sharing statement

All relevant data are presented in the manuscript and its Supplementary Material file.

## Declaration of interests

K.J.L. has received funding from GSK and the World Health Organization for work outside of the submitted work. Otherwise all authors declare no competing interests.

## Appendix A Supplementary data

Supplemental Box S1, S2, Figures S1–S3 and Tables S2–S11
