# Supplementary material for: Epidemiology of herpes simplex virus type 2 in Europe: systematic review, meta-analyses, and meta-regressions
Source: Lancet Reg Health Eur. 2022 Dec 12;25:100558. doi: 10.1016/j.lanepe.2022.100558 (PMC9929610; doi:10.1016/j.lanepe.2022.100558)
Supplement: Supplemental Box S1, S2, Figures S1–S3 and Tables S2–S11 [file mmc1.docx]

**Supplementary Material**

**Box S1. Definitions of population type classifications.**

| 1. **General populations** (populations at low risk): these include populations at lower risk of exposure to HSV-2, such as antenatal clinic attendees, blood donors, and pregnant women, among others. 2. **Intermediate-risk populations**: these include populations who presumably have frequent sexual contacts with populations engaging in high sexual risk behaviour and have therefore a higher risk of exposure to HSV-2 than the general population. These comprise prisoners, people who inject drugs, and truck drivers, among others. 3. **Higher-risk populations**: these include populations at high risk of exposure to HSV-2 because of specific sexual risk behaviours such as female sex workers, men who have sex with men, male sex workers, and transgender people, among others. 4. **STI clinic attendees and symptomatic populations**: these include patients attending STI clinics, or have clinical manifestations related to an STI. 5. **People living with HIV and people in HIV discordant couples**: these include populations living with HIV or are in a spousal relationship with an individual living with HIV. 6. **Infertility clinic attendees and women with ectopic pregnancy**: these include patients attending infertility clinics and women suffering from ectopic pregnancies. 7. **Other populations**: these include populations not satisfying above definitions, or populations with an undetermined risk of acquiring HSV-2 infection such as cervical cancer patients and their spouses. |
| --- |

Abbreviations: HIV = Human immunodeficiency virus, HSV-2 = Herpes simplex virus type 2, STI = Sexually transmitted infection.

**Table S1. Preferred Reporting Items for Systematic Reviews and Meta-analyses (PRISMA) checklist.^1^**

**Table S2. Data sources and search criteria for systematically reviewing HSV-2 epidemiology in Europe.**

| **PubMed (last searched February 20, 2022):** |
| --- |
| (Simplexvirus[MeSH] OR Herpes Simplex[MeSH] OR Herpes Genitalis[MeSH] OR Herpes Hominis[Text] OR HSV type-2[Text] OR HSV type 2[Text] OR HSV2[Text] OR HSV-2[Text] OR HSV [Text] OR Human herpes virus[Text] OR Herpes simplex virus type 2[Text] OR Herpes simplex virus type-2[Text] OR herpes simplex virus 2[Text] OR herpes simplex virus-2[Text] OR herpes simplex type 2[Text] OR herpes simplex type-2[Text] OR herpes simplex 2[Text] OR herpes simplex-2[Text] OR Herpesvirus type 2[Text] OR Herpesvirus type-2[Text] OR Herpesvirus 2[Text] OR Herpesvirus-2[Text] OR Herpes virus type 2[Text] OR Herpes virus type-[Text] OR Herpes virus [Text] OR Herpes virus-2[Text] OR genital herpes[Text] OR Herpes Genitalis[Text] OR Stomatitis Herpetic[Text] OR Herpes Labialis[Text]) **AND** (“Europe”[MeSH] OR Greenland[MeSH] “Israel”[MeSH] OR “Turkey”[MeSH] OR “USSR”[MeSH] OR “Cyprus”[MeSH] OR Albania*[Text] OR Andorra*[Text] OR Armenia*[Text] OR Austria*[Text] OR Azerbaijan*[Text] OR Belarus*[Text] OR Belgi*[Text] OR Bosnia*[Text] OR Bosnia and Herzegovina[Text] OR Bulgaria*[Text] OR Croatia*[Text] OR Cypr*[Text] OR Czech Republic[Text] OR Czech*[Text] OR Denmark*[Text] OR Danish[Text] OR Estonia*[Text] OR Finland*[Text] OR Finnish[Text] OR France*[Text] OR French[Text] OR Georgia*[Text] OR German*[Text] OR Gree*[Text] OR Greenlan*[Text] OR Hungar*[Text] OR Iceland*[Text] OR Ireland*[Text] OR Irish[Text] OR Israel*[Text] OR Ital*[Text] OR Kazakh*[Text] OR Kyrgyz*[Text] OR Latvia*[Text] OR Lithuania*[Text] OR Luxembourg*[Text] OR Malta*[Text] OR Monac*[Text] OR Montenegr*[Text] OR Netherlands*[Text] OR Dutch[Text] OR Norway*[Text] OR Norweg*[Text] OR Poland*[Text] OR Polish[Text] OR Portug*[Text] OR Republic of Moldova*[Text] OR Moldov*[Text] OR Romania*[Text] OR Russia*[Text] OR Russian Federation*[Text] OR San Marino*[Text] OR Serbia*[Text] OR Slovakia*[Text] OR Slovenia*[Text] OR Spain*[Text] OR Spanish[Text] OR Swed*[Text] OR Switzerland*[Text] OR Swiss[Text] OR Tajik*[Text] OR Yugoslav*[Text] OR Republic of Macedonia*[Text] OR Macedonia*[Text] OR Turkey*[Text] OR Turkmen*[Text] OR Ukrain*[Text] OR United Kingdom*[Text] OR Great Britain*[Text] OR UK[Text] OR Uzbek*[Text]) |
| **Embase (last searched February 20, 2022):** |
| (exp Herpes simplex/ or exp Herpesviridae/) OR (Herpes simplex or Herpes simplex virus or HSV type-2 or HSV type 2 or HSV2 or HSV-2 or HSV 2 or human herpes virus or Herpes simplex virus type 2 or Herpes simplex virus type-2 or herpes simplex virus 2 or herpes simplex virus-2 or herpes simplex type 2 or herpes simplex type-2 or herpes simplex 2 or herpes simplex-2 or Herpesvirus type 2 or Herpesvirus type-2 or Herpesvirus 2 or Herpesvirus-2 or Herpes virus type 2 or Herpes virus type-2 or Herpes virus 2 or Herpes virus-2 or genital herpes or Herpes Genitalis or herpes labialis or herpetic stomatitis).mp.) **AND**(exp Europe/ or exp Cyprus/ or exp Greenland/ or exp Israel/ or exp USSR or exp Turkey republic/ or (Albania* or Andorra* or Armenia* or Austria* or Azerbaijan* or Belarus* or Belgi* or Bosnia* or Bosnia and Herzegovina or Bulgaria* or Croatia* or Cypr* or Czech Republic* or Czech* or Denmark* or Danish* or Estonia* or Finland* or Finnish* or France* or French* or Georgia* or German* or Gree* or Greenlan* or Hungar* or Iceland* or Ireland* or Irish* or Israel* or Ital* or Kazakh* or Kyrgyz* or Latvia* or Lithuania* or Luxembourg* or Malta* or Monac* or Montenegr* or Netherlands* or Dutch* or Norway* or Norweg* or Poland* or Polish* or Portug* or Republic of Moldova* or Moldov* or Romania* or Russia* or Russian Federation* or San Marino* or Serbia* or Slovakia* or Slovenia* or Spain* or Spanish* or Swed* or Switzerland* or Swiss* or Tajik* or Yugoslav Republic of Macedonia* or Macedonia* or Turk* or Turkmen* or Ukrain* or United Kingdom* or Great Britain* or UK or Uzbek*).mp.) |

Abbreviations: HSV-2 = Herpes simplex virus type 2.

**Box S2. Extracted variables included in the study.**

| 1. Author(s) 2. Publication title 3. Year of publication 4. Year(s) of data collection 5. Subregion 6. Country of origin 7. Country of survey 8. City 9. Study site 10. Study design 11. Study sampling procedure 12. Study population and its characteristics (e.g., sex and age) 13. Sample size 14. Response rate 15. HSV-2 outcome measures 16. Diagnostic assay |
| --- |

**Table S3. Data sources and search criteria for reviewing neonatal herpes epidemiology in Europe.**

| **PubMed (last searched August 16, 2022):** |
| --- |
| (Neonatal herpes[MeSH] OR Neonatal herpes[Text]) **AND** (“Europe”[MeSH] OR Greenland[MeSH] “Israel”[MeSH] OR “Turkey”[MeSH] OR “USSR”[MeSH] OR “Cyprus”[MeSH] OR Albania*[Text] OR Andorra*[Text] OR Armenia*[Text] OR Austria*[Text] OR Azerbaijan*[Text] OR Belarus*[Text] OR Belgi*[Text] OR Bosnia*[Text] OR Bosnia and Herzegovina[Text] OR Bulgaria*[Text] OR Croatia*[Text] OR Cypr*[Text] OR Czech Republic[Text] OR Czech*[Text] OR Denmark*[Text] OR Danish[Text] OR Estonia*[Text] OR Finland*[Text] OR Finnish[Text] OR France*[Text] OR French[Text] OR Georgia*[Text] OR German*[Text] OR Gree*[Text] OR Greenlan*[Text] OR Hungar*[Text] OR Iceland*[Text] OR Ireland*[Text] OR Irish[Text] OR Israel*[Text] OR Ital*[Text] OR Kazakh*[Text] OR Kyrgyz*[Text] OR Latvia*[Text] OR Lithuania*[Text] OR Luxembourg*[Text] OR Malta*[Text] OR Monac*[Text] OR Montenegr*[Text] OR Netherlands*[Text] OR Dutch[Text] OR Norway*[Text] OR Norweg*[Text] OR Poland*[Text] OR Polish[Text] OR Portug*[Text] OR Republic of Moldova*[Text] OR Moldov*[Text] OR Romania*[Text] OR Russia*[Text] OR Russian Federation*[Text] OR San Marino*[Text] OR Serbia*[Text] OR Slovakia*[Text] OR Slovenia*[Text] OR Spain*[Text] OR Spanish[Text] OR Swed*[Text] OR Switzerland*[Text] OR Swiss[Text] OR Tajik*[Text] OR Yugoslav*[Text] OR Republic of Macedonia*[Text] OR Macedonia*[Text] OR Turkey*[Text] OR Turkmen*[Text] OR Ukrain*[Text] OR United Kingdom*[Text] OR Great Britain*[Text] OR UK[Text] OR Uzbek*[Text]) |
| **Embase (last searched August 16, 2022):** |
| (exp neonatal herpes) OR (neonatal herpes.mp.) **AND**(exp Europe/ or exp Cyprus/ or exp Greenland/ or exp Israel/ or exp USSR or exp Turkey republic/ or (Albania* or Andorra* or Armenia* or Austria* or Azerbaijan* or Belarus* or Belgi* or Bosnia* or Bosnia and Herzegovina or Bulgaria* or Croatia* or Cypr* or Czech Republic* or Czech* or Denmark* or Danish* or Estonia* or Finland* or Finnish* or France* or French* or Georgia* or German* or Gree* or Greenlan* or Hungar* or Iceland* or Ireland* or Irish* or Israel* or Ital* or Kazakh* or Kyrgyz* or Latvia* or Lithuania* or Luxembourg* or Malta* or Monac* or Montenegr* or Netherlands* or Dutch* or Norway* or Norweg* or Poland* or Polish* or Portug* or Republic of Moldova* or Moldov* or Romania* or Russia* or Russian Federation* or San Marino* or Serbia* or Slovakia* or Slovenia* or Spain* or Spanish* or Swed* or Switzerland* or Swiss* or Tajik* or Yugoslav Republic of Macedonia* or Macedonia* or Turk* or Turkmen* or Ukrain* or United Kingdom* or Great Britain* or UK or Uzbek*).mp.) |

**Table S4. Studies reporting HSV-2 incidence in Europe among different at-risk populations.**

| **Name** | **Year(s) of data collection** | **Country** | **Study design** | **Population characteristics** | **HSV-2 biological assay** | **Sample size** | **Follow-up duration** | **HSV-2 seroconversion rate (%)** | **HSV-2 incidence rate**  **(per 100 person years)** |
| --- | --- | --- | --- | --- | --- | --- | --- | --- | --- |
| **General populations** |  |  |  |  |  |  |  |  |  |
| Arvaja, 1999^2^ | 1988-89 | Finland | Cohort | Pregnant women | ELISA | 840 | - | 0·60 | - |
| Eskild, 1999^3^ | 1994-96 | Norway | Cohort | Pregnant women | EIA | 623 | 26 weeks | 2·60 | - |
| Suligoi, 2004^4^ | 1998-99 | Italy | Cohort | Healthy adolescents | ELISA | 336 | 6 years | - | 0·40 |
| **Intermediate-risk populations** | | |  |  |  |  |  |  |  |
| Davidovici, 2006^5^ | 1984-85 | Israel | Cohort | Young soldiers | ELISA | 421 | 2-3 years | 3·09 | 1·30 |
| Davidovici, 2006^5^ | 1992-93 | Israel | Cohort | Young soldiers | ELISA | 402 | 2-3 years | 1·74 | 0·70 |
| Davidovici, 2006^5^ | 2001-02 | Israel | Cohort | Young soldiers | ELISA | 335 | 2-3 years | 0·30 | 0·12 |
| **Higher-risk populations** |  |  |  |  |  |  |  |  |  |
| Keet, 1990^6^ | 1984 | Netherlands | Cohort | HIV negative MSM | ELISA | 36 | - | 8·00 | - |
| **STI clinic attendees and symptomatic populations** | | | |  |  |  |  |  |  |
| Varela, 2001^7^ | 1996-97 | Spain | Cohort | STI clinic attendees | WB | 145 | 6-18 months | 1·38 | - |
| **People living with HIV and people in HIV discordant couples** | | | |  |  |  |  |  |  |
| Keet, 1990^6^ | 1984 | Netherlands | Cohort | MSM living with HIV infection | ELISA | 18 | - | 17·00 | - |

Abbreviations: EIA = Enzyme immunoassay, ELISA = Enzyme-linked immunosorbent type-specific assay, HIV = Human immunodeficiency virus, HSV-2 = Herpes simplex virus type 2, MAb = Monoclonal antibody, MSM = Men who have sex with men, STI = Sexually transmitted infection, UK = United Kingdom of Great Britain and Northern Ireland, WB = Western blot.

**Table S5. Studies reporting HSV-2 seroprevalence in Europe among general populations.**

| **Author, year** | **Year(s) of data collection** | **Country** | **Study site** | **Study design** | **Sampling** | **Population** | **HSV-2 serological assay** | **Sample size** | | **HSV-2 seroprevalence (%)** |
| --- | --- | --- | --- | --- | --- | --- | --- | --- | --- | --- |
| **General populations** |  |  |  |  |  |  |  |  | |  |
| Ades, 1989^8^ | 1980-81 | UK | OC | CS | Conv | Pregnant women | ELISA | 3,533 | | 10·4 |
| Alanen, 2005^9^ | 2000 | Finland | Hospital | CS | Conv | Pregnant women | ELISA | 558 | | 9·3 |
| Andersson-Ellstrom, 1995^10^ | 1989-90 | Sweden | Community | CS | Conv | 15-17 years old women | ELISA | 98 | | 1·0 |
| Arama, 2010^11^ | 2004-05 | Romania | Hospital | CS | Conv | General population | ELISA | 1,070 | | 15·2 |
| Arnheim, 2011^12^ | 1975-02 | Mixed countries^a^ | Community | CC | Conv | Healthy controls | ELISA | 2,968 | | 14·0 |
| Arvaja, 1999^2^ | 1988-89 | Finland | OC | CS | RS | Pregnant women | ELISA | 997 | | 15·8 |
| Balaeva, 2016^13^ | 2010-11 | Russian Fed | Community | CS | Conv | Young adult population in Arkhangelsk | ELISA | 1,243 | | 18·8 |
| Benharrosh, 2008^14^ | - | France | OC | CS | Conv | Pregnant women | ELISA | 307 | | 15·3 |
| Berntsson, 2009^15^ | 2002 | Sweden | OC | CS | RS | Pregnant women | ELISA | 299 | | 9·0 |
| Bjerke, 2010^16^ | 2007-09 | Norway | OC | CS | Conv | Pakistani women living in Norway | ELISA | 112 | | 4·0 |
| Bjerke, 2010^16^ | 2007-09 | Norway | OC | CS | Conv | Husbands of Pakistani women | ELISA | 112 | | 2·0 |
| Blomstrom, 2012^17^ | - | Sweden | Community | CC | Conv | Healthy controls | ELISA | 524 | | 24·2 |
| Bodeus, 2004^18^ | 2001-02 | Belgium | Hospital | CS | Conv | Pregnant women | ELISA | 1,000 | | 18·2 |
| Bunzli, 2004^19^ | 1992-93 | Switzerland | Community | CS | RS | Adult populations in Vaud-Fribourg and Ticino | ELISA | 3,120 | | 18·6 |
| Bystricka, 1998^20^ | - | Slovakia | Community | CS | RS | HIV negative blood donors | WB | 37 | | 11·0 |
| Canessa, 1987^21^ | 1982-84 | Italy | OC | CS | Conv | Pregnant women | ELISA | 804 | | 69·0 |
| Celentano, 2010^22^ | - | Russian Fed | Community | RCT^b^ | Conv | Males | EIA | 1,136 | | 1·4 |
| Celentano, 2010^22^ | - | Russian Fed | Community | RCT^b^ | Conv | Females | EIA | 1,068 | | 4·0 |
| Cheslack-Postava, 2015^23^ | 1983-98 | Finland | Community | CC | Conv | Mothers of Schizophrenia patients | ELISA | 963 | | 16·4 |
| Cheslack-Postava, 2015^23^ | 2009 | Finland | Community | CC | RS | Mothers of controls | ELISA | 963 | | 12·6 |
| Christenson, 1992^24^ | 1972 | Sweden | Community | CS | Conv | 14–15 years old schoolgirls | ELISA | 739 | | 0·4 |
| Cliff, 2019^25^ | - | UK | Community | CC | Conv | Healthy controls | ELISA | 107 | | 33·9 |
| Cowan, 2003^26^ | 1998-00 | Estonia | OC | CS | Conv | Children | ELISA | 1,016 | | 0·5 |
| Cowan, 2003^26^ | 1998-00 | Estonia | OC | CS | Conv | Antenatal clinic attendees | ELISA | 794 | | 23·8 |
| Cowan, 2003^26^ | 1998-00 | Estonia | OC | CS | Conv | Male blood donors | ELISA | 574 | | 10·8 |
| Cowan, 2003^26^ | 1998-00 | Estonia | OC | CS | Conv | Female blood donors | ELISA | 462 | | 21·1 |
| Dan, 2003^27^ | 2000-01 | Israel | Hospital | CS | Conv | Pregnant women at delivery | ELISA | 512 | | 13·3 |
| Davidovici, 2006^28^ | 2000-01 | Israel | Community | CS | SRS | General population | ELISA | 3,677 | | 6·5 |
| De Ory, 1999^29^ | 1993-94 | Spain | Community | CS | RCS | Women of childbearing age | EIA | 692 | | 3·5 |
| De Ory, 2000^30^ | - | Spain | Community | CS | Conv | Adult males in the community | EIA | 542 | | 1·3 |
| De Sanjose, 1994^31^ | 1985-87 | Spain | Community | CC | SRS | Controls to women with invasive cervical cancer | ELISA | 238 | | 11·9 |
| De Sanjose, 1994^31^ | 1985-87 | Spain | Hospital | CC | Conv | Controls to women with CIN III | ELISA | 242 | | 10·6 |
| Dolar, 2006^32^ | - | Turkey | Community | CS | Conv | Sexually active healthy adults | ELISA | 725 | | 4·8 |
| Dolar, 2006^32^ | - | Turkey | Community | CS | Conv | Pregnant women in Istanbul | ELISA | 300 | | 5·0 |
| Dolar, 2006^32^ | - | Turkey | Community | CS | Conv | Blood donors in Istanbul | ELISA | 200 | | 5·5 |
| Dordević, 2006^33^ | 2005 | Serbia | OC | CS | Conv | Pregnant women | ELISA | 32 | | 12·5 |
| Dordević, 2006^33^ | 2005 | Serbia | OC | CS | Conv | Women of reproductive age | ELISA | 49 | | 12·2 |
| Eis-Hübinger, 1999^34^ | - | Germany | Hospital | CS | RS | Children hospital attendees | EIA | 121 | | 0·0 |
| Eis-Hübinger, 1999^34^ | - | Germany | Community | CS | Conv | Healthy medical staff | EIA | 26 | | 0·0 |
| Eis-Hübinger, 1999^34^ | - | Germany | Hospital | CS | Conv | Healthy adults | EIA | 40 | | 10·0 |
| Eis-Hübinger, 1999^34^ | - | Germany | OC | CS | RS | Pregnant women | EIA | 205 | | 7·8 |
| Enders, 1998^35^ | 1988-89 | Germany | Community | CS | Conv | Pregnant women | EIA | 408 | | 8·3 |
| Enders, 1998^35^ | 1990-91 | Germany | Community | CS | Conv | Pregnant women | EIA | 592 | | 6·3 |
| Enders, 1998^35^ | 1996-97 | Germany | Community | CS | Conv | Pregnant women | EIA | 1,999 | | 8·9 |
| Enders, 1998^35^ | 1996-97 | Germany | Community | CS | Conv | 6-9 years old children | EIA | 31 | | 0·0 |
| Enders, 1998^35^ | 1996-97 | Germany | Community | CS | Conv | 15-40 years old men | EIA | 68 | | 8·8 |
| Enders, 1998^35^ | 1996-97 | Germany | Community | CS | Conv | 15-40 years old women | EIA | 797 | | 10·0 |
| Esteban-Hernández, 2011^36^ | 2000 | Spain | OC | CC | Conv | Patients with cardiovascular disease | ELISA | 75 | | 5·3 |
| Esteban-Hernández, 2011^36^ | 2000 | Spain | OC | CC | Conv | Healthy controls | ELISA | 75 | | 0·0 |
| Eskild et al, 2000^37^ | 1992-94 | Norway | OC | CS | RS | Pregnant women | ELISA | 960 | | 27·0 |
| Eskild et al, 2002^38^ | 1992-94 | Norway | OC | CC | Conv | First trimester pregnant women with a fetal death | ELISA | 281 | | 29·0 |
| Espinola-Klein, 2002^39^ | 1996-98 | Germany | Hospital | CS | RS | Patients with progression of atherosclerosis | ELISA | 116 | | 17·2 |
| Espinola-Klein, 2002^39^ | 1996-98 | Germany | Hospital | CS | RS | Patients without progression of atherosclerosis | ELISA | 311 | | 11·9 |
| Forbes, 2019^40^ | 1998-00 | UK | Community | CS | RS | Community dwelling aging population | ELISA | 9,929 | | 3·6 |
| Forsgren, 1994^41^ | 1969 | Sweden | OC | CS | Conv | Pregnant women | ELISA | 941 | | 17·0 |
| Forsgren, 1994^41^ | 1983 | Sweden | OC | CS | Conv | Pregnant women | ELISA | 1,759 | | 32·0 |
| Forsgren, 1994^41^ | 1989 | Sweden | OC | CS | Conv | Pregnant women | ELISA | 1,000 | | 33·0 |
| Garcia-Corbeira, 1999^42^ | - | Spain | Community | CS | Conv | Spanish university students | ELISA | 306 | | 1·3 |
| Garcia-Corbeira, 1999^43^ | 1992-93 | Spain | Community | CS | RS | Spanish general population | EIA | 3,974 | | 3·6 |
| Gaytant, 2002^44^ | 1998 | Netherlands | Community | CS | Conv | Pregnant women in the Netherlands | ELISA | 1,507 | | 24·4 |
| Gorander, 2008^45^ | 2005-06 | Poland | OC | CS | Conv | Blood donors | ELISA | 199 | | 5·0 |
| Hamdani, 2017^46^ | 2008-14 | France | OC | CC | Conv | French healthy controls | EIA | 180 | | 28·3 |
| Hawkes, 2006^47^ | 1995-02 | UK | Community | CS | Conv | Patients with multiple sclerosis | ELISA | 497 | | 13·5 |
| Hawkes, 2006^47^ | 1992 | UK | Community | CS | Conv | Blood donors | ELISA | 1,378 | | 8·0 |
| Hawkes, 2006^47^ | 2000 | UK | Community | CS | Conv | General population in London | ELISA | 3,646 | | 8·5 |
| Hellenbrand, 2005^48^ | 2005 | Germany | Community | CS | RS | General population in 1997-98 | ELISA | 3792 | | 13·3 |
| Hettmann, 2008^49^ | 1999 | Hungary | Community | CS | Conv | General population | ELISA | 2,500 | | 4·2 |
| Hettmann, 2008^49^ | 1999 | Hungary | Community | CS | Conv | Pregnant women | ELISA | 512 | | 2·6 |
| Isacsohn, 2002^50^ | 1998-99 | Israel | Community | CS | Conv | Children and adolescents | WB | 172 | | 0·0 |
| Isacsohn, 2002^50^ | 1998-99 | Israel | Community | CS | Conv | Healthy adults | WB | 716 | | 4·5 |
| Isacsohn, 2002^50^ | 1998-99 | Israel | Community | CS | Conv | Women in 1990-99 | WB | 180 | | 7·7 |
| Isacsohn, 2002^50^ | 1998-99 | Israel | Community | CS | Conv | Parturient women | WB | 155 | | 5·8 |
| Jha, 1993^51^ | 1990 | UK | Community | CC | Conv | Healthy controls | ELISA | 387 | | 4·7 |
| Jonsson et al, 1995^52^ | 1989 | Sweden | Community | CS | Conv | Healthy women | ELISA | 584 | | 6·0 |
| Jonsson, 2006^53^ | 1990-91 | Sweden | Community | CS | Conv | Swedish general population | ELISA | 2,399 | | 13·0 |
| Jonsson, 2006^53^ | 1996-97 | Sweden | Community | CS | Conv | Swedish general population | ELISA | 500 | | 16·4 |
| Juhl, 2010^54^ | - | Germany | Hospital | CS | Conv | 18-67 years old blood donors | WB | 653 | | 7·4 |
| Karachaliou, 2016^55^ | 2007-08 | Greece | Community | CS | Conv | 4 years old children | ELISA | 674 | | 1·5 |
| Karachaliou, 2016^56^ | 2007-08 | Greece | Hospital | CS | Conv | 4 years old children | ELISA | 690 | | 1·4 |
| Karaer, 2013^57^ | 2003-05 | Turkey | Hospital | CC | Conv | Pregnant women | ELISA | 125 | | 4·0 |
| Khryanin, 2007^58^ | - | Russian Fed | Community | CS | RS | General population | ELISA | 443 | | 20·3 |
| Kibur, 2000^59^ | 1996-97 | Estonia | OC | Cohort^b^ | RS | Primiparous women | ELISA | 1,110 | | 12·7 |
| Korodi, 2005^60^ | 1968-72 | Finland | Community | CC | Conv | Finish matched controls | ELISA | 288 | | 6·9 |
| Korr, 2017^61^ | 2008-11 | Germany | Community | CS | Conv | Heathy population | ELISA | 5,013 | | 9·4 |
| Kramer, 2008^62^ | 2004 | Netherlands | Community | CS | SRS | General ethnic adult population | ELISA | 1,325 | | 22·0 |
| Krone, 2008^63^ | 1989-06 | Germany | OC | CC | Conv | Healthy children | ELISA | 152 | | 1·3 |
| Kucera, 2012^64^ | 2004-07 | Switzerland | OC | CS | Conv | First trimester pregnant women | ELISA | 1,030 | | 21·2 |
| Laubereau, 2000^65^ | 1997 | Switzerland | Community | CS | Conv | Male population | ELISA | 62 | | 8·1 |
| Laubereau, 2000^65^ | 1997 | Switzerland | Community | CS | Conv | Female population | ELISA | 89 | | 14·6 |
| LeGoff, 2007^66^ | 2004 | France | OC | CS | Conv | Healthy women | ELISA | 76 | | 26·3 |
| Lehtinen, 1996^67^ | 1968-72 | Finland | Community | CC | Conv | Healthy women | ELISA | 143 | | 25·9 |
| Lorber, 2006^68^ | 2003 | Slovenia | Community | CS | Conv | Pregnant women | ELISA | 4,000 | | 8·4 |
| Lowhagen, 1990^69^ | 1987 | Sweden | Hospital | CS | Conv | Pregnant women | ELISA | | 273 | 24·0 |
| Lowhagen, 1990^69^ | 1989 | Sweden | Hospital | CS | Conv | Pregnant women | ELISA | | 295 | 24·0 |
| Mahic, 2017^70^ | 1999-08 | Norway | OC | CC | Conv | Pregnant women | ELISA | | 903 | 13·2 |
| Malkin, 2002^71^ | 1996 | France | Community | CS | RS | General population | WB | | 4,412 | 17·2 |
| Maral, 2009^72^ | - | Turkey | Community | CS | Conv | Adult women | ELISA | | 1,115 | 53·5 |
| Marchesi, 2007^73^ | - | Italy | Community | CS | Conv | Black Italian residents | ELISA | | 22 | 95·0 |
| Marchesi, 2007^73^ | - | Italy | Community | CS | Conv | White Italian residents | ELISA | | 20 | 45·0 |
| Marchi, 2017^74^ | 2000 | Italy | Community | CS | Conv | Population of Siena | ELISA | | 621 | 22·4 |
| Marchi, 2017^74^ | 2005 | Italy | Community | CS | Conv | Population of Siena | ELISA | | 615 | 13·0 |
| Marchi, 2017^74^ | 2013-14 | Italy | Community | CS | Conv | Population of Siena | ELISA | | 497 | 11·5 |
| Marchi, 2017^74^ | 2005 | Italy | Community | CS | Conv | Population of Bari | ELISA | | 168 | 4·8 |
| Marchi, 2017^74^ | 2003-05 | Italy | Community | CS | Conv | Pregnant women in Bari | ELISA | | 91 | 9·9 |
| Miskulin, 2011^75^ | 2005 | Croatia | Community | CS | RS | Blood donors | WB | | 423 | 3·3 |
| Morris-Cunnington, 2004^76^ | 1991 | UK | Community | CS | Conv | General population of England | ELISA | | 2,259 | 11·6 |
| Munoz, 1995^77^ | 1985-87 | Spain | Hospital | CC | RS | Women healthy controls | ELISA | | 189 | 36·0 |
| Munoz, 1995^77^ | 1985-87 | Spain | OC | CC | Conv | Healthy controls | ELISA | | 213 | 27·2 |
| Munoz, 1995^77^ | 1985-87 | Spain | Community | CC | Conv | Husbands of healthy controls | ELISA | | 120 | 40·8 |
| Munoz, 1995^77^ | 1985-87 | Spain | Community | CC | Conv | Husbands of healthy controls | ELISA | | 169 | 30·8 |
| Nilsen, 2003^78^ | - | Norway | OC | CS | Conv | Healthy adults | ELISA | 68 | | 8·8 |
| Nilsen, 2005^79^ | - | Norway | Community | CS | Conv | Pregnant women | ELISA | 110 | | 13·6 |
| Nilsen, 2005^79^ | - | Norway | Community | CS | Conv | Blood donors | ELISA | 100 | | 7·0 |
| Nilsen, 2005^79^ | - | Norway | Community | CS | Conv | Medical students | ELISA | 99 | | 4·0 |
| NIMH group, 2007^80^ | 2001 | Russian Fed | Community | RCT^b^ | RS | Males living in school dormitories | ELISA | 470 | | 3·2 |
| NIMH group, 2007^80^ | 2001 | Russian Fed | Community | RCT^b^ | RS | Females living in school dormitories | ELISA | 471 | | 9·1 |
| Ohana, 2000^81^ | - | Israel | OC | CS | Conv | Samples tested using Gull commercial kit | ELISA | 233 | | 33·1 |
| Ohana, 2000^81^ | - | Israel | OC | CS | Conv | Samples tested using Sorin commercial kit | ELISA | 99 | | 7·1 |
| Ohana, 2000^81^ | - | Israel | OC | CS | Conv | Sample tested using MRL commercial kit | WB | 66 | | 24·2 |
| Ohana, 2000^81^ | - | Israel | OC | CS | Conv | Sample tested using Biokit commercial kit | ELISA | 81 | | 7·4 |
| Olsson, 2017^82^ | 2003-05 | Sweden | Community | CS | Conv | Adult Swedish population | ELISA | 535 | | 12·9 |
| Opaneye, 2002^83^ | 1996-97 | UK | OC | CC | Conv | Pregnant women as control | EIA | 198 | | 8·1 |
| Ozdemir, 2009^84^ | - | Turkey | OC | CS | Conv | Pregnant women | ELISA | 158 | | 8·2 |
| Papadogeorgakis, 2008^85^ | 2004-05 | Greece | OC | CS | Conv | General population | ELISA | 1,867 | | 10·2 |
| Pasquini, 1988^86^ | 1981 | Italy | Community | CS | Conv | Healthy young males/recruits | ELISA | 1,169 | | 0·1 |
| Pasquini, 1988^86^ | 1985 | Italy | Community | CS | Conv | Health care workers | ELISA | 411 | | 4·8 |
| Pebody, 2004^87^ | 1999-00 | Belgium | Community | CS | RS | General population | ELISA | 2,983 | | 10·7 |
| Pebody, 2004^87^ | 1999 | Bulgaria | Community | CS | Conv | General population | ELISA | 2,750 | | 23·2 |
| Pebody, 2004^87^ | 1989 | Czech Republic | Community | CS | RS | General population | ELISA | 3,098 | | 4·7 |
| Pebody, 2004^87^ | 1997-98 | Finland | Community | CS | Conv | General population | ELISA | 2,567 | | 12·1 |
| Pebody, 2004^87^ | 1996 | Netherlands | Community | CS | MSCS | General population | ELISA | 5,954 | | 9·0 |
| Pebody, 2004^87^ | 1993 | Slovenia | Community | CS | Conv | Female population | ELISA | 3,000 | | 7·2 |
| Persson, 1995^88^ | 1970-73 | Sweden | Hospital | CS | Conv | Pregnant women | ELISA | 1,198 | | 21·0 |
| Persson, 1995^88^ | 1979 | Sweden | Hospital | CS | Conv | Pregnant women | ELISA | 294 | | 21·0 |
| Persson, 1995^88^ | 1990-93 | Sweden | Hospital | CS | Conv | Pregnant women in 1990, 1991 and 1993 | ELISA | 1,190 | | 21·0 |
| Petersen, 2000^89^ | - | Denmark | Community | CS | Conv | >18 years old men | ELISA | 94 | | 15·0 |
| Petersen, 2000^89^ | - | Denmark | Community | CS | Conv | >19 years old women | ELISA | 49 | | 26·0 |
| Puhakka, 2016^90^ | 1992 | Finland | Community | CS | RS | Pregnant women | ELISA | 200 | | 17·5 |
| Puhakka, 2016^90^ | 2002 | Finland | Community | CS | RS | Pregnant women | ELISA | 200 | | 15·0 |
| Puhakka, 2016^90^ | 2012 | Finland | Community | CS | RS | Pregnant women | ELISA | 200 | | 11·0 |
| Rabenau, 2002^91^ | 1997-01 | Germany | Hospital | CS | Conv | Female controls | ELISA | 2,473 | | 17·0 |
| Rabenau, 2002^91^ | 1997-01 | Germany | Hospital | CS | Conv | Male controls | ELISA | 1,879 | | 12·5 |
| Rabenau, 2002^91^ | 1997-01 | Germany | Hospital | CS | Conv | Male OTX recipients | ELISA | 51 | | 9·6 |
| Rabenau, 2002^91^ | 1997-01 | Germany | Hospital | CS | Conv | Female OTX recipients | ELISA | 31 | | 21·9 |
| Rode, 2008^92^ | 1999-01 | Croatia | Hospital | CS | Conv | Blood donors | ELISA | 219 | | 8·7 |
| Sauerbrei, 2011^93^ | 1999-06 | Germany | Community | CS | RS | Infants to adolescents | ELISA | 1,100 | | 4·2 |
| Sauerbrei, 2011^93^ | 1999-00 | Germany | Community | CS | RS | Adults blood donors | ELISA | 800 | | 13·6 |
| Sauerbrei, 2011^93^ | 1999-00 | Germany | Community | CS | Conv | 17-40 years old pregnant women | ELISA | 200 | | 18·0 |
| Shev, 1995^94^ | 1990-92 | Sweden | Community | CC | Conv | HCV negative blood donors | ELISA | 41 | | 17·0 |
| Silins, 2000^95^ | 1989-91 | Sweden | Community | CS | Conv | Women using contraceptives | ELISA | 266 | | 20·7 |
| Slomka, 1995^96^ | 1991 | UK | Hospital | CS | Conv | Children and young adolescents | MAb | 100 | | 0·0 |
| Slomka, 1995^96^ | 1988 | UK | Community | CS | Conv | Adult blood donors | MAb | 104 | | 2·9 |
| Smith, 2006^97^ | 2002 | Poland | Community | CS | SRS | Polish population | ELISA | 2,257 | | 9·1 |
| Smith, 2006^97^ | 2000 | Poland | Community | CS | Conv | Population in Mazowieckie | ELISA | 222 | | 11·3 |
| Snijders, 2019^98^ | 2011 | Netherlands | Hospital | CC | Conv | Healthy controls | ELISA | 132 | | 7·6 |
| Snijders, 2019^98^ | 2011 | Netherlands | Hospital | CC | Conv | Family of patients with bipolar disorder | ELISA | 144 | | 4·2 |
| Stock, 2001^99^ | - | Mixed countries^c^ | Community | CS | Conv | University students | ELISA | 352 | | 3·4 |
| Strandberg, 2003^100^ | - | Finland | Community | CS | RS | Elderly with cardiovascular disease | ELISA | 325 | | 31·4 |
| Strandberg, 2003^100^ | - | Finland | Community | CS | RS | Elderly patients | ELISA | 58 | | 51·7 |
| Suligoi, 2004^4^ | 1992-93 | Italy | Community | CS | Conv | 12 years old children | ELISA | 345 | | 2·6 |
| Suligoi, 2000^101^ | 1998 | Italy | Hospital | CS | Conv | Outpatients | ELISA | 272 | | 5·5 |
| Suligoi, 2000^101^ | 1998 | Italy | Hospital | CS | Conv | Blood donors | ELISA | 179 | | 6·1 |
| Suligoi, 2000^101^ | 1998 | Italy | Hospital | CS | Conv | Pregnant women | ELISA | 66 | | 7·6 |
| Suligoi, 2009^102^ | 2005 | Serbia | Hospital | CS | Conv | General population in Kosovo | EIA | 1,285 | | 20·2 |
| Topbas, 2012^103^ | 2007-08 | Turkey | Community | CS | Conv | 20-49 years old adults | ELISA | 1,983 | | 7·6 |
| Tunback, 2003^104^ | 1996-97 | Sweden | OC | CS | Conv | <19 years old children | WB | 2,106 | | 0·5 |
| Vass-Sorensen, 1984^105^ | 1980 | Norway | Hospital | CC | RS | Healthy controls to women with cervical cancer | WB | 30 | | 17·0 |
| Vilibić-Čavlek, 2017^106^ | 2013-15 | Croatia | OC | CC | Conv | Healthy controls | WB | 150 | | 12·7 |
| Vilibić-Čavlek, 2011^107^ | 2005-09 | Croatia | Hospital | CS | Conv | Women of childbearing age | EIA | 502 | | 6·8 |
| Vilibić-Čavlek, 2011^108^ | 2008-10 | Croatia | OC | CS | Conv | Croatian general population | WB | 1,672 | | 9·9 |
| Vyse, 2000^109^ | 1994-95 | UK | Community | CS | Conv | Male general population of England and Wales | ELISA | 1,674 | | 3·3 |
| Vyse, 2000^109^ | 1994-95 | UK | Community | CS | Conv | Female general population of England and Wales | ELISA | 1,673 | | 5·1 |
| Wang, 2011^110^ | 2001-07 | Netherlands | Community | Cohort^b^ | Conv | Dutch preadolescents in primary schools | EIA | 1,176 | | 0·9 |
| Warnecke, 2020^111^ | 2006-18 | Germany | OC | CS | Conv | Healthy women | WB | 202 | | 8·3 |
| Warnecke, 2020^111^ | 2018-19 | Poland | OC | CS | Conv | Heathy women | WB | 193 | | 4·1 |
| Warnecke, 2020^111^ | 2018 | Turkey | OC | CS | Conv | Healthy women | WB | 97 | | 2·1 |
| Werler, 2016^112^ | 1987-12 | Finland | Community | CC | RS | Mothers of children with gastroschisis | ELISA | 271 | | 38·0 |
| Werler, 2016^112^ | 1987-12 | Finland | Community | CC | RS | Mothers of control children | ELISA | 753 | | 34·9 |
| Woestenberg, 2016^113^ | 2006-07 | Netherlands | Community | CS | SRS | General population | ELISA | 3,757 | | 6·0 |
| Wutzler, 2000^114^ | 1996-97 | Germany | Community | CS | Conv | Population with low risk of STI | WB | 5,058 | | 12·8 |

^a^ This study was conducted in Finland, Iceland, Norway, and Sweden.

^b^ The reported study design is the original study design. The included measures in this study are those for the baseline measures at the beginning of the study.

^c^ This study was conducted in Germany and Spain.

Abbreviations: CC = Case-control, CIN = Cervical intraepithelial neoplasia, Conv = Convenience, CS = Cross-sectional, EIA = Enzyme immunoassay, ELISA = Enzyme-linked immunosorbent type-specific assay, HCV = Hepatitis C virus, HIV = Human immunodeficiency virus, HSV-2 = Herpes simplex virus type 2, MAb = Monoclonal antibody, MSCS = Multiple stage cluster sampling, OC = Outpatient clinic, OTX = Organ transplant, RCS = Random cluster sampling, RCT = Randomized controlled trial, RS = Random sampling, Russian Fed = Russian Federation, SRS = Stratified random sampling, STI = Sexually transmitted infection, UK = United Kingdom of Great Britain and Northern Ireland, WB = Western blot.

**Table S6. Studies reporting HSV-2 seroprevalence in Europe among different at-risk populations.**

| **Author, year** | **Year(s) of data collection** | **Country** | **Study site** | **Study design** | **Sampling** | **Population** | **HSV-2 serological assay** | **Sample size** | **HSV-2 seroprevalence (%)** |
| --- | --- | --- | --- | --- | --- | --- | --- | --- | --- |
| **Intermediate-risk populations** | |  |  |  |  |  |  |  |  |
| Chacowry, 2018^115^ | 2011 | Switzerland | Prison | CS | Conv | Male detainees in prison | ELISA | 156 | 22·4 |
| Christensen, 2002^116^ | - | Denmark | Prison | CS | Conv | Prisoners | EIA | 330 | 17·6 |
| Christensen, 2002^116^ | - | Denmark | OC | CS | Conv | Drug treatment user center | EIA | 137 | 32·9 |
| Davidovici, 2006^5^ | 1982 | Israel | - | CC | Conv | Young soldiers recruited | ELISA | 445 | 8·4 |
| Davidovici, 2006^5^ | 1990 | Israel | - | CC | Conv | Young soldiers recruited | ELISA | 416 | 5·1 |
| Davidovici, 2006^5^ | 1999 | Israel | - | CC | Conv | Young soldiers recruited | ELISA | 340 | 2·0 |
| Dolar, 2006^32^ | - | Turkey | Community | CS | Conv | Hotel staff in Istanbul | ELISA | 264 | 8·3 |
| Sarmati, 2007^117^ | 2001-02 | Italy | Prison | CS | Conv | Inmates | ELISA | 876 | 21·2 |
| Suligoi, 2000^101^ | 1998 | Italy | Hospital | CS | Conv | Military recruits | ELISA | 156 | 3·8 |
| **Higher-risk populations** | |  |  |  |  |  |  |  |  |
| Bozicevic, 2009^118^ | 2006 | Croatia | Hospital | CS | RDS | MSM | WB | 360 | 9·4 |
| Bozicevic, 2012^119^ | 2010-11 | Croatia | Hospital | CS | RDS | MSM | WB | 387 | 5·9 |
| Bystricka, 2000^120^ | - | Slovakia | Community | CS | Conv | HIV negative MSM | WB | 52 | 23·1 |
| Bystricka, 2003^121^ | - | Slovakia | Community | CS | Conv | FSWs | WB | 18 | 50·0 |
| Dolar, 2006^32^ | - | Turkey | Community | CS | Conv | FSWs in Istanbul | ELISA | 483 | 60·0 |
| Eing, 2002^122^ | - | Germany | OC | CS | Conv | FSWs | WB | 194 | 46·4 |
| Fox, 2006^123^ | - | UK | Community | CS | Conv | FSWs | ELISA | 453 | 60·0 |
| Hill, 2009^124^ | 2003 | UK | OC | CS | SRS | MSM population | MAb | 3,968 | 22·7 |
| Linhart, 2008^125^ | - | Israel | Community | CS | Conv | FSWs | ELISA | 300 | 60·0 |
| Papadogeorgaki, 2006^126^ | 2005 | Greece | Hospital | CS | Conv | FSWs | EIA | 299 | 69·6 |
| Pasquini, 1988^86^ | 1984 | Italy | Community | Cohort^a^ | Conv | MSM | ELISA | 397 | 55·0 |
| Rabenau, 2002 ^91^ | 1997-01 | Germany | Community | CS | Conv | FSWs | ELISA | 300 | 80·0 |
| Smit, 2007^127^ | 1984-03 | Netherlands | Community | Cohort^a^ | Conv | MSM | ELISA | 1,847 | 41·0 |
| **STI clinic attendees and symptomatic populations** | | |  |  |  |  |  |  |  |
| Al-Sulaiman, 2009^128^ | 2006-07 | UK | OC | CS | Conv | Samples collected from serum bank | ELISA | 218 | 24·3 |
| Bamberger, 2005^129^ | 1999-00 | Israel | OC | CS | Conv | Patients with hematospermia | ELISA | 16 | 25·0 |
| Berntsson, 2009^15^ | 2000-01 | Sweden | OC | CS | Conv | STI clinic attendees | ELISA | 290 | 22·8 |
| Cowan, 1994^130^ | 1990-91 | UK | OC | CS | Conv | GUM clinic attendees | WB | 833 | 22·7 |
| Cusini, 2000^131^ | 1997-98 | Italy | OC | CS | Conv | STI clinic attendees | ELISA | 919 | 24·6 |
| De Ory, 2018^132^ | - | Spain | OC | CS | Conv | Samples collected from a laboratory | ELISA | 384 | 27·6 |
| Dolar, 2006^32^ | - | Turkey | OC | CS | Conv | Patients with genital warts | ELISA | 110 | 17·3 |
| Enbom, 2001^133^ | - | Sweden | OC | CS | Conv | STI clinic attendees | ELISA | 112 | 12·0 |
| Evans, 2003^134^ | 1992 | UK | OC | CS | Conv | Female STI clinic attendees | MAb | 520 | 26·0 |
| Feldman, 2003^135^ | 1998-99 | Israel | OC | CS | Conv | GUM clinic attendees | EIA | 472 | 10·6 |
| Glinsek Biskup, 2015^136^ | 2006-08 | Slovenia | OC | CS | Conv | STI clinic attendees | ELISA | 227 | 29·5 |
| Glinsek Biskup, 2015^136^ | 2009-14 | Slovenia | OC | CS | Conv | STI clinic attendees | ELISA | 1,767 | 16·0 |
| Gorander, 2008^45^ | 2005-06 | Poland | OC | CS | Conv | STI clinic attendees | ELISA | 110 | 14·0 |
| Groen, 1998^137^ | 1993-94 | Netherlands | OC | CS | Conv | STI clinic attendees | WB | 1,250 | 27·0 |
| Janier, 2002^138^ | 1994 | France | OC | CS | Conv | Patients attending STI clinic | EIA | 512 | 43·0 |
| Janier, 2006^139^ | 1999-00 | France | OC | CS | Conv | Patients with genital lesions | ELISA | 239 | 77·0 |
| Joffe, 2006^140^ | 2000-01 | Israel | OC | CC | Conv | STI clinic attendees | ELISA | 175 | 68·8 |
| Lowhagen, 1990^69^ | 1987-88 | Sweden | OC | CS | Conv | Male STI clinic attendees | ELISA | 1,143 | 14·0 |
| Lowhagen, 1990^69^ | 1987-88 | Sweden | OC | CS | Conv | Female STI clinic attendees | ELISA | 475 | 26·0 |
| Lowhagen, 2000^141^ | 1995-99 | Sweden | OC | CS | Conv | Partners of HSV-1/HSV-2 infected patients | ELISA | 29 | 37·9 |
| Lowhagen, 2001^142^ | 1996-99 | Sweden | OC | CS | Conv | Partners of HSV-2 positive patients | ELISA | 26 | 58·0 |
| Lowhagen, 2005^143^ | 2000-01 | Sweden | OC | CS | Conv | STI clinic attendees | ELISA | 1,014 | 15·0 |
| Mele, 1988^144^ | 1986 | Italy | OC | CS | Conv | Italian STI outpatients | ELISA | 941 | 41·0 |
| Mullan, 2003^145^ | - | UK | OC | CS | Conv | STI clinic attendees | ELISA | 102 | 19·6 |
| Narouz, 2003^146^ | - | UK | OC | CS | Conv | Patients with genital herpes | EIA | 216 | 19·9 |
| Nilsen, 2005^79^ | - | Norway | OC | CS | Conv | Norwegian STI clinic attendees | ELISA | 601 | 16·8 |
| Opaneye, 2002^83^ | 1996-97 | UK | OC | CC | Conv | Female GUM clinic attendees | EIA | 269 | 21·6 |
| Papadogeorgaki, 2006^126^ | 2005 | Greece | Hospital | CS | Conv | Women with a clinical diagnosis of HSV-2 | EIA | 130 | 29·2 |
| Ramaswamy, 2005^147^ | - | UK | OC | CS | Conv | Patients with genital herpes | ELISA | 70 | 45·7 |
| Roest, 2001^148^ | 1998 | Netherlands | OC | CS | RS | STI clinic attendees | ELISA | 654 | 22·0 |
| Slomka, 1995^96^ | 1993 | UK | OC | CS | Conv | GUM clinic attendees | MAb | 80 | 25·0 |
| Smith, 1999^149^ | 1995-96 | UK | OC | CS | Conv | Patients with genital herpes | ELISA | 2,718 | 25·4 |
| Svennerholm, 1984^150^ | - | Sweden | OC | CS | Conv | Patients with herpes related symptoms | ELISA | 52 | 65·4 |
| van de Laar, 1998^151^ | 1986-88 | Netherlands | OC | CS | Conv | STI clinic attendees | ELISA | 1,679 | 32·3 |
| van de Laar, 1998^151^ | 1986-88 | Netherlands | OC | CS | Conv | Sexual partners to STI clinic attendees | ELISA | 119 | 30·3 |
| van Rooijen, 2016^152^ | 2000-11 | Netherlands | OC | Cohort^a^ | Conv | Patients with recurrent genital herpes | ELISA | 33 | 84·9 |
| varela, 2001^7^ | 1996-97 | Spain | OC | CS | Conv | STI clinic attendees | WB | 374 | 25·0 |
| Woolley, 2000^153^ | - | UK | OC | CS | Conv | STI clinic attendees | EIA | 488 | 14·3 |
| **People living with HIV and people in HIV discordant couples** | | | | |  |  |  |  |  |
| Allan, 2004^154^ | 2000-01 | UK | OC | CS | Conv | Patients living with HIV attending GUM clinic | EIA | 92 | 67·0 |
| Andreoletti, 2005^155^ | - | France | Hospital | CS | Conv | French adults infected living with HIV | ELISA | 534 | 59·0 |
| Bystricka, 1998^20^ | - | Slovakia | Community | CS | Conv | Patients living with HIV | WB | 30 | 37·0 |
| Bystricka, 2000^120^ | - | Slovakia | Community | CS | Conv | MSM living with HIV | WB | 27 | 40·7 |
| Joffe, 2006^140^ | 2000-01 | Israel | Community | CC | Conv | Patients living with HIV | ELISA | 200 | 9·0 |
| Lidon, 2019^156^ | 2008-12 | Spain | Hospital | CS | Conv | MSM living with HIV | ELISA | 141 | 54·0 |
| Pere, 2016^157^ | - | France | OC | Cohort^a^ | Conv | Women living with HIV | ELISA | 22 | 86·0 |
| Rabenau, 2002^91^ | 1997-01 | Germany | Hospital | CS | Conv | Males living with HIV | ELISA | 70 | 52·8 |
| Rabenau, 2002^91^ | 1997-01 | Germany | Hospital | CS | Conv | Females living with HIV | ELISA | 64 | 63·1 |
| Rode, 2008^92^ | 1999-01 | Croatia | Hospital | CS | Conv | Patients living with HIV | ELISA | 166 | 45·8 |
| Spielmann, 2010^158^ | 1996-99 | Germany | Community | Cohort^a^ | Conv | MSM living with HIV | ELISA | 132 | 44·7 |
| Spielmann, 2010^158^ | 2000 | Germany | Community | Cohort^a^ | Conv | MSM living with HIV | ELISA | 44 | 31·8 |
| Spielmann, 2010^158^ | 2001 | Germany | Community | Cohort^a^ | Conv | MSM living with HIV | ELISA | 62 | 33·3 |
| Spielmann, 2010^158^ | 2002 | Germany | Community | Cohort^a^ | Conv | MSM living with HIV | ELISA | 74 | 42·7 |
| Spielmann, 2010^158^ | 2003 | Germany | Community | Cohort^a^ | Conv | MSM living with HIV | ELISA | 130 | 42·0 |
| Spielmann, 2010^158^ | 2004 | Germany | Community | Cohort^a^ | Conv | MSM living with HIV | ELISA | 176 | 39·8 |
| Spielmann, 2010^158^ | 2005 | Germany | Community | Cohort^a^ | Conv | MSM living with HIV | ELISA | 170 | 39·4 |
| Spielmann, 2010^158^ | 2006 | Germany | Community | Cohort^a^ | Conv | MSM living with HIV | ELISA | 170 | 42·9 |
| Spielmann, 2010^158^ | 2007 | Germany | Community | Cohort^a^ | Conv | MSM living with HIV | ELISA | 94 | 38·3 |
| Sprenger, 2014^159^ | 2009-10 | Switzerland | OC | CS | Conv | Patients living with HIV | ELISA | 224 | 48·7 |
| Suligoi, 2002^160^ | 1983-95 | Italy | OC | CS | Conv | People living with HIV | ELISA | 380 | 33·2 |
| van Benthem, 2001^161^ | 1993 | Mixed countries^b^ | OC | CS | Conv | European women living with HIV | ELISA | 276 | 42·0 |
| Wutzler, 2000^114^ | 1997-98 | Germany | Community | CS | Conv | People living with HIV | WB | 382 | 47·9 |
| **Infertility clinic attendees and women with ectopic pregnancy** | | | |  |  |  |  |  |  |
| Ardizzoni, 2011^162^ | - | Italy | OC | CS | Conv | Women undergoing IVF | ELISA | 102 | 2·0 |
| Hettmann, 2008^49^ | 1999 | Hungary | OC | CS | Conv | Women attending infertility clinics | ELISA | 539 | 12·6 |
| Karaer, 2013^57^ | 2003-05 | Turkey | Hospital | CC | Conv | Women with ectopic pregnancy in Ankara | ELISA | 125 | 6·4 |
| **Other populations** |  |  |  |  |  |  |  |  |  |
| Arnheim, 2011^12^ | 1975-02 | Mixed countries^c^ | Community | CC | Conv | Women with invasive carcinoma | ELISA | 603 | 20·4 |
| Berglov, 2020^163^ | 2005-16 | Denmark | OC | CS | Conv | Women with chronic HBV | ELISA | 177 | 18·6 |
| Blomstrom, 2012^17^ | - | Sweden | Community | CC | Conv | Patients with psychosis | ELISA | 198 | 26·3 |
| Buxbaum, 2003^164^ | 1997-02 | Germany | Hospital | CS | Conv | Cases of suspected herpes encephalitis or meningitis | ELISA | 42 | 26·2 |
| Cliff, 2019^25^ | - | UK | Community | CC | Conv | Patients with severe fatigue syndrome | ELISA | 54 | 40·7 |
| Cliff, 2019^25^ | - | UK | Community | CC | Conv | Patients with mild-moderate fatigue syndrome | ELISA | 197 | 38·6 |
| Cliff, 2019^25^ | - | UK | Community | CC | Conv | Patients with multiple sclerosis | ELISA | 46 | 34·8 |
| De Sanjose, 1994^165^ | 1985-87 | Spain | Hospital | CC | Conv | Patients with invasive cervical cancer | ELISA | 223 | 25·8 |
| De Sanjose, 1994^165^ | 1985-87 | Spain | Hospital | CC | Conv | Patients with CIN grade 111 | ELISA | 249 | 14·3 |
| Gentile, 2014^166^ | 2010-13 | Italy | Hospital | CC | Conv | Patients with ASD | ELISA | 52 | 1·9 |
| Gentile, 2014^166^ | 2010-13 | Italy | Hospital | CC | Conv | Control patients without ASD | ELISA | 40 | 0·0 |
| Hamdani, 2017^46^ | 2008-14 | France | Hospital | CC | Conv | Patients with bipolar disorder | EIA | 138 | 23·9 |
| Hamdani, 2017^46^ | 2008-14 | France | Hospital | CC | Conv | Patients with schizophrenia | EIA | 105 | 15·2 |
| Jha, 1993^51^ | 1990 | UK | Community | CC | Conv | Women with invasive cervical cancer | ELISA | 219 | 11·4 |
| Korodi, 2005^60^ | 1968-72 | Finland | Community | CC | Conv | Patients with prostate cancer | ELISA | 163 | 6·8 |
| Krone, 2008^63^ | 1989-06 | Germany | OC | CC | Conv | Children with multiple sclerosis | ELISA | 152 | 2·0 |
| Lehtinen, 1996^167^ | 1968-72 | Finland | Community | CC | Conv | Women with cervical cancer | ELISA | 72 | 15·3 |
| Munoz, 1995^77^ | 1985-87 | Spain | Hospital | CC | Conv | Women with invasive cervical cancer | ELISA | 195 | 42·1 |
| Munoz, 1995^77^ | 1985-87 | Spain | OC | CC | Conv | Women with cervical intra-epithelial neoplasia | ELISA | 234 | 32·1 |
| Munoz, 1995^77^ | 1985-87 | Spain | Community | CC | Conv | Husbands of women with invasive cervical cancer | ELISA | 108 | 44·4 |
| Munoz, 1995^77^ | 1985-87 | Spain | Community | CC | Conv | Husbands of women with CIN | ELISA | 163 | 36·2 |
| Przybylski, 2017^168^ | 2009-13 | Poland | Hospital | CS | Conv | Transplant patients | EIA | 190 | 10·0 |
| Reinheimer, 2012^169^ | 2000-11 | Germany | Hospital | CS | Conv | Mixed populations at risk | ELISA | 29,694 | 13·6 |
| Shev, 1995^94^ | 1990-92 | Sweden | Community | CC | Conv | HCV positive blood donors | ELISA | 41 | 43·9 |
| Snijders, 2019^98^ | 2011 | Netherlands | Hospital | CC | Conv | Patients with bipolar disorder | ELISA | 760 | 9·3 |
| Vass-Sorensen, 1984^105^ | 1980 | Norway | Hospital | CC | Conv | Women with cervical cancer | WB | 46 | 24·0 |
| Vilibic-Cavlec, 2017^106^ | 2013-15 | Croatia | Hospital | CC | Conv | Hemodialysis patients | WB | 152 | 12·5 |

^a^ The reported study design is the original study design. The included measures in this study are those for the baseline measures at the beginning of the study.

^b^ This study was conducted in Belgium, Denmark, Finland, France, Greece, Italy, Netherlands, Norway, Portugal, Spain, Sweden, and Switzerland.

^c^ This study was conducted in Finland, Iceland, Norway, and Sweden.

Abbreviations: ASD = Autism spectrum disorder, CC = Case-control, CIN = Cervical intraepithelial neoplasia, Conv = Convenience, CS = Cross-sectional, EIA = Enzyme immunoassay, ELISA = Enzyme-linked immunosorbent type-specific assay, FSWs = Female sex workers, GUM = Genitourinary medicine, HBV= Hepatitis B virus, HCV = Hepatitis C virus, HIV = Human immunodeficiency virus, HSV-1 = Herpes simplex virus type 1, HSV-2 = Herpes simplex virus type 2, IVF= *In-vitro* fertilization, MAb = Monoclonal antibody, MSM = Men who have sex with men, OC = Outpatient clinic, RDS = Respondent driven sampling, RS = Random sampling, SRS = Stratified random sampling, STI = Sexually transmitted infection, UK = United Kingdom of Great Britain and Northern Ireland, WB = Western blot.

**Table S7. Summary of the precision and risk of bias (ROB) assessments for the studies reporting HSV-2 seroprevalence in Europe.**

| **Quality assessments** | **HSV-2 seroprevalence measures** | |
| --- | --- | --- |
|  | **Number of studies** | **%** |
| **Precision of seroprevalence measures^a^** | | |
| Low precision | 84 | 28·6 |
| High precision | 210 | 71·4 |
| **Risk of bias quality domain^b^** | | |
| **Sampling method** | | |
| Low risk of bias | 43 | 14·6 |
| High risk of bias | 251 | 85·4 |
| **Response rate** | | |
| Low risk of bias | 18 | 6·1 |
| High risk of bias | 24 | 8·2 |
| Unclear risk of bias | 252 | 85·7 |
| **Summary of the risk of bias assessment** | | |
| **Low risk of bias** |  |  |
| In at least one quality domain | 55 | 18·7 |
| In both quality domains | 6 | 2·0 |
| **High risk of bias** |  |  |
| In at least one quality domain | 260 | 98·0 |
| In both quality domains | 15 | 5·1 |
| **Seroprevalence studies where risk of bias assessment was possible** | **294** | **100** |

^a^ Precision was assessed based on the overall sample size (not each stratum subsample size) of the study as reported in the record/publication.

^b^ Risk of bias was assessed based on the sampling method and response rate of the study as reported in the record/publication.
Abbreviations: HSV-2 = Herpes simplex virus type 2.

**Figure S1. Forest plots for the pooled mean HSV-2 seroprevalence among different at-risk populations in Europe.**

1. **General populations by subregion**
   1. **Eastern Europe**

**
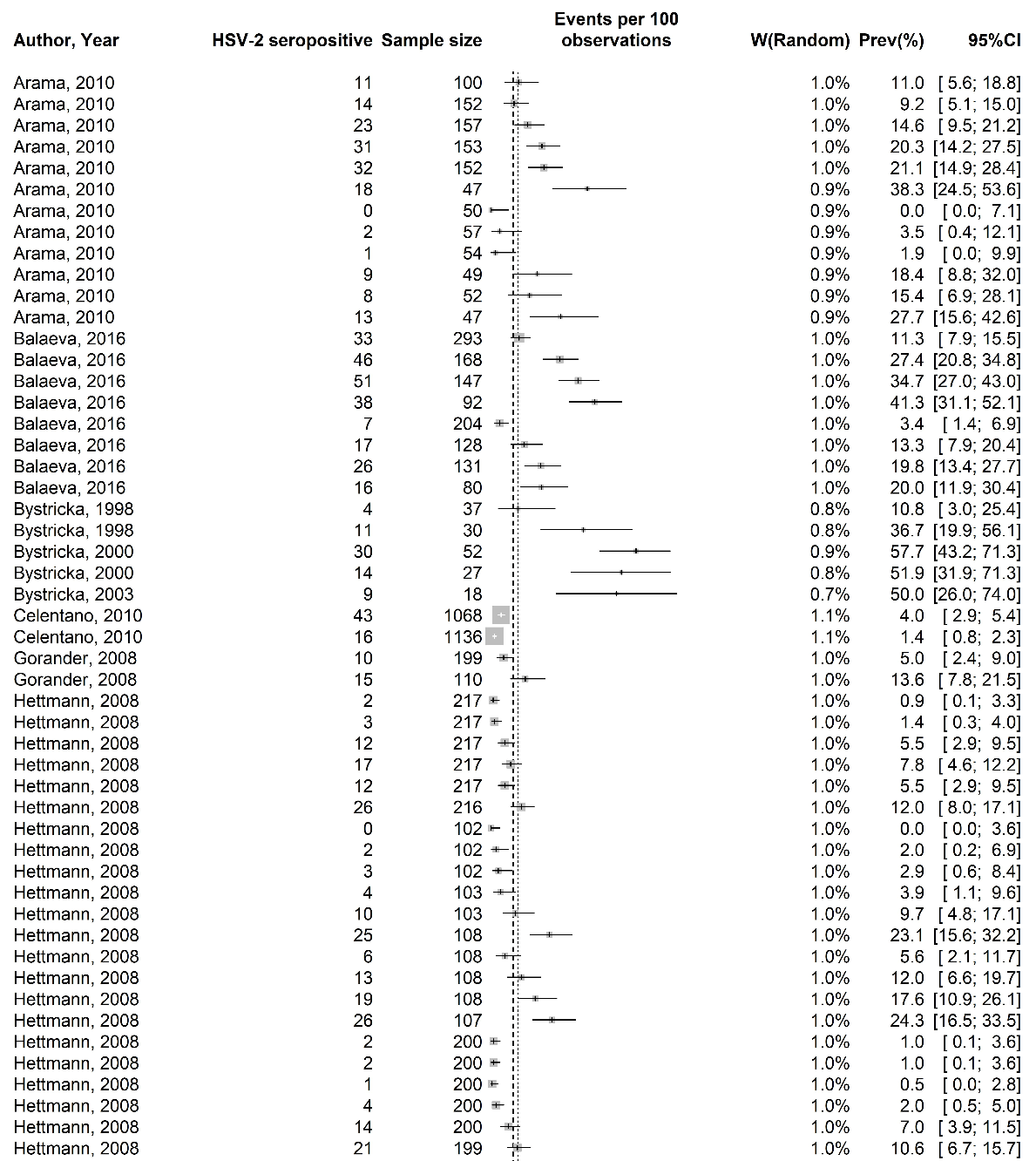
**

**
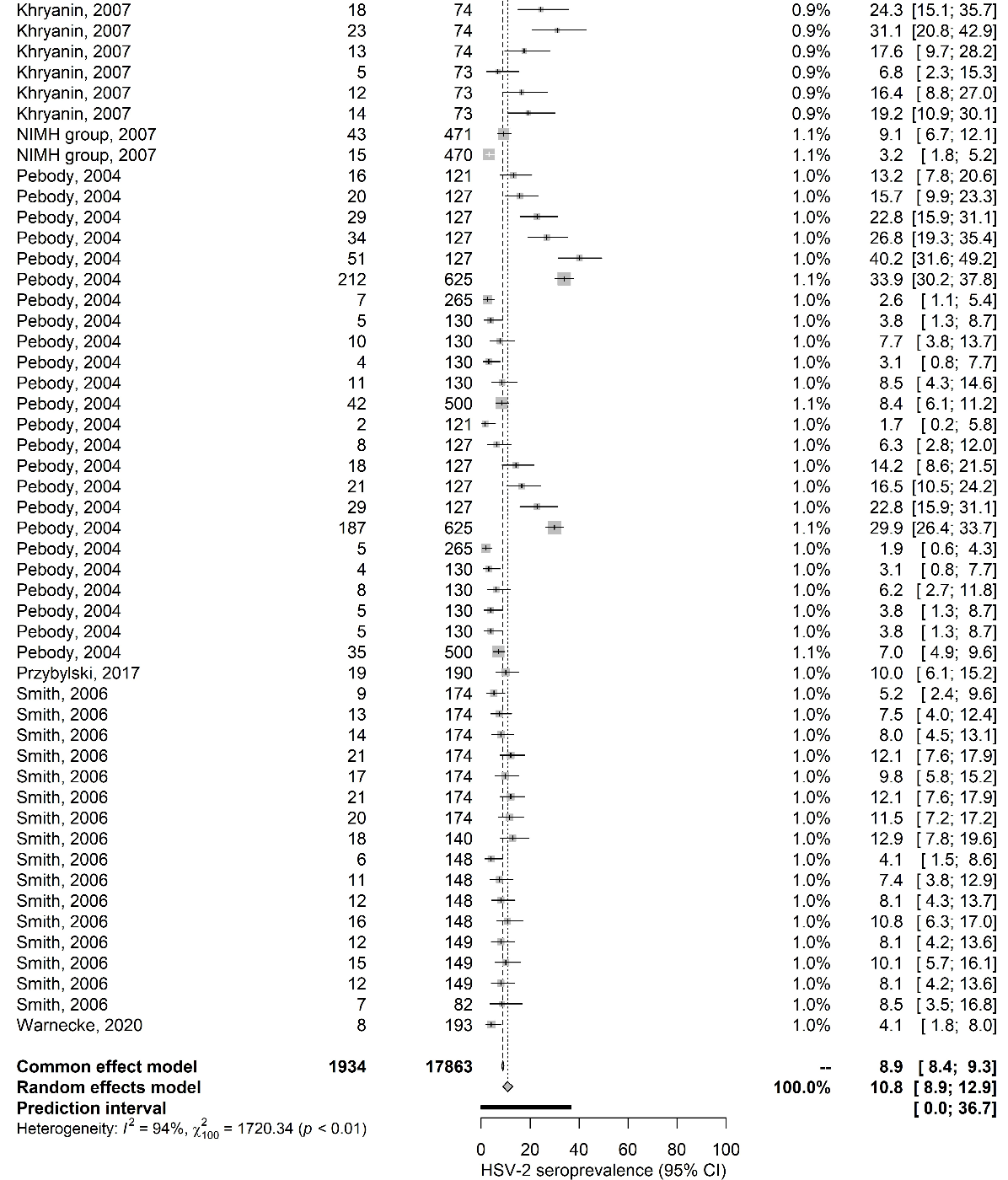
**

- 1. **Southern Europe**

**
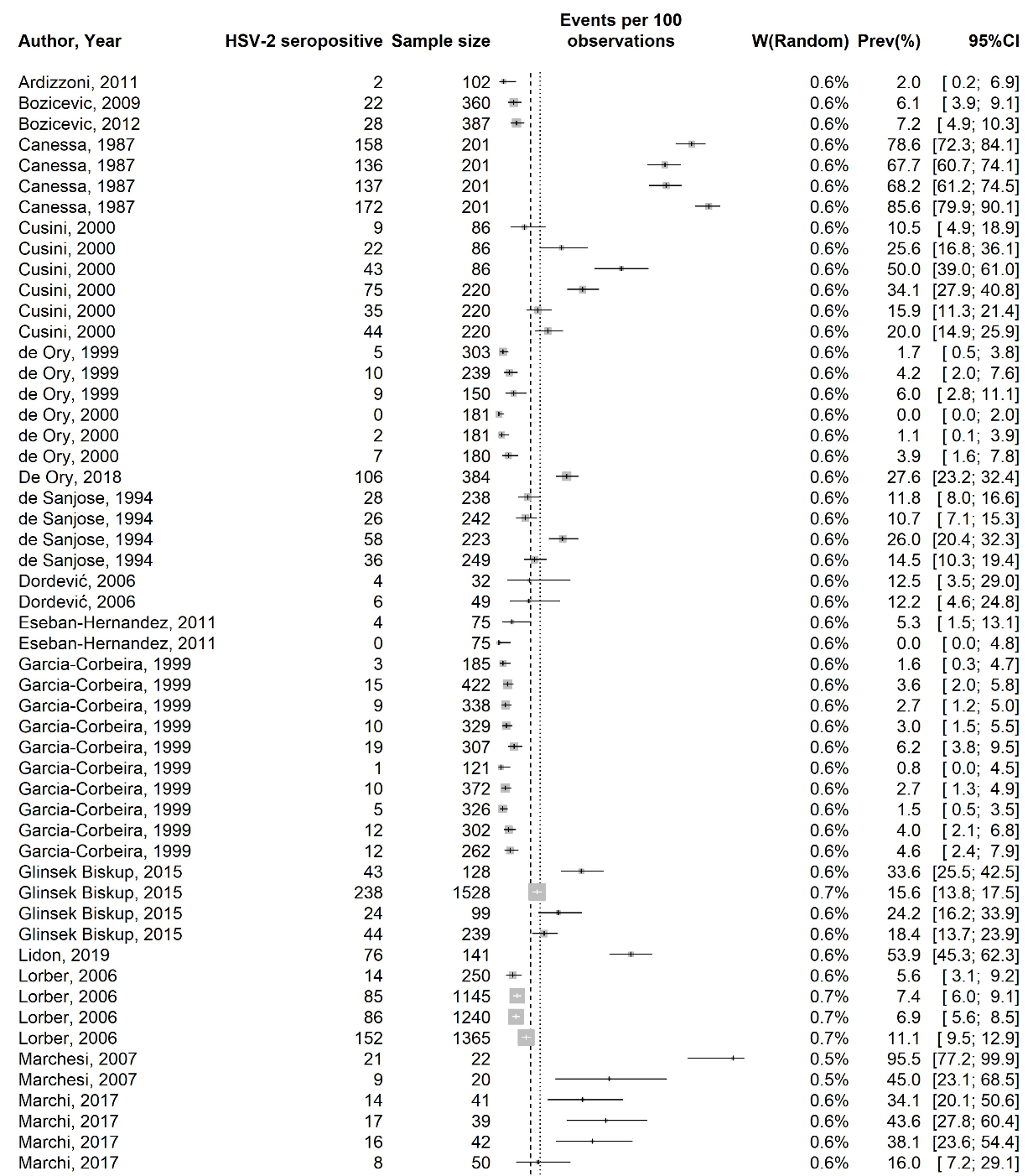
**

**
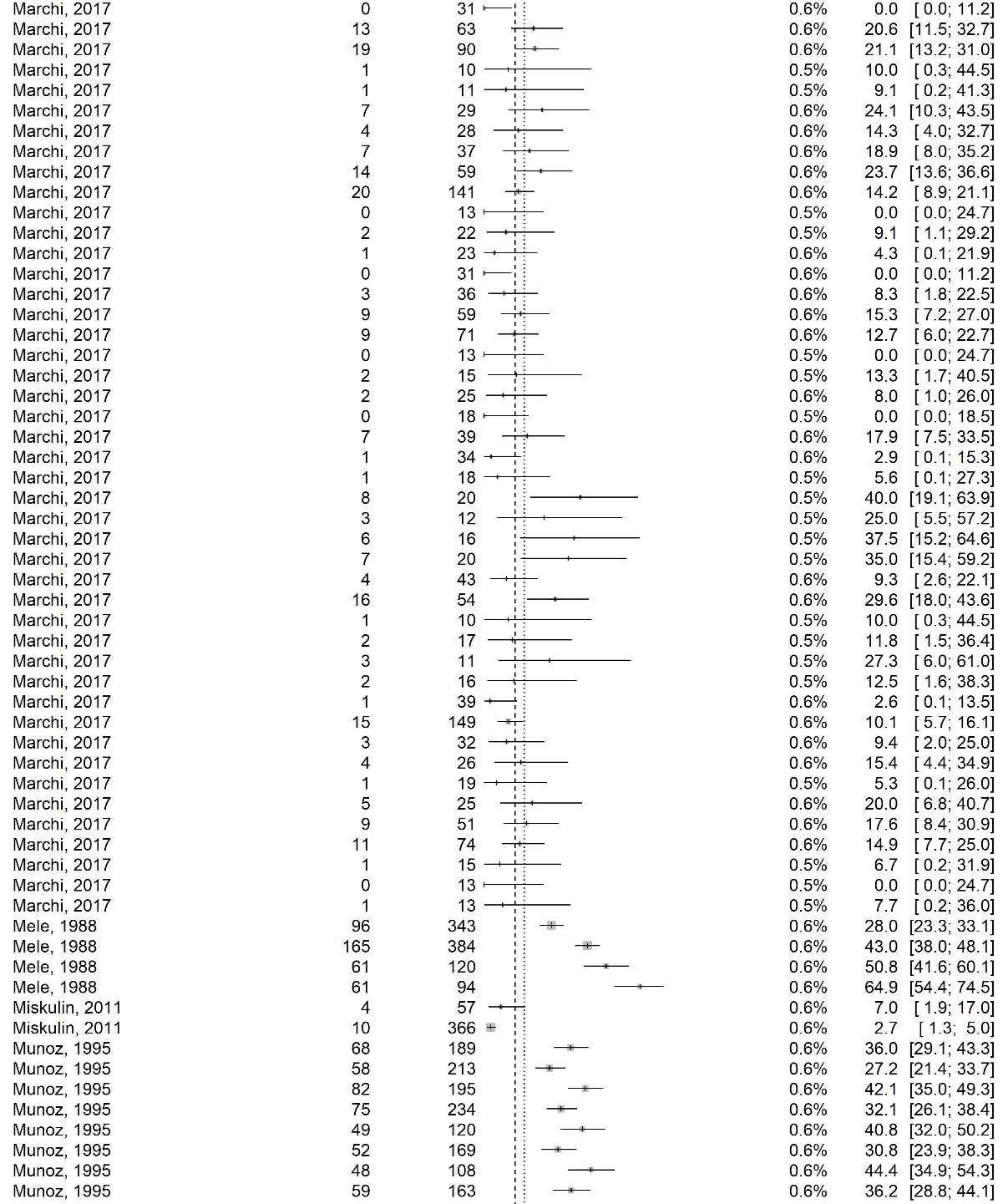
**

**
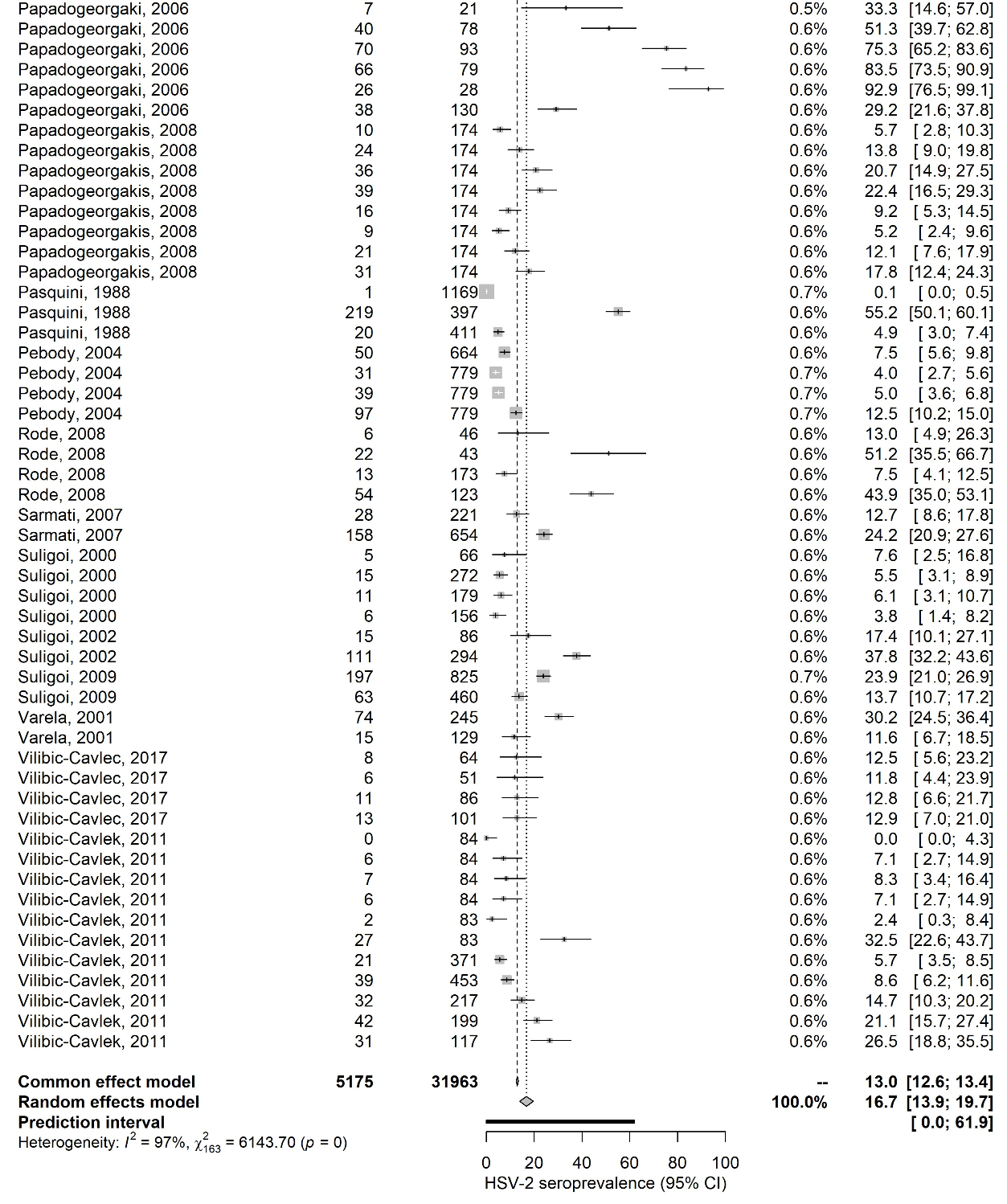
**

- 1. **Western Europe**

**
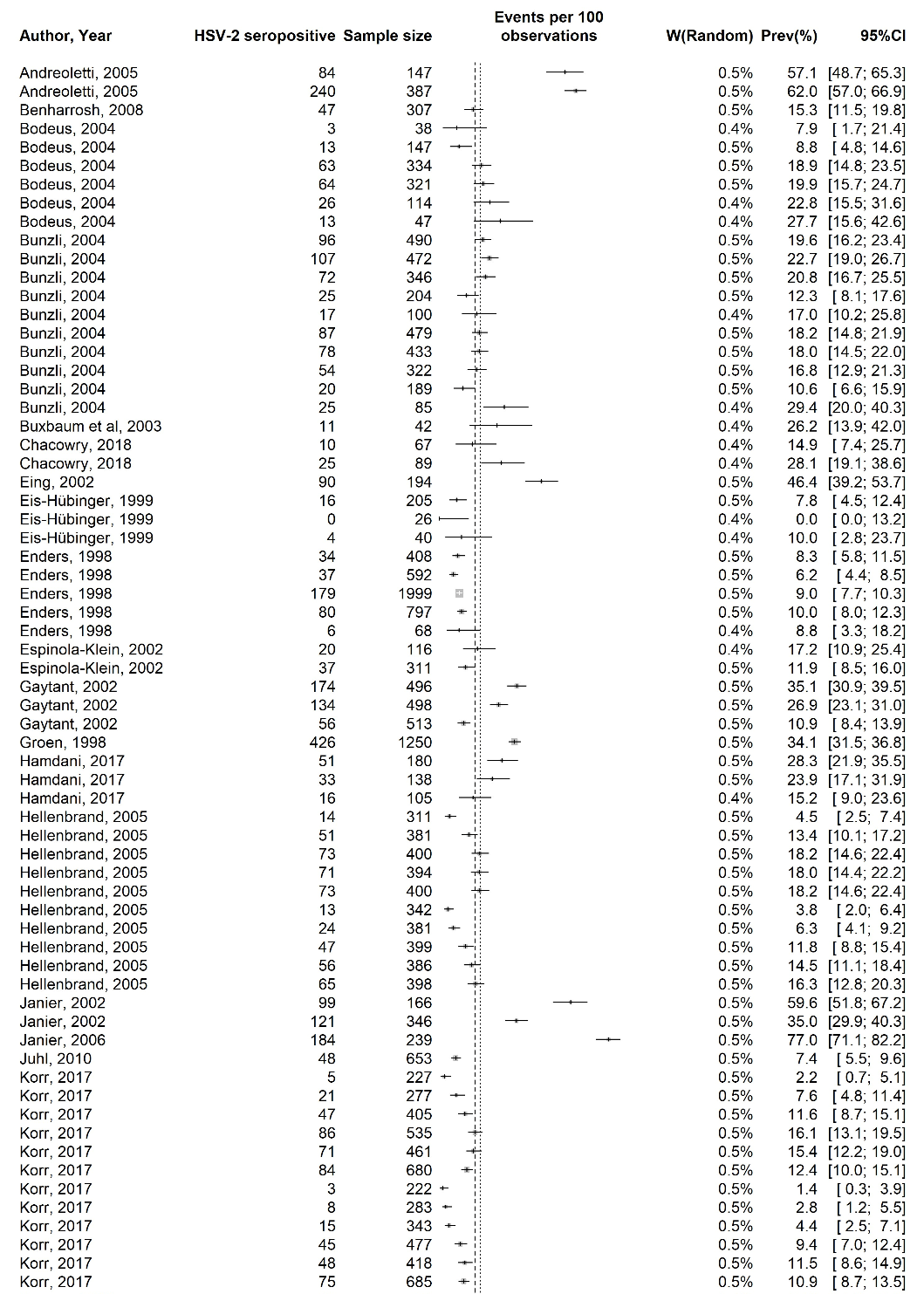
**

**
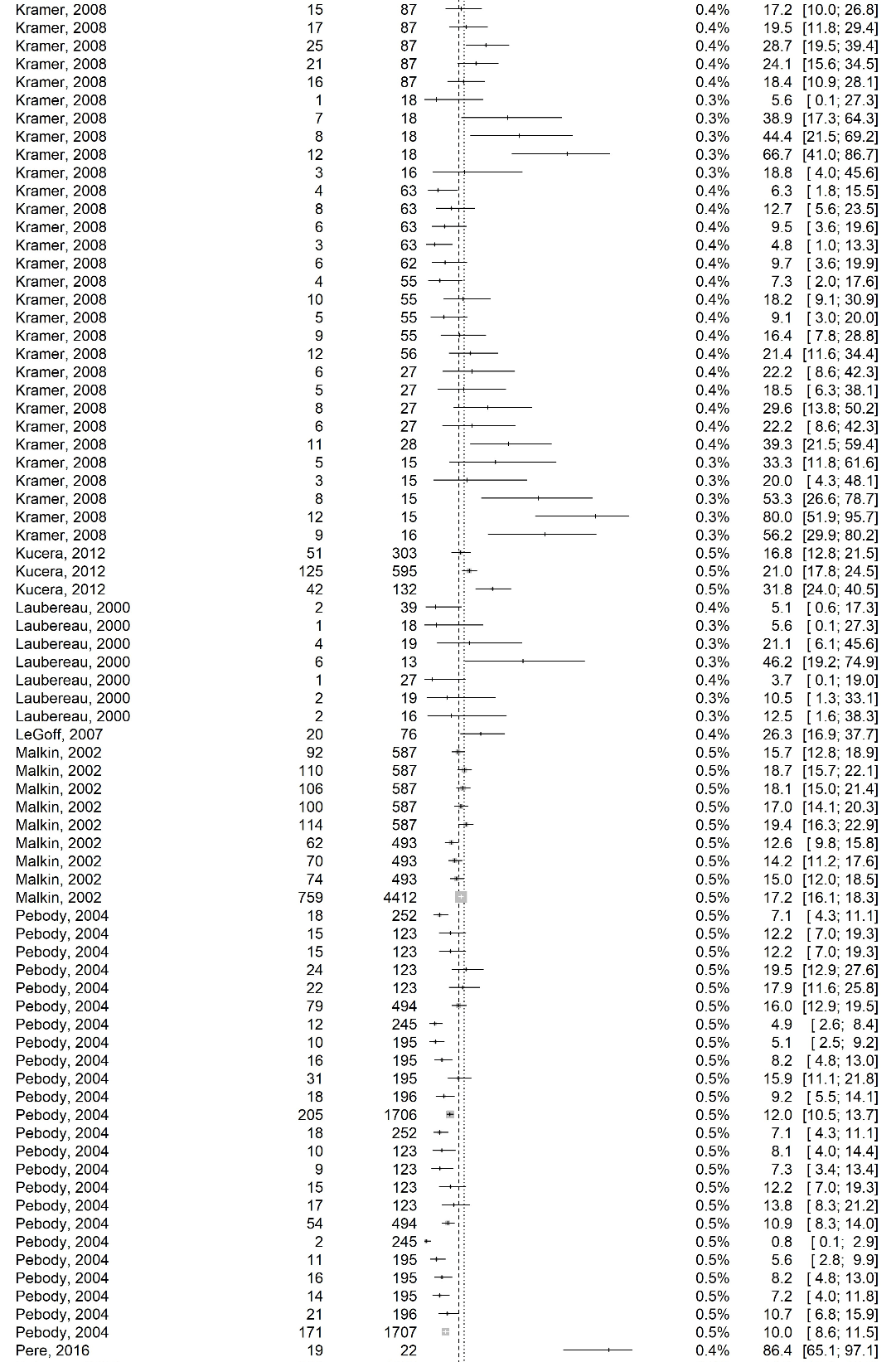
**

**
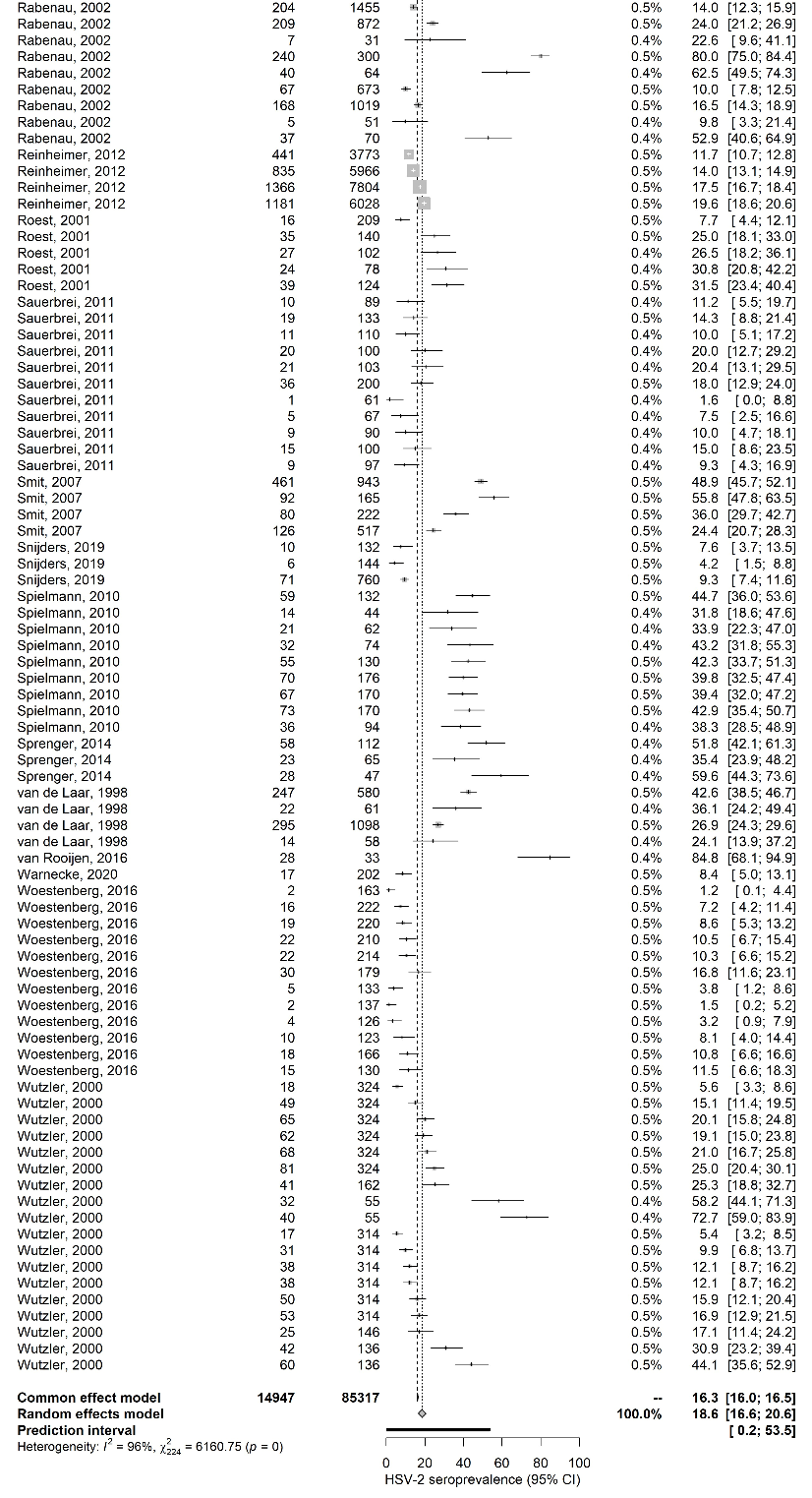
**

- 1. **Northern Europe**

**
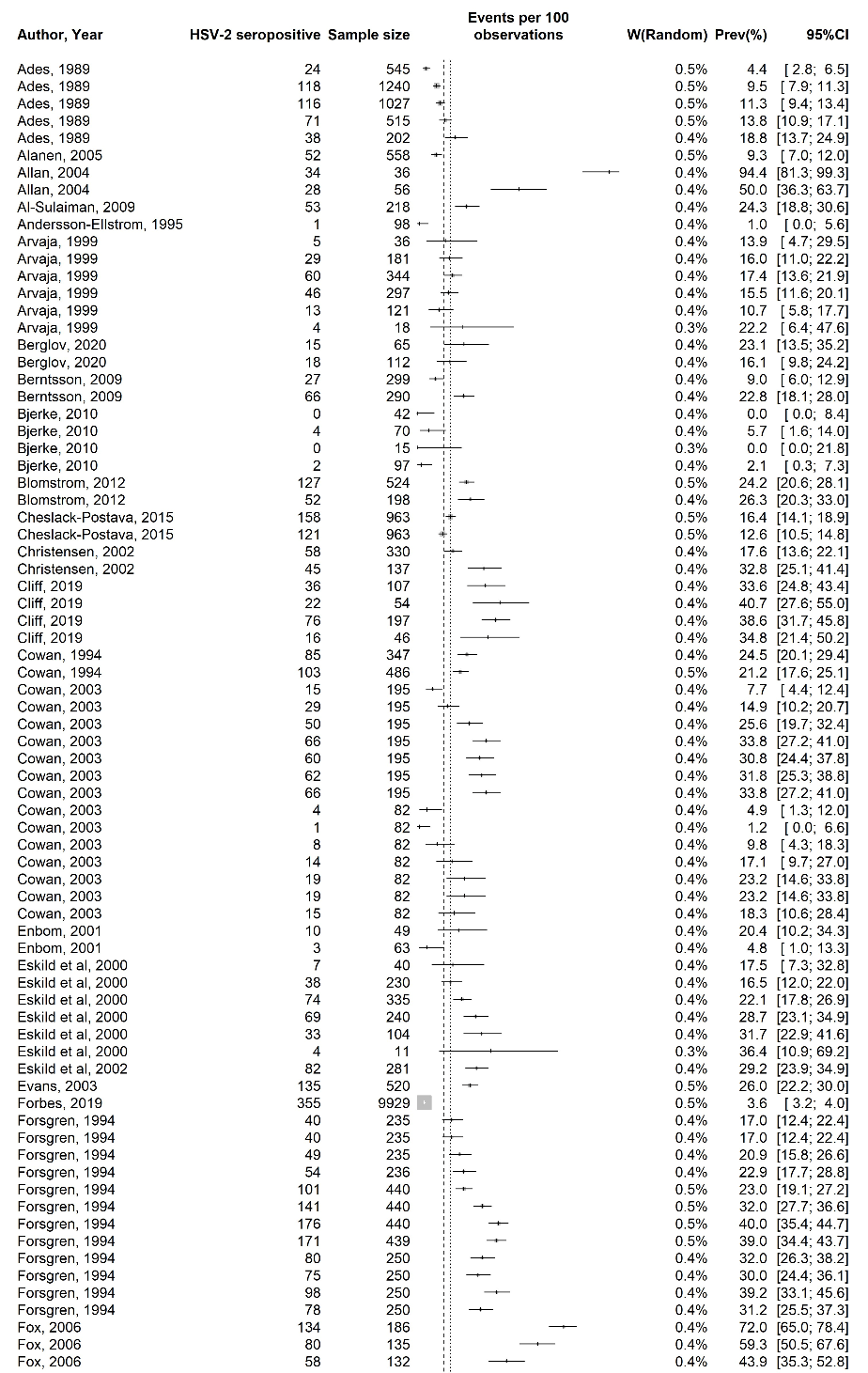
**

**
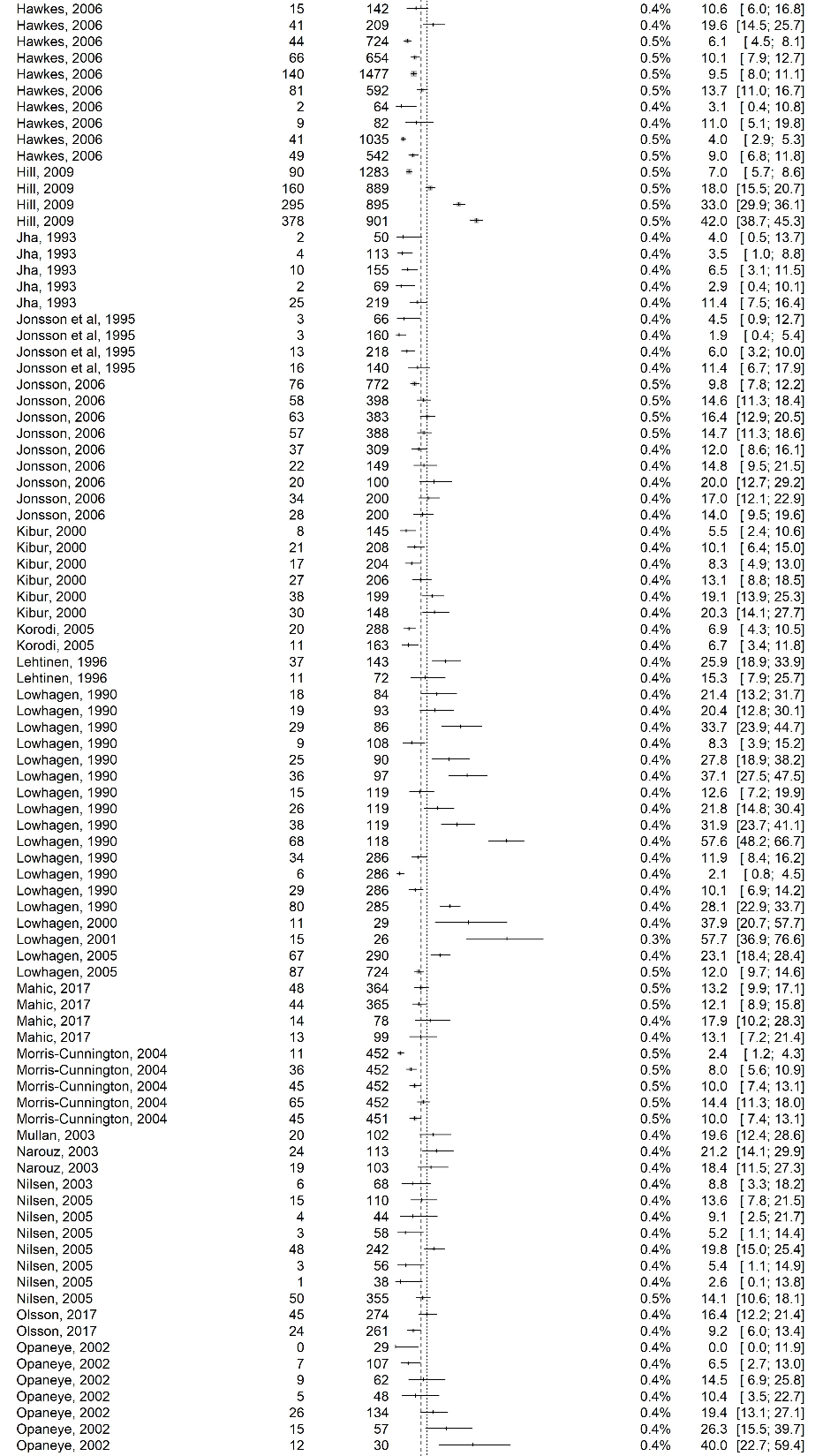
**

**
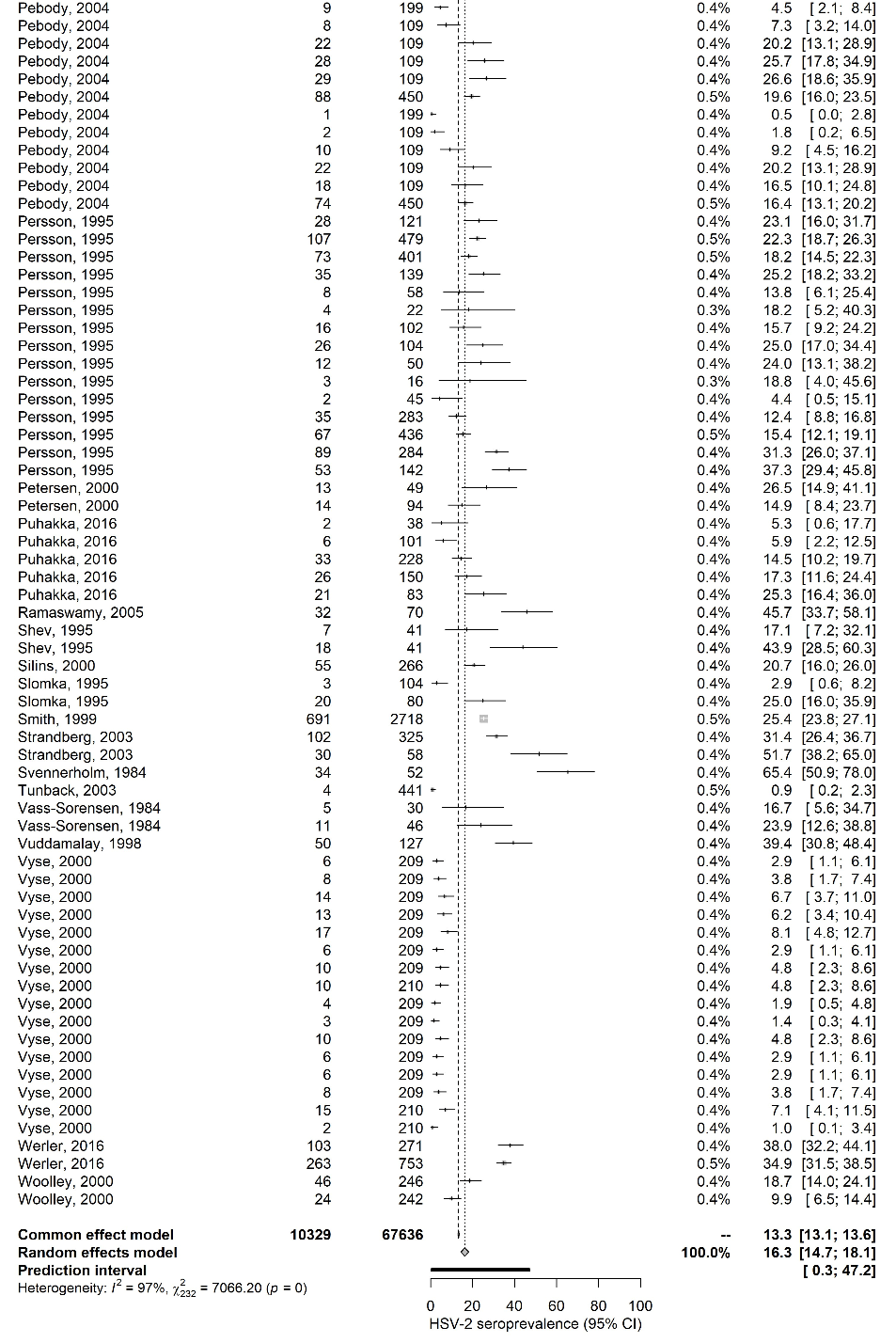
**

- 1. **Mixed regions in Europe**

**
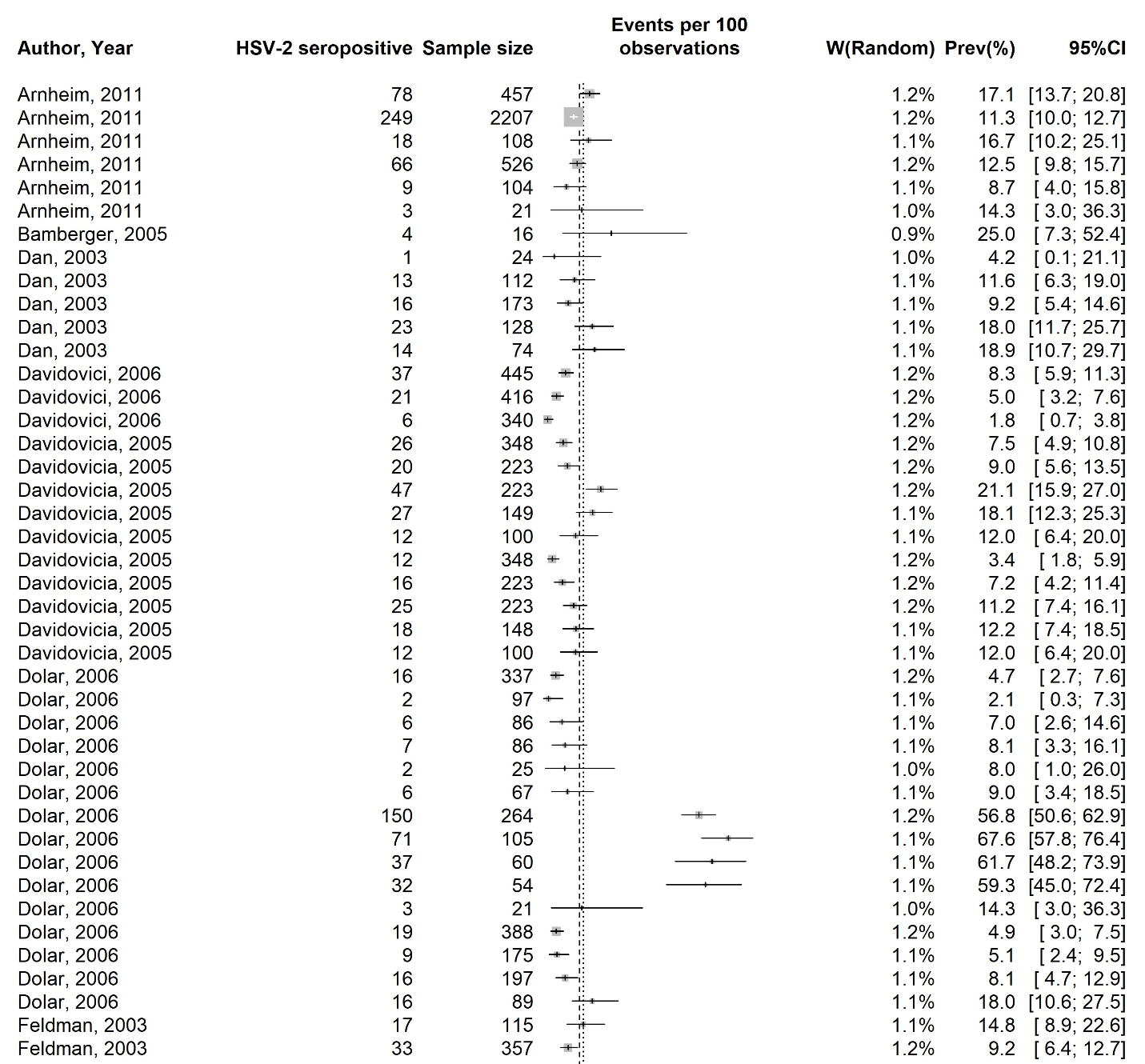
**

**
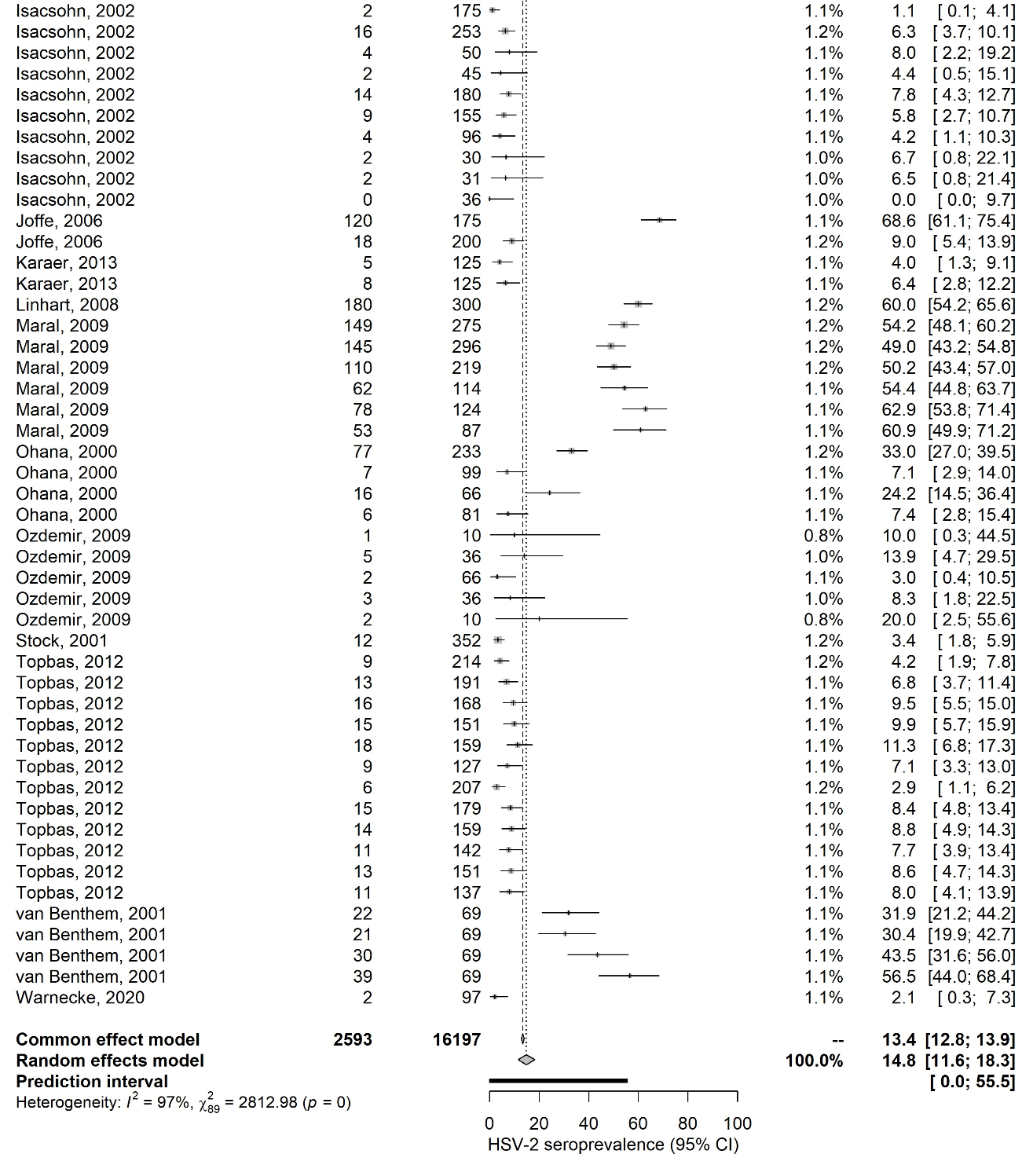
**

1. **Intermediate-risk populations**

**
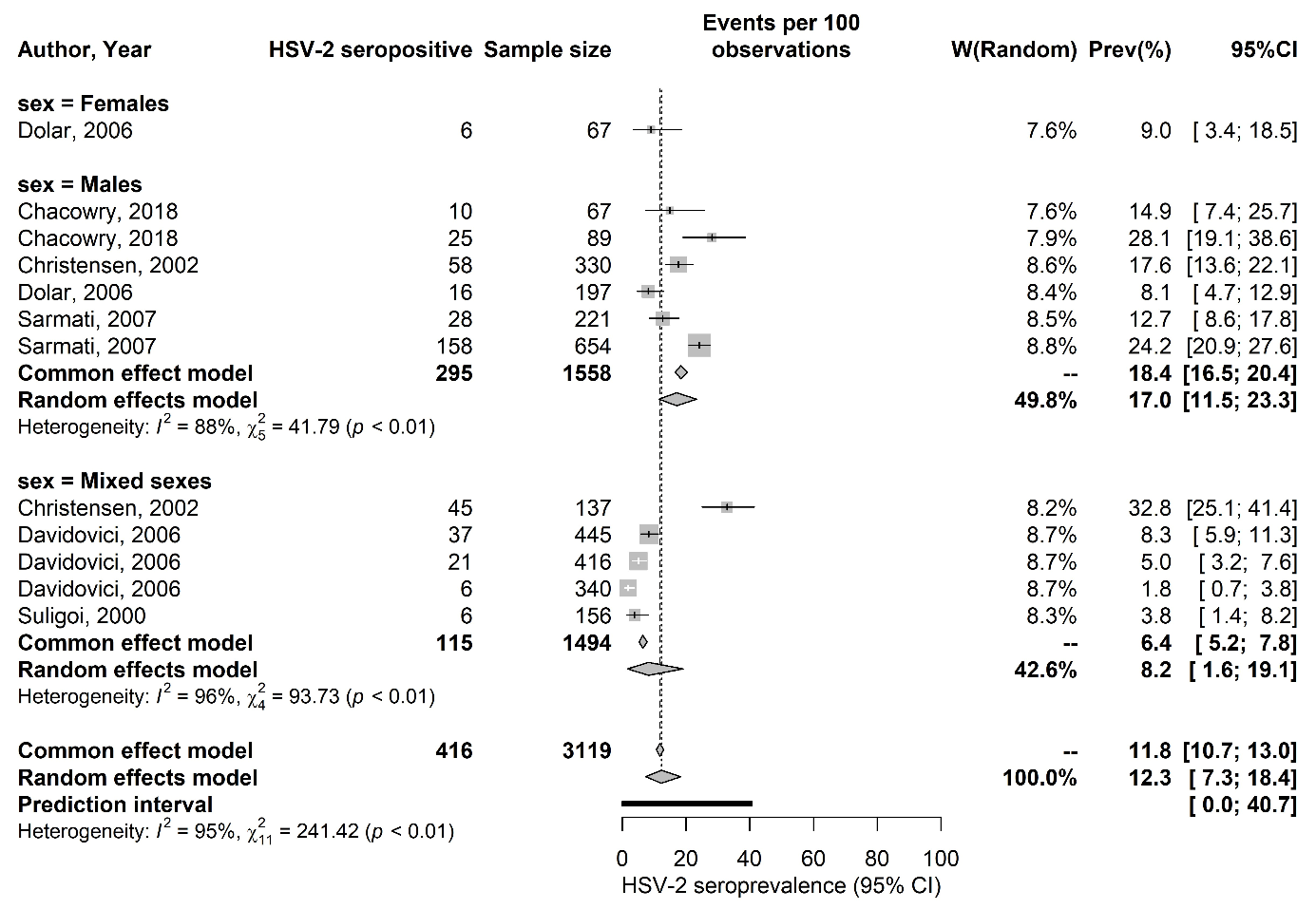
**

1. **Female sex workers**

**
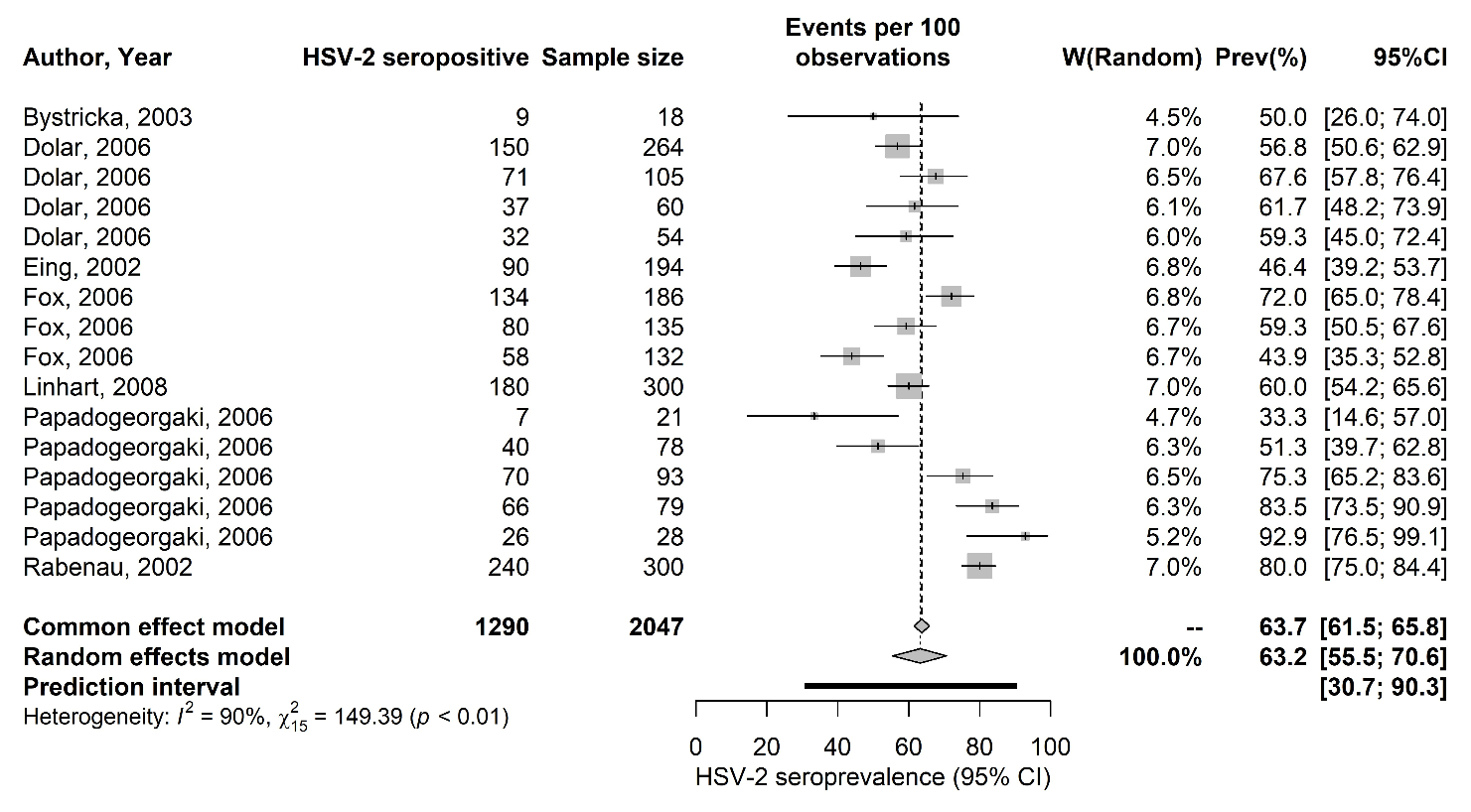
**

1. **Men who have sex with men**

**

**

1.

**Sexually transmitted infection clinic attendees and symptomatic populations**
2. **People living with HIV and people in HIV discordant couples**


3. **Infertility clinic attendees and women with ectopic pregnancies**

**
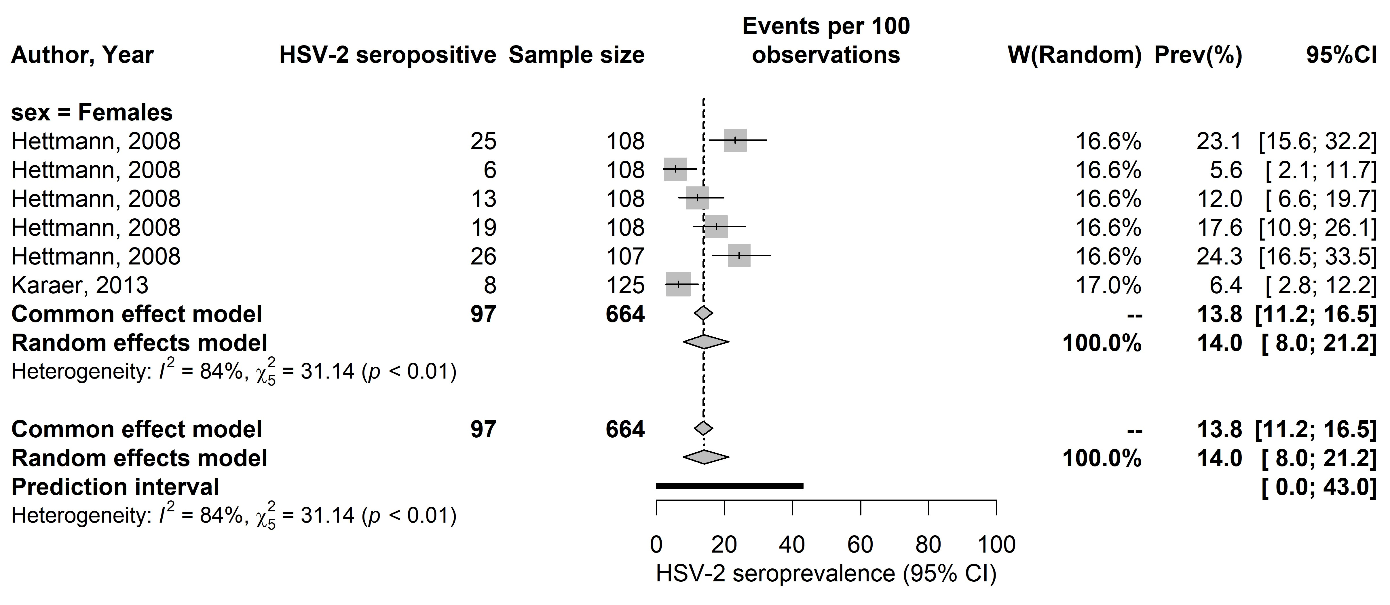
**

1. **Other populations**

**
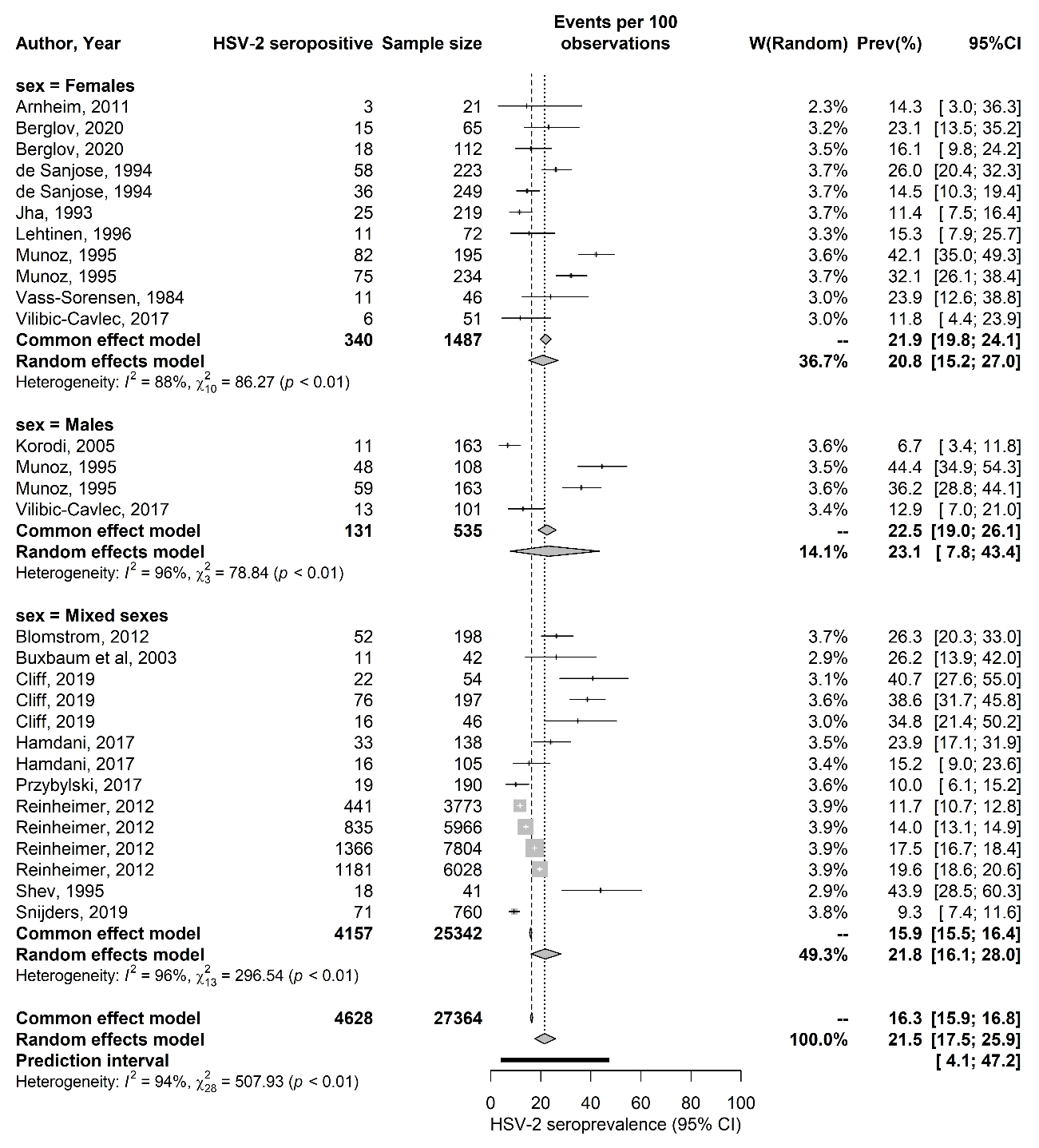
**

**Table S8. Univariable and multivariable meta-regression analyses for HSV-2 seroprevalence in Europe, using the year of publication as the temporal variable instead of the year of data collection.**

|  | | | **Outcome measures** | **Sample size** | **Univariable analysis** | | | | **Multivariable analysis** | | | |
| --- | --- | --- | --- | --- | --- | --- | --- | --- | --- | --- | --- | --- |
|  |  |  |  |  |  |  |  |  | **Model 3^a^: Time as a categorical variable model** | | **Model 4^b^: Time as a linear variable model** | |
|  |  |  | **Total n** | **Total N** | ***RR* (95%CI)** | **p-value** | **LR test p-value^c^** | **Adjusted R^2^ (%)** | ***ARR* (95% CI)** | **p-value** | ***ARR* (95% CI)** | **p-value** |
| **Population characteristics** | **Population type** | General populations | 626 | 154,957 | 1·00 | - | <0·001 | 27·72 | 1·00 | - | 1·00 | - |
|  |  | Intermediate-risk populations | 12 | 3,119 | 0·93 (0·61-1·44) | 0·751 |  |  | 1·07 (0·71-1·62) | 0·737 | 1·07 (0·71-1·61) | 0·740 |
|  |  | FSWs | 16 | 2,047 | 5·13 (3·58-7·33) | <0·001 |  |  | 4·49 (3·20-6·31) | <0·001 | 4·47 (3·20-6·26) | <0·001 |
|  |  | MSM | 12 | 7,011 | 1·99 (1·32-3·00) | <0·001 |  |  | 2·51 (1·69-3·72) | <0·001 | 2·52 (1·70-3·74) | <0·001 |
|  |  | STI clinic attendees and symptomatic populations | 74 | 19,487 | 2·07 (1·73-2·48) | <0·001 |  |  | 1·89 (1·58-2·26) | <0·001 | 1·86 (1·55-2·23) | <0·001 |
|  |  | People living with HIV and people in HIV discordant couples | 35 | 3,660 | 3·69 (2·88-4·72) | <0·001 |  |  | 3·37 (2·64-4·30) | <0·001 | 3·37 (2·64-4·29) | <0·001 |
|  |  | Infertility clinic attendees and women with ectopic pregnancies | 7 | 766 | 0·96 (0·53-1·72) | 0·884 |  |  | 1·00 (0·57-1·74) | 0·987 | 1·00 (0·57-1·75) | 0·996 |
|  |  | Other populations | 31 | 27,929 | 1·70 (1·30-2·22) | <0·001 |  |  | 1·50 (1·15-1·95) | 0·003 | 1·52 (1·18-1·96) | 0·001 |
|  | **Age group** | <20 years | 48 | 7,799 | 1·00 | - | <0·001 | 8·41 | 1·00 | - | 1·00 | - |
|  |  | 20-29 years | 117 | 28,019 | 1·77 (1·29-2·41) | <0·001 |  |  | 1·77 (1·36-2·31) | <0·001 | 1·78 (1·37-2·32) | <0·001 |
|  |  | 30-39 years | 98 | 19,576 | 2·55 (1·85-3·51) | <0·001 |  |  | 2·65 (2·01-3·48) | <0·001 | 2·66 (2·03-3·50) | <0·001 |
|  |  | 40-49 years | 35 | 13,743 | 2·35 (1·59-3·46) | <0·001 |  |  | 2·70 (1·94-3·77) | <0·001 | 2·72 (1·95-3·80) | <0·001 |
|  |  | 50-59 years | 19 | 3,814 | 2·14 (1·35-3·39) | 0·001 |  |  | 2·61 (1·76-3·86) | <0·001 | 2·64 (1·79-3·91) | <0·001 |
|  |  | ≥60 years | 22 | 9,220 | 2·57 (1·63-4·03) | <0·001 |  |  | 2·58 (1·75-3·82) | <0·001 | 2·60 (1·76-3·84) | <0·001 |
|  |  | Mixed ages | 474 | 136,805 | 2·73 (2·06-3·61) | <0·001 |  |  | 2·04 (1·59-2·62) | <0·001 | 2·06 (1·61-2·64) | <0·001 |
|  | **Sex** | Women | 437 | 100,759 | 1·00 | - | <0·001 | 6·10 | 1·00 | - | 1·00 | - |
|  |  | Men | 238 | 55,096 | 0·69 (0·60-0·79) | <0·001 |  |  | 0·65 (0·58-0·74) | <0·001 | 0·65 (0·58-0·74) | <0·001 |
|  |  | Mixed sexes | 138 | 63,121 | 1·26 (1·07-1·49) | 0·005 |  |  | 0·97 (0·83-1·12) | 0·640 | 0·97 (0·84-1·13) | 0·692 |
|  | **European subregion** | Eastern Europe | 101 | 17,863 | 1·00 | - | <0·001 | 3·19 | 1·00 | - | 1·00 | - |
|  |  | Southern Europe | 164 | 31,963 | 1·52 (1·22-1·89) | <0·001 |  |  | 1·19 (0·97-1·45) | 0·095 | 1·18 (0·98-1·43) | 0·085 |
|  |  | Western Europe | 225 | 85,317 | 1·68 (1·37-2·07) | <0·001 |  |  | 1·35 (1·13-1·62) | 0·001 | 1·33 (1·11-1·59) | 0·002 |
|  |  | Northern Europe | 233 | 67,636 | 1·51 (1·23-1·85) | <0·001 |  |  | 1·20 (1·00-1·44) | 0·051 | 1·18 (0·98-1·41) | 0·074 |
|  |  | Israel, Turkey, and Mixed regions | 90 | 16,197 | 1·26 (0·98-1·62) | 0·076 |  |  | 0·92 (0·74-1·14) | 0·447 | 0·90 (0·73-1·12) | 0·359 |
|  | **Country’s income** | UMIC | 87 | 15,429 | 1·00 | - | 0·326 | 0·03 | - | - | - | - |
|  |  | HIC | 721 | 202,919 | 1·03 (0·84-1·26) | 0·769 |  |  | - | - | - | - |
|  |  | Mixed Income | 5 | 628 | 1·80 (0·84-3·89) | 0·132 |  |  | - | - | - | - |
| **Study methodology characteristics** | **Assay type** | Western Blot | 80 | 23,545 | 1·00 | - | 0·541 | 0·00 | - | - | - | - |
|  |  | ELISA | 725 | 190,632 | 0·99 (0·80-1·24) | 0·940 |  |  | - | - | - | - |
|  |  | Monoclonal antibody | 8 | 4,799 | 1·39 (0·74-2·60) | 0·306 |  |  | - | - | - | - |
|  | **Sample size^d^** | <200 | 106 | 6,947 | 1·00 | - | 0·013 | 1·24 | 1·00 | - | 1·00 | - |
|  |  | ≥200 | 707 | 212,029 | 0·78 (0·65-0·95) | 0·013 |  |  | 0·99 (0·84-1·17) | 0·905 | 0·99 (0·84-1·17) | 0·919 |
|  | **Sampling method** | Probability-based | 210 | 62,803 | 1·00 | - | <0·001 | 3·04 | 1·00 | - | 1·00 | - |
|  |  | Non-probability-based | 603 | 156,173 | 1·31 (1·15-1·51) | <0·001 |  |  | 1·19 (1·04-1·36) | 0·013 | 1·19 (1·04-1·37) | 0·010 |
|  | **Response rate** | ≥80% | 41 | 13,331 | 1·00 | - | 0·009 | 1·09 | 1·00 | - | 1·00 | - |
|  |  | <80% | 102 | 28,738 | 1·06 (0·78-1·44) | 0·716 |  |  | 1·01 (0·78-1·31) | 0·932 | 1·02 (0·79-1·32) | 0·860 |
|  |  | Unclear | 670 | 176,907 | 0·82 (0·62-1·07) | 0·135 |  |  | 0·83 (0·66-1·04) | 0·099 | 0·82 (0·66-1·03) | 0·091 |
| **Temporal variables** | **Year of publication category** | <2000 | 126 | 35,629 | 1·00 | - | 0·121 | 0·52 | 1·00 | - | - | - |
|  |  | 2000-2010 | 500 | 117,355 | 0·87 (0·74-1·04) | 0·122 |  |  | 0·91 (0·78-1·06) | 0·208 | - | - |
|  |  | >2010 | 187 | 65,992 | 0·81 (0·66-0·99) | 0·041 |  |  | 0·86 (0·72-1·02) | 0·076 | - | - |
|  | **Year of publication** | | 813 | 218,976 | 0·99 (0·98-1·00) | 0·035 | 0·035 | 0·68 | - | - | 0·99 (0·98-0·99) | 0·035 |

^a^ Variance explained by multivariable model 3 (adjusted *R^2^*) = 40·15%.

^b^ Variance explained by multivariable model 4 (adjusted *R^2^*) = 40·45%.

^c^ Factors in the univariable analyses with a p-value<0.1 were included in the multivariable analysis.

^d^ Sample size denotes the sample size of each study population found in the original publication.

Abbreviations: ARR = Adjusted risk ratio, CI = Confidence interval*,* ELISA = Enzyme-linked immunosorbent type-specific assay, FSWs = Female sex workers, HIC = High-income countries, HIV = Human immunodeficiency virus, HSV-2 = Herpes simplex virus type 2, LR = Likelihood ratio, MSM = Men who have sex with men, RR = Risk ratio, STI = Sexually transmitted infection, UMIC = Upper-middle-income countries.

**Table S9. Univariable and multivariable meta-regression analyses for HSV-2 seroprevalence among the general populations in Europe.**

|  | | | **Outcome measures** | **Sample size** | **Univariable analysis** | | | | **Multivariable analysis** | | | |
| --- | --- | --- | --- | --- | --- | --- | --- | --- | --- | --- | --- | --- |
|  |  |  |  |  |  |  |  |  | **Model 1^a^: Time as a categorical variable model** | | **Model 2^b^: Time as a linear variable model** | |
|  |  |  | **Total n** | **Total N** | ***RR* (95%CI)** | **p-value** | **LR test p-value^c^** | **Adjusted R^2^ (%)** | ***ARR* (95% CI)** | **p-value** | ***ARR* (95% CI)** | **p-value** |
| **Population characteristics** | **Age group** | <20 years | 45 | 7,346 | 1·00 | - | <0·001 | 5·85 | 1·00 | - | 1·00 | - |
|  |  | 20-29 years | 109 | 23,057 | 1·67 (1·23-2·27) | 0·001 |  |  | 1·77 (1·33-2·35) | <0·001 | 1·75 (1·31-2·33) | <0·001 |
|  |  | 30-39 years | 92 | 13,291 | 2·40 (1·76-3·28) | <0·001 |  |  | 2·73 (2·03-3·66) | <0·001 | 2·68 (1·99-3·60) | <0·001 |
|  |  | 40-49 years | 34 | 5,939 | 2·33 (1·60-3·39) | <0·001 |  |  | 2·75 (1·93-3·93) | <0·001 | 2·73 (1·90-3·90) | <0·001 |
|  |  | 50-59 years | 19 | 3,814 | 2·13 (1·38-3·30) | 0·001 |  |  | 2·51 (1·66-3·79) | <0·001 | 2·59 (1·70-3·93) | <0·001 |
|  |  | ≥60 years | 21 | 3,192 | 2·54 (1·64-3·94) | <0·001 |  |  | 2·42 (1·59-3·68) | <0·001 | 2·42 (1·58-3·70) | <0·001 |
|  |  | Mixed ages | 306 | 98,318 | 2·00 (1·52-2·65) | <0·001 |  |  | 1·95 (1·49-2·54) | <0·001 | 1·97 (1·50-2·58) | <0·001 |
|  | **Sex** | Women | 359 | 88,907 | 1·00 | - | <0·001 | 7·98 | 1·00 | - | 1·00 | - |
|  |  | Men | 186 | 38,478 | 0·63 (0·54-0·73) | <0·001 |  |  | 0·60 (0·53-0·70) | <0·001 | 0·62 (0·53-0·71) | <0·001 |
|  |  | Mixed sexes | 81 | 27,572 | 1·12 (0·93-1·36) | 0·235 |  |  | 0·98 (0·81-1·19) | 0·854 | 1·00 (0·82-1·22) | 0·998 |
|  | **European subregion** | Eastern Europe | 90 | 16,897 | 1·00 | - | 0·004 | 1·87 | 1·00 | - | 1·00 | - |
|  |  | Southern Europe | 121 | 22,635 | 1·28 (1·02-1·62) | 0·034 |  |  | 1·29 (1·03-1·60) | 0·026 | 1·20 (0·96-1·50) | 0·104 |
|  |  | Western Europe | 174 | 51,372 | 1·44 (1·17-1·77) | 0·001 |  |  | 1·29 (1·05-1·57) | 0·013 | 1·28 (1·04-1·56) | 0·017 |
|  |  | Northern Europe | 176 | 52,064 | 1·40 (1·13-1·72) | 0·002 |  |  | 1·40 (1·14-1·72) | 0·001 | 1·23 (1·00-1·52) | 0·053 |
|  |  | Israel, Turkey, and Mixed regions | 65 | 11,989 | 1·11 (0·85-1·45) | 0·440 |  |  | 1·00 (0·78-1·28) | 0·995 | 0·98 (0·76-1·26) | 0·850 |
|  | **Country’s income** | UMIC | 78 | 14,447 | 1·00 | - | 0·152 | 0·51 | - | - | - | - |
|  |  | HIC | 547 | 140,158 | 0·89 (0·73-1·08) | 0·228 |  |  | - | - | - | - |
|  |  | Mixed Income | 1 | 352 | 0·26 (0·05-1·30) | 0·101 |  |  | - | - | - | - |
| **Study methodology characteristics** | **Assay type** | Western Blot | 48 | 17,238 | 1·00 | - | 0·325 | 0·07 | - | - | - | - |
|  |  | ELISA | 577 | 137,615 | 1·04 (0·82-1·33) | 0·736 |  |  | - | - | - | - |
|  |  | Monoclonal antibody | 1 | 104 | 0·26 (0·04-1·70) | 0·158 |  |  | - | - | - | - |
|  | **Sample size^d^** | <200 | 61 | 3,306 | 1·00 | - | 0·513 | 0·00 | - | - | - | - |
|  |  | ≥200 | 565 | 151,651 | 1·09 (0·84-1·41) | 0·513 |  |  | - | - | - | - |
|  | **Sampling method** | Probability-based | 199 | 57,435 | 1·00 | - | 0·648 | 0·00 | - | - | - | - |
|  |  | Non-probability-based | 427 | 97,522 | 1·03 (0·90-1·19) | 0·648 |  |  | - | - | - | - |
|  | **Response rate** | ≥80% | 26 | 8,472 | 1·00 | - | 0·002 | 2·54 | 1·00 | - | 1·00 | - |
|  |  | <80% | 79 | 20,909 | 1·30 (0·92-1·84) | 0·142 |  |  | 1·15 (0·82-1·59) | 0·419 | 1·12 (0·80-1·56) | 0·505 |
|  |  | Unclear | 521 | 125,576 | 0·91 (0·67-1·24) | 0·564 |  |  | 0·83 (0·62-1·12) | 0·222 | 0·84 (0·62-1·12) | 0·239 |
| **Temporal variables** | **Year of data collection category** | <1995 | 166 | 46,557 | 1·00 | - | 0·027 | 0·96 | 1·00 | - | - | - |
|  |  | 1995-2005 | 373 | 91,650 | 1·14 (0·98-1·33) | 0·081 |  |  | 1·27 (1·09-1·47) | 0·002 | - | - |
|  |  | >2005 | 87 | 16,750 | 0·90 (0·72-1·11) | 0·321 |  |  | 0·92 (0·74-1·14) | 0·450 | - | - |
|  | **Year of data collection** | | 626 | 154,957 | 0·99 (0·99-1·00) | 0·130 | 0·130 | 0·41 | - | - | 0·99 (0·98-1·00) | 0·110 |

^a^ Variance explained by multivariable model 1 (adjusted *R^2^*) = 20.17%.

^b^ Variance explained by multivariable model 2 (adjusted *R^2^*) =18.12%.

^c^ Factors in the univariable analyses with a p-value<0.1 were included in the multivariable analysis.

^d^ Sample size denotes the sample size of each study population found in the original publication.

Abbreviations: ARR = Adjusted risk ratio, CI = Confidence interval*,* ELISA = Enzyme-linked immunosorbent type-specific assay, HIC = High-income countries, HSV-2 = Herpes simplex virus type 2, LR = Likelihood ratio, RR = Risk ratio, UMIC = Upper-middle-income countries.

**Table S10. Studies reporting proportions of HSV-2 virus isolation in clinically diagnosed genital ulcer disease and in laboratory-confirmed genital herpes in Europe.**

| **Author, year** | **Year(s) of data collection** | **Country** | **Study site** | **Study design** | **Sampling method** | **Population** | **HSV-2 biological assay** | **Sample size** | **Proportion of HSV-2 detection (%)** |
| --- | --- | --- | --- | --- | --- | --- | --- | --- | --- |
| **Patients with clinically diagnosed GUD** | | |  |  |  |  |  |  |  |
| Aldea, 2002^170^ | - | Spain | OC | CS | Conv | Patients with GUD | Culture | 118 | 15·3 |
| Bruisten, 2001^171^ | 1996 | Netherlands | OC | CS | Conv | Patients with GUD | PCR | 372 | 48·1 |
| Doric, 2012^172^ | 2008-09 | Croatia | OC | CS | Conv | STI clinic attendees | PCR | 180 | 21·7 |
| Glinsek Biskup, 2015^136^ | 2009-14 | Slovenia | OC | CS | Conv | STI clinic attendees | PCR | 174 | 20·7 |
| Grange, 2020^173^ | 2010-16 | France | OC | CS | Conv | STI clinic attendees suspected of primary syphilis | PCR | 117 | 2·6 |
| Grange, 2020^173^ | 2010-16 | France | OC | CS | Conv | STI clinic attendees suspected of secondary syphilis | PCR | 50 | 2·0 |
| Grange, 2020^173^ | 2010-16 | France | OC | CS | Conv | STI clinic attendees with anogenital lesions | PCR | 148 | 22·3 |
| Hope-Rapp, 2010^174^ | 1995-05 | France | OC | CS | Conv | Patients with GUD | PCR | 278 | 15·8 |
| Janier, 2006^139^ | 1999-00 | France | OC | CS | Conv | Patients with genital lesions | PCR | 464 | 45·7 |
| Kortekangas-Savolainen, 2014^175^ | 2005-12 | Finland | OC | CS | Conv | STI clinic attendees | MAb | 1,762 | 31·6 |
| Pérez-Torralba, 2020^176^ | 2018-19 | Spain | OC | CS | Conv | Patients with GUD | PCR | 162 | 19·1 |
| Scieux, 1989^177^ | 1981-86 | France | OC | CS | Conv | Patients with GUD | MAb | 154 | 23·4 |
| Slomka, 1998^178^ | - | UK | OC | CS | Conv | Patients with GUD | PCR | 194 | 36·6 |
| **Patients with laboratory-confirmed genital herpes** | | | | | | | | | |
| Aldea, 2002^170^ | - | Spain | OC | CS | Conv | Patients with genital herpes | Culture | 28 | 64·0 |
| Al-Hasani, 1986^179^ | - | UK | OC | CS | Conv | Patients with first-episode genital herpes | Culture | 12 | 41·7 |
| Barton, 1982^180^ | 1980 | UK | OC | CS | Conv | Patients with genital herpes | GE | 31 | 38·7 |
| Challenor, 2007^181^ | 2005 | UK | OC | CS | Conv | Patients with first-episode genital herpes | Culture | 1,203 | 54·4 |
| Christie, 1997^182^ | 1995 | UK | OC | CS | Conv | GUM clinic attendees | Culture | 116 | 41·4 |
| Christie, 1997^182^ | 1982-84 | UK | OC | CS | Conv | GUM clinic attendees | Culture | 127 | 70·1 |
| Coyle, 2003^183^ | 1995-01 | Ireland | OC | CS | Conv | Patients with recurrent genital herpes | PCR | 69 | 50·7 |
| Doric, 2012^172^ | 2008-09 | Croatia | OC | CS | Conv | STI clinic attendees | PCR | 64 | 60·9 |
| Dundarov, 1980^184^ | - | Bulgaria | OC | CS | Conv | Patients with genital lesions | Culture | 287 | 100 |
| Edwards, 1994^185^ | 1992-94 | UK | OC | CS | Conv | Patients with first-episode genital herpes | MAb | 49 | 30·6 |
| Filen, 2004^186^ | 2001-02 | Sweden | OC | CS | Conv | STI clinic attendees | PCR | 51 | 47·1 |
| Grillner, 1983^187^ | - | Sweden | OC | CS | Conv | Patients with symptoms of herpes infection | Culture | 106 | 94·3 |
| Harrison, 2020^188^ | 2016-18 | UK | OC | CS | Conv | Patients with first-episode genital herpes | PCR | 2,864 | 47·5 |
| Hope-Rapp, 2010^174^ | 1995-05 | France | OC | CS | Conv | Patients with genital herpes | PCR | 70 | 62·9 |
| Janier, 2006^139^ | 1999-02 | France | OC | CS | Conv | Patients with genital herpes | PCR | 248 | 85·5 |
| Juhl, 2010^54^ | - | Germany | Hospital | CS | Conv | Patients with genital herpes | PCR | 15 | 53·3 |
| Kortekangas- Savolainen, 2007^189^ | 1994-02 | Finland | OC | CS | Conv | STI clinic attendees | MAb | 617 | 79·3 |
| Kortekangas-Savolainen, 2014^175^ | 2003-12 | Finland | OC | CS | Conv | STI clinic attendees | MAb | 839 | 66·4 |
| Lowhagen, 1990^69^ | 1980-87 | Sweden | OC | CS | Conv | STI clinic attendees in Göteborg | MAb | 1,087 | 89·6 |
| Lowhagen, 2000^141^ | 1995-99 | Sweden | OC | CS | Conv | Patients with first-episode genital herpes | Culture | 97 | 57·0 |
| Lowhagen, 2002^190^ | 1994-98 | Sweden | OC | CS | Conv | STI clinic attendees | ELISA | 3,085 | 71·0 |
| Macho-Aizpurua, 2020^191^ | 2004-15 | Spain | OC | CS | Conv | Patients with genital herpes | Culture | 1,003 | 74·3 |
| Magdaleno-Tapial, 2020^192^ | 2016-19 | Spain | Hospital | CS | Conv | Patients with genital herpes | PCR | 110 | 52·7 |
| Manavi, 2004^193^ | 1989-01 | UK | OC | CS | Conv | Patients with first-episode genital herpes | IF | 1,754 | 45·7 |
| Matondo, 1996^194^ | 1994-95 | UK | OC | CS | Conv | Women with first-episode genital herpes | IF | 121 | 48·8 |
| Melaugh, 2016^195^ | 2015-16 | Ireland | OC | CS | Conv | Patients with first-episode genital herpes | PCR | 36 | 16·7 |
| Nieuwenhuis, 2006^196^ | 1996-01 | Netherlands | OC | CS | Conv | Patients with first-episode genital herpes | MAb | 115 | 47·8 |
| Nieuwenhuis, 2006^196^ | 1996-01 | Netherlands | OC | CS | Conv | Patients with recurrent genital herpes | MAb | 419 | 83·1 |
| Nilsen, 2000^197^ | 1987-98 | Norway | OC | CS | Conv | Patients with first-episode genital herpes | MAb | 329 | 48·9 |
| Nilsen, 2000^197^ | 1987-98 | Norway | OC | CS | Conv | Patients with recurrent genital herpes | MAb | 498 | 87·8 |
| Nouchi, 2020^198^ | 2008-16 | France | OC | CS | Conv | Patients with first-episode genital herpes | PCR | 14 | 92·8 |
| Ooi, 2015^199^ | 2011-13 | UK | OC | CS | Conv | Patients with first-episode genital herpes | Culture | 743 | 52·0 |
| Parra-Sanchez, 2016^200^ | 2014-15 | Spain | OC | CS | Conv | STI clinic attendees with ulcers | PCR | 85 | 50·6 |
| Pérez-Torralba, 2020^176^ | 2018-19 | Spain | OC | CS | Conv | Patients with GUD | PCR | 125 | 24·8 |
| Peutherer, 1982^201^ | 1966-71 | UK | OC | CS | Conv | Patients with genital herpes | IF | 65 | 83·1 |
| Peutherer, 1982^201^ | 1977-79 | UK | OC | CS | Conv | Patients with genital herpes | IF | 150 | 76·7 |
| Ramaswamy, 2005^147^ | - | UK | OC | CS | Conv | Patients with genital herpes | PCR | 108 | 93·5 |
| Reina, 2005^202^ | 1995-03 | Spain | OC | CS | Conv | Patients with suspected genital herpes | MAb | 137 | 85·4 |
| Ross, 1993^203^ | 1978 | UK | OC | CS | Conv | Patients with genital herpes in 1978 | IF | 75 | 66·7 |
| Ross, 1993^203^ | 1979 | UK | OC | CS | Conv | Patients with genital herpes in 1979 | IF | 112 | 71·4 |
| Ross, 1993^203^ | 1980 | UK | OC | CS | Conv | Patients with genital herpes in 1980 | IF | 91 | 79·1 |
| Ross, 1993^203^ | 1981 | UK | OC | CS | Conv | Patients with genital herpes in 1981 | IF | 147 | 68·0 |
| Ross, 1993^203^ | 1982 | UK | OC | CS | Conv | Patients with genital herpes in 1982 | IF | 168 | 71·4 |
| Ross, 1993^203^ | 1983 | UK | OC | CS | Conv | Patients with genital herpes in 1983 | IF | 143 | 66·4 |
| Ross, 1993^203^ | 1984 | UK | OC | CS | Conv | Patients with genital herpes in 1984 | IF | 154 | 71·4 |
| Ross, 1993^203^ | 1985 | UK | OC | CS | Conv | Patients with genital herpes in 1985 | IF | 154 | 68·2 |
| Ross, 1993^203^ | 1986 | UK | OC | CS | Conv | Patients with genital herpes in 1986 | IF | 98 | 73·5 |
| Ross, 1993^203^ | 1987 | UK | OC | CS | Conv | Patients with genital herpes in 1987 | IF | 84 | 65·5 |
| Ross, 1993^203^ | 1988 | UK | OC | CS | Conv | Patients with genital herpes in 1988 | IF | 84 | 59·5 |
| Ross, 1993^203^ | 1989 | UK | OC | CS | Conv | Patients with genital herpes in 1989 | IF | 98 | 71·4 |
| Ross, 1993^203^ | 1990 | UK | OC | CS | Conv | Patients with genital herpes in 1990 | IF | 126 | 71·4 |
| Ross, 1993^203^ | 1991 | UK | OC | CS | Conv | Patients with genital herpes in 1991 | IF | 112 | 71·4 |
| Samra, 2003^204^ | 1993-02 | Israel | Hospital | CS | Conv | Patients with genital lesions | MAb | 285 | 56·8 |
| Sundaram, 2009^205^ | 2006-08 | UK | Hospital | CS | Conv | Patients with first-episode genital herpes | PCR | 547 | 53·9 |
| Scoular, 2002^206^ | 1986-88 | UK | OC | CS | Conv | Patients with genital herpes in Scotland | MAb | 564 | 66·8 |
| Scoular, 2002^206^ | 1989-91 | UK | OC | CS | Conv | Patients with genital herpes in 1989-91 | MAb | 339 | 62·0 |
| Scoular, 2002^206^ | 1992-94 | UK | OC | CS | Conv | Patients with genital herpes in 1992-94 | MAb | 593 | 50·3 |
| Scoular, 2002^206^ | 1995-97 | UK | OC | CS | Conv | Patients with genital herpes in 1995-97 | MAb | 653 | 43·2 |
| Scoular, 2002^206^ | 1998-00 | UK | OC | CS | Conv | Patients with genital herpes in 1998-00 | MAb | 977 | 43·9 |
| Slomka, 1998^178^ | - | UK | OC | CS | Conv | Patients with genital herpes | PCR | 108 | 65·7 |
| Strutt, 2003^207^ | 1997-98 | UK | OC | CS | Conv | Patients with genital herpes | IF | 334 | 85·9 |
| Tarin, 2021^208^ | 2019-20 | Spain | OC | CS | Conv | Patients with genital herpes | Other | 52 | 71·2 |
| Thompson, 2000^209^ | 1995-99 | UK | OC | CS | Conv | Patients with first-episode genital herpes | Culture | 105 | 28·6 |
| van de Laar, 1998^151^ | 1986-88 | Netherlands | OC | CS | Conv | Patients with first-episode genital herpes | Culture | 40 | 85·0 |
| van Oeffelen, 2018^210^ | - | Netherlands | OC | CS | Conv | Pregnant women | Culture | 10 | 70·0 |
| van Rooijen, 2016^152^ | 2000-11 | Netherlands | OC | Cohort^a^ | Conv | Patients with recurrent genital herpes | PCR | 85 | 77·7 |
| Wilson, 1994^211^ | - | UK | OC | CS | Conv | Patients with genital herpes attending STI clinic | Culture | 70 | 50·0 |
| Wolontis, 1977^212^ | - | Sweden | OC | CS | Conv | Patients with genital herpes | IO | 37 | 81·1 |
| Woolley, 1990^213^ | 1989 | UK | OC | CS | Conv | Patients with first-episode genital herpes | MAb | 129 | 58·9 |
| Woolley, 2000^153^ | - | UK | OC | CS | Conv | Patients with first-episode genital herpes | Culture | 13 | 30·8 |

^a^ The reported study design is the original study design. The included measures in this study are those for the baseline measures at the beginning of the study.

Abbreviations: Conv = Convenience, CS = Cross sectional, ELISA = Enzyme-linked immunosorbent type-specific assay, GE = Gel electrophoresis, GUD = Genital ulcer disease, GUM = Genitourinary medicine, HSV-2 = Herpes simplex virus type 2, IF = Immunofluorescence, IO = Immunoelectro osmophoresis, MAb = Monoclonal antibody, OC = Outpatient clinic, PCR = Polymerase chain reaction, STI = Sexually transmitted infections, UK = United Kingdom of Great Britain and Northern Ireland.

**Figure S2. Forest plots for the pooled mean proportions of HSV-2 virus detection in clinically diagnosed genital ulcer disease and in laboratory-confirmed genital herpes in Europe.**

1. **Patients with genital ulcer disease**


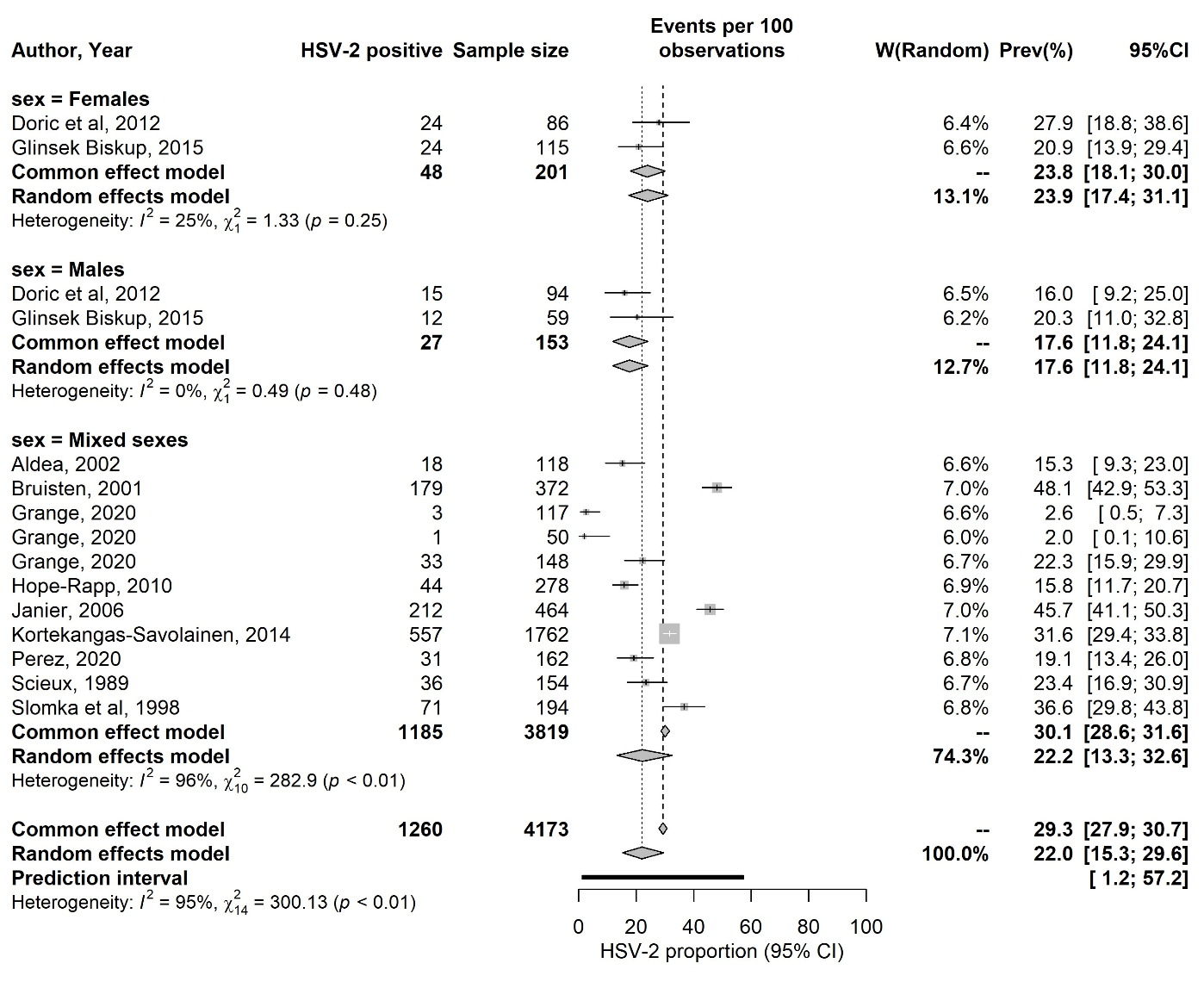


1. **Patients with genital herpes**

**
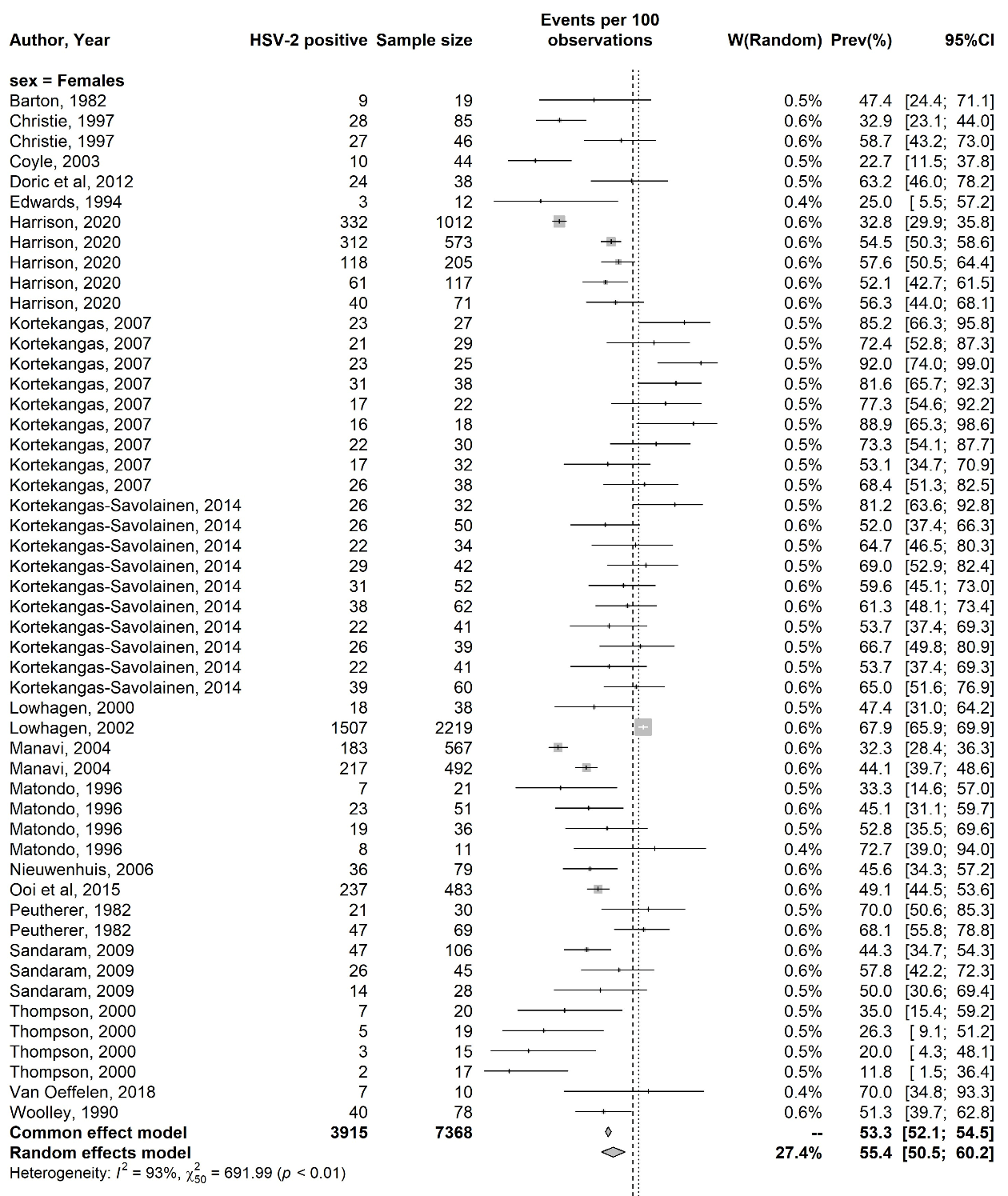
**

**
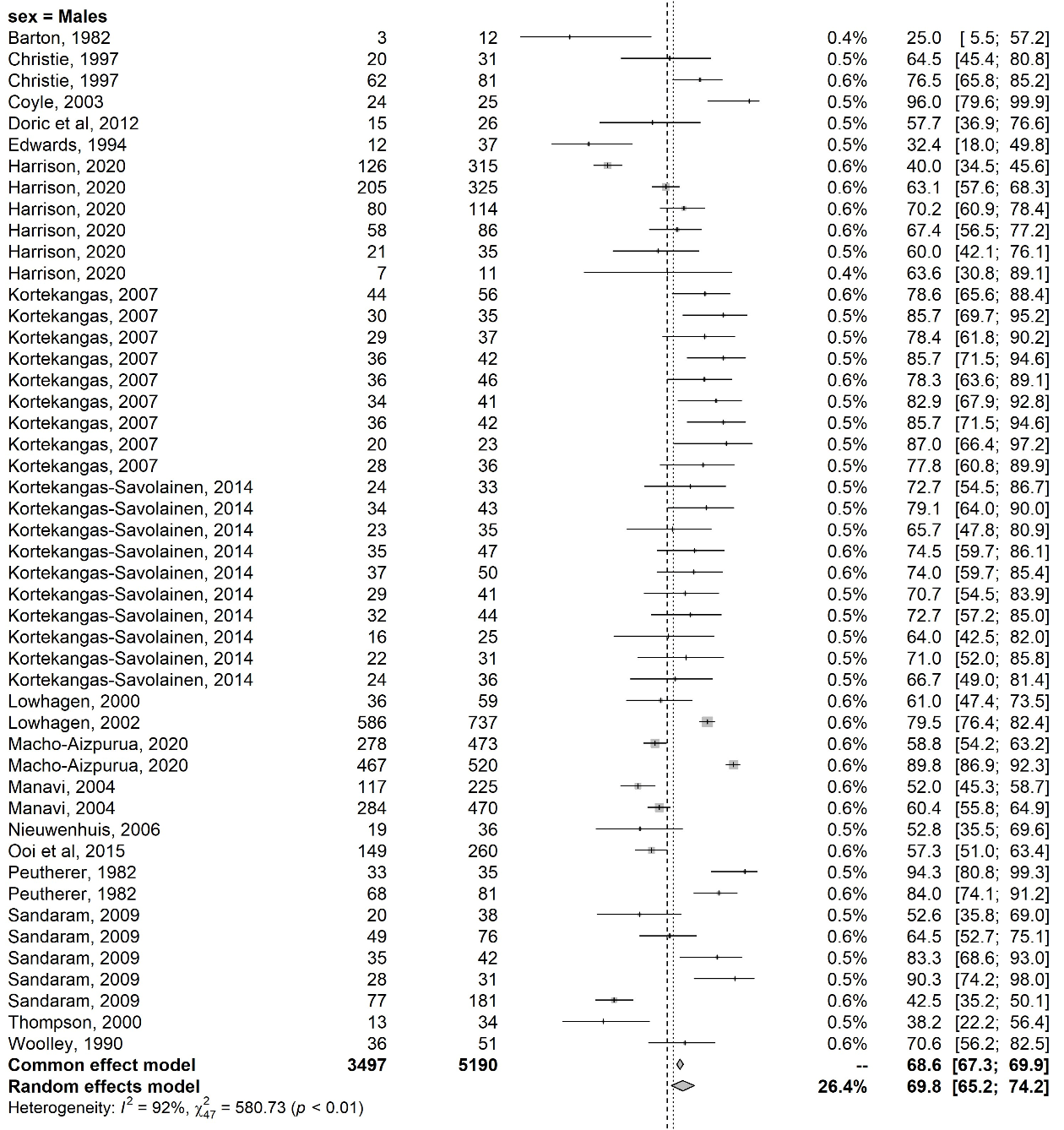
**

**
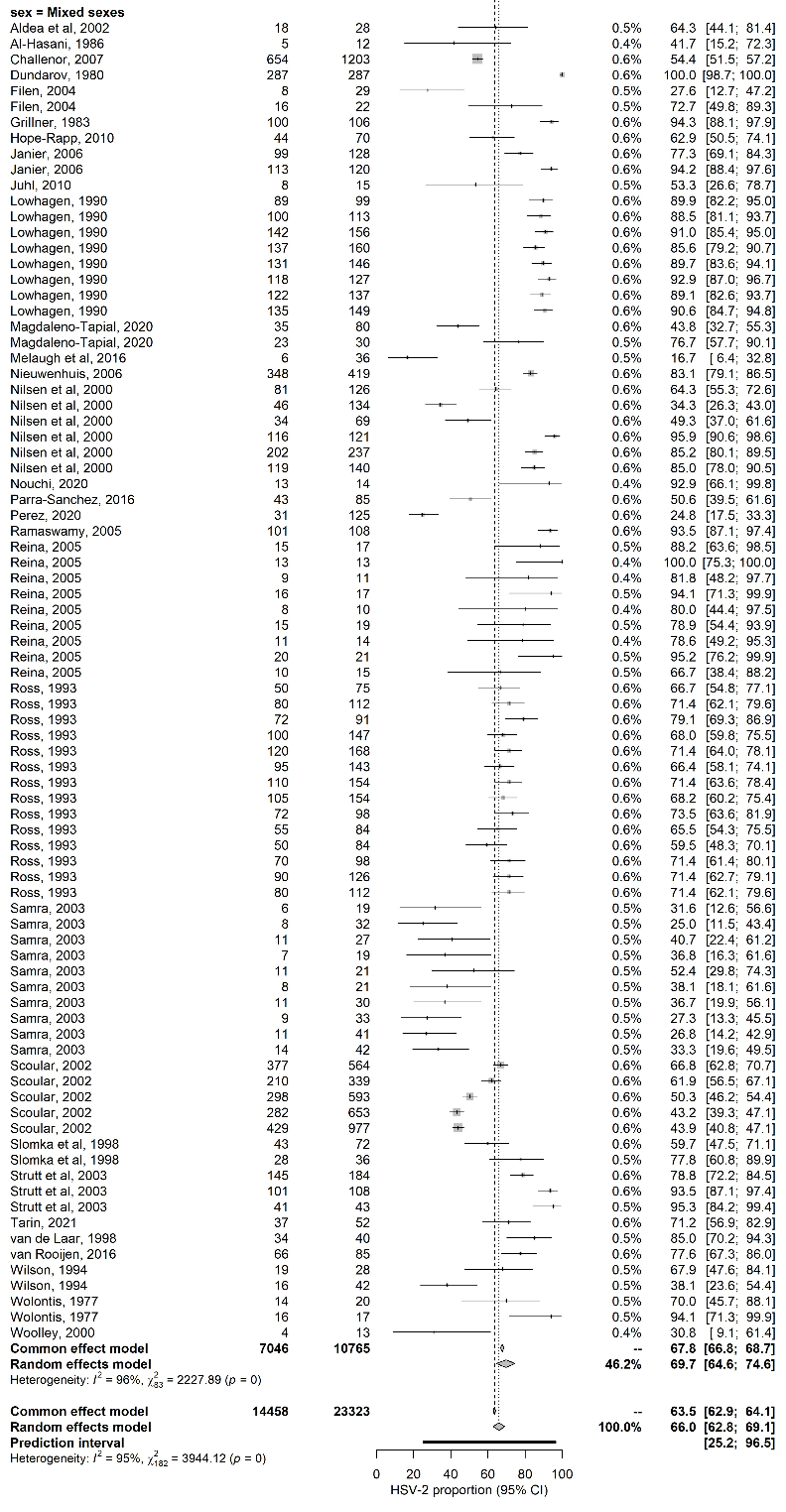
**

**Table S11. Univariable and multivariable meta-regression analyses for proportion of HSV-2 virus detection in laboratory-confirmed genital herpes in Europe, using the year of publication as the temporal variable instead of the year of data collection.**

|  | | **Outcome measures** | **Samples** | **Univariable analysis** | | | | **Multivariable analysis** | | | |  |
| --- | --- | --- | --- | --- | --- | --- | --- | --- | --- | --- | --- | --- |
|  |  |  |  |  |  |  |  | **Model 3^a^: Time as a categorical variable model** | | **Model 4^b^: Time as a linear variable model** | | |
|  | | **Total n** | **Total N** | ***RR* (95%CI)** | **p-value** | **LR test p-value^c^** | **Adjusted R^2^ (%)** | ***ARR* (95%CI)** | **p-value** | ***ARR* (95%CI)** | **p-value** | |
| **Age group** | <25 | 7 | 1638 | 1·00 | - | 0·005 | 10·09 | 1·00 | - | 1·00 | - |  |
|  | ≥25 | 15 | 1865 | 1·41 (1·05-1·88) | 0·021 |  |  | 1·51 (1·22-1·86) | <0·001 | 1·53 (1·24-1·89) | <0·001 |  |
|  | Mixed | 161 | 19,820 | 1·50 (1·18-1·93) | 0·001 |  |  | 1·23 (1·01-1·49) | 0·038 | 1·21 (1·00-1·46) | 0·051 |  |
| **Sex** | Women | 51 | 7,368 | 1·00 | - | 0·001 | 10·39 | 1·00 | - | 1·00 | - |  |
|  | Men | 48 | 5,190 | 1·23 (1·08-1·39) | 0·001 |  |  | 1·21 (1·10-1·33) | <0·001 | 1·21 (1·11-1·32) | <0·001 |  |
|  | Mixed | 84 | 10,765 | 1·21 (1·08-1·35) | 0·001 |  |  | 1·07 (0·97-1·19) | 0·169 | 1·07 (0·97-1·18) | 0·199 |  |
| **Genital herpes episode status** | First episode | 57 | 9,135 | 1·00 | - | <0·001 | 27·34 | 1·00 | - | 1·00 | - |  |
|  | Recurrent episode | 13 | 1,907 | 1·54 (1·30-1·82) | <0·001 |  |  | 1·55 (1·34-1·79) | <0·001 | 1·53 (1·33-1·77) | <0·001 |  |
|  | Unspecified status | 113 | 12,281 | 1·31 (1·19-1·44) | <0·001 |  |  | 1·36 (1·24-1·50) | <0·001 | 1·35 (1·23-1·48) | <0·001 |  |
| **European subregion/ Country** | Southern Europe^d^ | 20 | 1,881 | 1·00 | - | <0·001 | 13·30 | 1·00 | - | 1·00 | - |  |
|  | Western Europe | 11 | 1,016 | 1·01 (0·81-1·26) | 0·909 |  |  | 0·10 (0·92-1·30) | 0·298 | 1·11 (0·94-1·32) | 0·211 |  |
|  | Northern Europe | 142 | 20,141 | 0·91 (0·79-1·05) | 0·185 |  |  | 0·90 (0·80-1·02) | 0·089 | 0·89 (0·79-1·00) | 0·053 |  |
|  | Israel | 10 | 285 | 0·49 (0·37-0·64) | <0·001 |  |  | 0·45 (0·35-0·57) | <0·001 | 0·44 (0·35-0·55) | <0·001 |  |
| **Sample size^e^** | <200 | 49 | 2032 | 1·00 | - | 0·014 | 0·53 | 1·00 | - | 1·00 | - |  |
|  | ≥200 | 134 | 21,291 | 1·15 (1·03-1·29) | 0·014 |  |  | 1·14 (1·04-1·25) | 0·006 | 1·15 (1·05-1·26) | 0·002 |  |
| **Year of publication category** | <2000 | 50 | 4,179 | 1·00 | - | 0·106 | 3·56 | 1·00 | - | - | - |  |
|  | 2000-2010 | 87 | 13,125 | 0·91 (0·81-1·02) | 0·111 |  |  | 0·89 (0·81-0·99) | 0·026 | - | - |  |
|  | >2010 | 46 | 6,019 | 0·87 (0·77-0·99) | 0·042 |  |  | 0·81 (0·72-0·91) | 0·001 | - | - |  |
| **Year of publication** | | 183 | 23,323 | 0·99 (0·99-1·00) | 0·032 | 0·032 | 5·59 | - | - | 0·99 (0·99-1·00) | <0·001 | |

^a^ Variance explained by the final multivariable model 3 (adjusted *R^2^*) = 55·71%.

^b^ Variance explained by the final multivariable model 4 (adjusted *R^2^*) = 57·42%.

^c^ Factors in the univariable analyses with a p-value<0.1 were included in the multivariable analysis.

^d^ Southern Europe includes one measure from Eastern Europe.

^e^ Sample size denotes the sample size of the study population found in the original publication.

Abbreviations: ARR = Adjusted risk ratio, CI = Confidence interval, HSV-2 = Herpes simplex virus type 2, LR = Likelihood ratio, RR = Risk ratio.

**
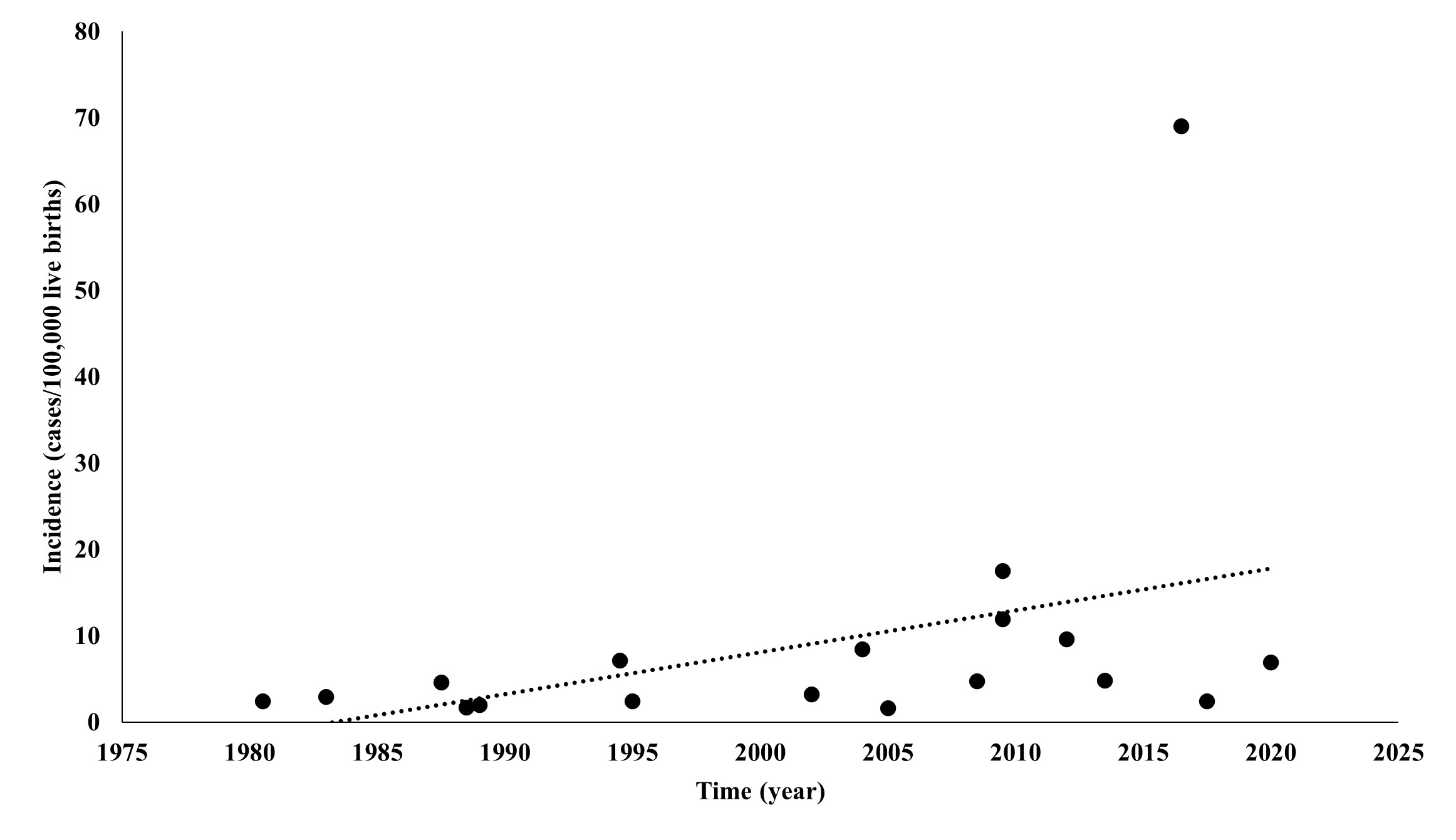
**

**Figure S3. Trend of neonatal herpes incidence in Europe.**

**References**

1. Page MJ, McKenzie JE, Bossuyt PM, et al. The PRISMA 2020 statement: an updated guideline for reporting systematic reviews. *BMJ* 2021; **372**: n71.

2. Arvaja M, Lehtinen M, Koskela P, Lappalainen M, Paavonen J, Vesikari T. Serological evaluation of herpes simplex virus type 1 and type 2 infections in pregnancy. *Sexually Transmitted Infections* 1999; **75**(3): 168-71.

3. Eskild A, Jeansson S, Jenum PA. [Antibodies against Herpes simplex virus type 2 among pregnant women in Norway]. *Tidsskr Nor Laegeforen* 1999; **119**(16): 2323-6.

4. Suligoi B, Torri A, Grilli G, Tanzi E, Palu G. Seroprevalence and seroincidence of herpes simplex virus type 1 and herpes simplex virus type 2 infections in a cohort of adolescents in Italy. *Sex Transm Dis* 2004; **31**(10): 608-10.

5. Davidovici BB, Grotto I, Balicer RD, Robinson NJ, Cohen D. Decline in the prevalence of antibodies to herpes simplex virus types 1 and 2 among Israeli young adults between 1984 and 2002. *Sex Transm Dis* 2006; **33**(11): 641-5.

6. Keet I, Lee FK, van Griensven G, Lange J, Nahmias A, Coutinho R. Herpes simplex virus type 2 and other genital ulcerative infections as a risk factor for HIV-1 acquisition. *Sexually Transmitted Infections* 1990; **66**(5): 330-3.

7. Varela JA, Garcia-Corbeira P, Aguanell MV, et al. Herpes simplex virus type 2 seroepidemiology in Spain: prevalence and seroconversion rate among sexually transmitted disease clinic attendees. *Sex Transm Dis* 2001; **28**(1): 47-50.

8. Ades AE, Peckham CS, Dale GE, Best JM, Jeansson S. Prevalence of antibodies to herpes simplex virus types 1 and 2 in pregnant women, and estimated rates of infection. *Journal of Epidemiology and Community Health* 1989; **43**(1): 53-60.

9. Alanen A, Kahala K, Vahlberg T, Koskela P, Vainionpaa R. Seroprevalence, incidence of prenatal infections and reliability of maternal history of varicella zoster virus, cytomegalovirus, herpes simplex virus and parvovirus B19 infection in South-Western Finland. *Bjog* 2005; **112**(1): 50-6.

10. Andersson-Ellstrom A, Svennerholm B, Forssman L. Prevalence of antibodies to herpes simplex virus types 1 and 2, Epstein-Barr virus and cytomegalovirus in teenage girls. *Scand J Infect Dis* 1995; **27**(4): 315-8.

11. Arama V, Cercel AS, Vladareanu R, et al. Type-specific herpes simplex virus-1 and herpes simplex virus-2 seroprevalence in Romania: comparison of prevalence and risk factors in women and men. *International journal of infectious diseases: IJID: official publication of the International Society for Infectious Diseases* 2010; **14 Suppl 3**: e25-31.

12. Arnheim Dahlstrom L, Andersson K, Luostarinen T, et al. Prospective seroepidemiologic study of human papillomavirus and other risk factors in cervical cancer. *Cancer Epidemiol Biomarkers Prev* 2011; **20**(12): 2541-50.

13. Balaeva T, Grjibovski AM, Sidorenkov O, et al. Seroprevalence and correlates of herpes simplex virus type 2 infection among young adults in Arkhangelsk, Northwest Russia: a population-based cross-sectional study. *BMC Infect Dis* 2016; **16**(1): 616.

14. Benharrosh J, Dauphin H, Porcheret H, Meritet JF, Boulanger MC, Maisonneuve L. [Comparison of two ELISA tests to study the seroprevalence of herpes simplex 1 et 2 infection in a maternity near Paris]. *Ann Biol Clin (Paris)* 2008; **66**(6): 665-70.

15. Berntsson M, Tunback P, Ellstrom A, Krantz I, Lowhagen GB. Decreasing prevalence of herpes simplex virus-2 antibodies in selected groups of women in Sweden. *Acta Derm Venereol* 2009; **89**(6): 623-6.

16. Bjerke SEY, Holter E, Vangen S, Stray-Pedersen B. Sexually transmitted infections among Pakistani pregnant women and their husbands in Norway. *International Journal of Women's Health* 2010; **2**(1): 303-9.

17. Blomstrom A, Karlsson H, Wicks S, Yang S, Yolken RH, Dalman C. Maternal antibodies to infectious agents and risk for non-affective psychoses in the offspring-a matched case-control study. *Schizophrenia Research* 2012; **140**(1-3): 25-30.

18. Bodeus M, Laffineur K, Kabamba-Mukadi B, Hubinont C, Bernard P, Goubau P. Seroepidemiology of Herpes Simplex Type 2 in Pregnant Women in Belgium. *Sexually Transmitted Diseases* 2004; **31**(5): 297-300.

19. Bunzli D, Wietlisbach V, Barazzoni F, Sahli R, Meylan PR. Seroepidemiology of Herpes Simplex virus type 1 and 2 in Western and Southern Switzerland in adults aged 25-74 in 1992-93: a population-based study. *BMC Infect Dis* 2004; **4**: 10.

20. Bystricka M, Solarikova L, Gasparikova L, et al. Antibody responses to the herpes simplex virus type 2 glycoprotein G in sera of human immunodeficiency virus-infected patients in Slovakia. *Acta Virol* 1998; **42**(5): 319-24.

21. Canessa A, Pantarotto F, Miletich F, et al. Antibody prevalence to torch agents in pregnant women and relative risk of congenital infections in Italy (Liguria). *Biol Res Pregnancy Perinatol* 1987; **8**(2 2D Half): 84-8.

22. Celentano DD, Mayer KH, Pequegnat W, et al. Prevalence of Sexually Transmitted Diseases and Risk Behaviors from the NIMH Collaborative HIV/STD Prevention Trial. *International journal of sexual health : official journal of the World Association for Sexual Health* 2010; **22**(4): 272-84.

23. Cheslack-Postava K, Brown AS, Chudal R, et al. Maternal exposure to sexually transmitted infections and schizophrenia among offspring. *Schizophr Res* 2015; **166**(1-3): 255-60.

24. Christenson B, Bottiger M, Svensson A, Jeansson S. A 15-year surveillance study of antibodies to herpes simplex virus types 1 and 2 in a cohort of young girls. *The Journal of infection* 1992; **25**(2): 147-54.

25. Cliff JM, King EC, Lee JS, et al. Cellular Immune Function in Myalgic Encephalomyelitis/Chronic Fatigue Syndrome (ME/CFS). *Front Immunol* 2019; **10**: 796.

26. Cowan FM, French RS, Mayaud P, et al. Seroepidemiological study of herpes simplex virus types 1 and 2 in Brazil, Estonia, India, Morocco, and Sri Lanka. *Sexually Transmitted Infections* 2003; **79**(4): 286-90.

27. Dan M, Sadan O, Glezerman M, Raveh D, Samra Z. Prevalence and risk factors for herpes simplex virus type 2 infection among pregnant women in Israel. *Sex Transm Dis* 2003; **30**(11): 835-8.

28. Davidovici BB, Green M, Marouni MJ, Bassal R, Pimenta JM, Cohen D. Seroprevalence of herpes simplex virus 1 and 2 and correlates of infection in Israel. *The Journal of infection* 2006; **52**(5): 367-73.

29. De Ory F, Pachon I, Echevarria JM, Ramirez R. Seroepidemiological study of herpes simplex virus in the female population in the autonomous region of Madrid, Spain. *European Journal of Clinical Microbiology and Infectious Diseases* 1999; **18**(9): 678-80.

30. De Ory F, Echevarria JM, Pachon I, Ramirez R. Seroprevalence of type 2 herpes simplex virus in an adult population in the community of Madrid. *Enfermedades infecciosas y microbiologia clinica* 2000; **18**(8): 420-1.

31. De Sanjose S, Munoz N, Bosch FX, et al. Sexually transmitted agents and cervical neoplasia in Colombia and Spain. *International Journal of Cancer* 1994; **56**(3): 358-63.

32. Dolar N, Serdaroglu S, Yilmaz G, Ergin S. Seroprevalence of herpes simplex virus type 1 and type 2 in Turkey. *J Eur Acad Dermatol Venereol* 2006; **20**(10): 1232-6.

33. Dordevic H. Serological response to herpes simplex virus type 1 and 2 infection among women of reproductive age. *Medicinski pregled* 2006; **59**(11-12): 591-7.

34. Eis-Hübinger AM, Däumer M, Matz B, Schneweis KE. Evaluation of three glycoprotein G2-based enzyme immunoassays for detection of antibodies to herpes simplex virus type 2 in human sera. *Journal of Clinical Microbiology* 1999; **37**(5): 1242-6.

35. Enders G, Risse B, Zauke M, Bolley I, Knotek F. Seroprevalence study of herpes simplex virus type 2 among pregnant women in Germany using a type-specific enzyme immunoassay. *Eur J Clin Microbiol Infect Dis* 1998; **17**(12): 870-2.

36. Esteban-Hernandez J, San Roman Montero J, Gil R, Anegon M, Gil A. Association between herpetic burden and chronic ischemic heart disease: Matched case-control study. *Medicina Clinica* 2011; **137**(4): 157-60.

37. Eskild A, Jeansson S, Hagen JA, Jenum PA, Skrondal A. Herpes simplex virus type-2 antibodies in pregnant women: the impact of the stage of pregnancy. *Epidemiol Infect* 2000; **125**(3): 685-92.

38. Eskild A, Jeansson S, Stray-Pedersen B, Jenum PA. Herpes simplex virus type-2 infection in pregnancy: no risk of fetal death: results from a nested case-control study within 35,940 women. *Bjog* 2002; **109**(9): 1030-5.

39. Espinola-Klein C, Rupprecht HJ, Blankenberg S, et al. Impact of infectious burden on progression of carotid atherosclerosis. *Stroke* 2002; **33**(11): 2581-6.

40. Forbes H, Warne B, Doelken L, et al. Risk factors for herpes simplex virus type-1 infection and reactivation: Cross-sectional studies among EPIC-Norfolk participants. *PLoS One* 2019; **14**(5): e0215553.

41. Forsgren M, Skoog E, Jeansson S, Olofsson S, Giesecke J. Prevalence of antibodies to herpes simplex virus in pregnant women in Stockholm in 1969, 1983 and 1989: implications for STD epidemiology. *Int J STD AIDS* 1994; **5**(2): 113-6.

42. Garcia-Corbeira P, Hogrefe W, Aguilar L, et al. Whole cell lysate enzyme immunoassays vs. Recombinant glycoprotein G2- based immunoassays for HSV-2 seroprevalence studies. *Journal of Medical Virology* 1999; **59**(4): 502-6.

43. Garcia-Corbeira P, Dal-Re R, Aguilar L, Granizo JJ, Garcia-de-Lomas J. Is sexual transmission an important pattern for herpes simplex type 2 virus seroconversion in the Spanish general population? *J Med Virol* 1999; **59**(2): 194-7.

44. Gaytant MA, Steegers EA, van Laere M, et al. Seroprevalences of herpes simplex virus type 1 and type 2 among pregnant women in the Netherlands. *Sex Transm Dis* 2002; **29**(11): 710-4.

45. Gorander S, Lagergard T, Romanik M, Viscidi RP, Martirosian G, Liljeqvist JA. Seroprevalences of herpes simplex virus type 2, five oncogenic human papillomaviruses, and Chlamydia trachomatis in Katowice, Poland. *Clinical and vaccine immunology : CVI* 2008; **15**(4): 675-80.

46. Hamdani N, Daban-Huard C, Godin O, et al. Effects of Cumulative Herpesviridae and Toxoplasma gondii Infections on Cognitive Function in Healthy, Bipolar, and Schizophrenia Subjects. *The Journal of clinical psychiatry* 2017; **78**(1): e18-e27.

47. Hawkes CH, Giovannoni G, Keir G, Cunnington M, Thompson EJ. Seroprevalence of herpes simplex virus type 2 in multiple sclerosis. *Acta neurologica Scandinavica* 2006; **114**(6): 363-7.

48. Hellenbrand W, Thierfelder W, Muller-Pebody B, Hamouda O, Breuer T. Seroprevalence of herpes simplex virus type 1 (HSV-1) and type 2 (HSV-2) in former East and West Germany, 1997-1998. *Eur J Clin Microbiol Infect Dis* 2005; **24**(2): 131-5.

49. Hettmann A, Gerle B, Barcsay E, Csiszar C, Takacs M. Seroprevalence of HSV-2 in Hungary and comparison of the HSV-2 prevalence of pregnant and infertile women. *Acta microbiologica et immunologica Hungarica* 2008; **55**(4): 429-36.

50. Isacsohn M, Smetana Z, Rones ZZ, et al. A sero-epidemiological study of herpes virus type 1 and 2 infection in Israel. *J Clin Virol* 2002; **24**(1-2): 85-92.

51. Jha PKS, Beral V, Peto J, et al. Antibodies to human papillomavirus and to other genital infectious agents and invasive cervical cancer risk. *Lancet* 1993; **341**(8853): 1116-8.

52. Jonsson M, Karlsson R, Rylander E, et al. The silent suffering women - A population based study on the association between reported symptoms and past and present infections of the lower genital tract. *Genitourinary Medicine* 1995; **71**(3): 158-62.

53. Jonsson MK, Levi M, Ruden U, Wahren B. Minimal change in HSV-2 seroreactivity: a cross-sectional Swedish population study. *Scand J Infect Dis* 2006; **38**(5): 357-65.

54. Juhl D, Mosel C, Nawroth F, et al. Detection of herpes simplex virus DNA in plasma of patients with primary but not with recurrent infection: implications for transfusion medicine? *Transfusion medicine (Oxford, England)* 2010; **20**(1): 38-47.

55. Karachaliou M, Chatzi L, Roumeliotaki T, et al. Common infections with polyomaviruses and herpesviruses and neuropsychological development at 4 years of age, the Rhea birth cohort in Crete, Greece. *Journal of child psychology and psychiatry, and allied disciplines* 2016; **57**(11): 1268-76.

56. Karachaliou M, Waterboer T, Casabonne D, et al. The natural history of human polyomaviruses and herpesviruses in early life - The rhea birth cohort in Greece. *American Journal of Epidemiology* 2016; **183**(7): 671-9.

57. Karaer A, Mert I, Cavkaytar S, Batioglu S. Serological investigation of the role of selected sexually transmitted infections in the aetiology of ectopic pregnancy. *The European journal of contraception & reproductive health care : the official journal of the European Society of Contraception* 2013; **18**(1): 68-74.

58. Khryanin AA, Reshetnikov OV. Seroprevalence of herpes simplex virus type 2 infection in Russia. *Int J STD AIDS* 2007; **18**(11): 797-8.

59. Kibur M, Koskela P, Dillner J, et al. Seropositivity to multiple sexually transmitted infections is not common. *Sex Transm Dis* 2000; **27**(8): 425-30.

60. Korodi Z, Wang X, Tedeschi R, Knekt P, Dillner J. No serological evidence of association between prostate cancer and infection with herpes simplex virus type 2 or human herpesvirus type 8: a nested case-control study. *J Infect Dis* 2005; **191**(12): 2008-11.

61. Korr G, Thamm M, Czogiel I, Poethko-Mueller C, Bremer V, Jansen K. Decreasing seroprevalence of herpes simplex virus type 1 and type 2 in Germany leaves many people susceptible to genital infection: time to raise awareness and enhance control. *BMC Infect Dis* 2017; **17**(1): 471.

62. Kramer MA, Uitenbroek DG, Ujcic-Voortman JK, et al. Ethnic differences in HSV1 and HSV2 seroprevalence in Amsterdam, the Netherlands. *Euro surveillance : bulletin Europeen sur les maladies transmissibles = European communicable disease bulletin* 2008; **13**(24).

63. Krone B, Pohl D, Rostasy K, et al. Common infectious agents in multiple sclerosis: a case-control study in children. *Multiple sclerosis (Houndmills, Basingstoke, England)* 2008; **14**(1): 136-9.

64. Kucera P, Gerber S, Marques-Vidal P, Meylan PR. Seroepidemiology of herpes simplex virus type 1 and 2 in pregnant women in Switzerland: an obstetric clinic based study. *Eur J Obstet Gynecol Reprod Biol* 2012; **160**(1): 13-7.

65. Laubereau B, Zwahlen M, Neuenschwander B, Heininger U, Schaad UB, Desgrandchamps D. [Herpes simplex virus type 1 and 2 in Switzerland]. *Schweiz Med Wochenschr* 2000; **130**(5): 143-50.

66. LeGoff J, Saussereau E, Boulanger MC, et al. Unexpected high prevalence of herpes simplex virus (HSV) type 2 seropositivity and HSV genital shedding in pregnant women living in an East Paris suburban area. *Int J STD AIDS* 2007; **18**(9): 593-5.

67. Lehtinen M, Dillner J, Knekt P, et al. Serologically diagnosed infection with human papillomavirus type 16 and risk for subsequent development of cervical carcinoma: Nested case-control study. *British Medical Journal* 1996; **312**(7030): 537-9.

68. Lorber N. Comparison of infection of pregnant women with Herpes simplex viruses types 1 and 2 in Slovenia at ten-year intervals. Diploma work University of Ljubljana, Biotechnical Faculty, Interdepartmental Study Unit of Microbiology; 2006.

69. Lowhagen GB, Jansen E, Nordenfelt E, Lycke E. Epidemiology of genital herpes infections in Sweden. *Acta Derm Venereol* 1990; **70**(4): 330-4.

70. Mahic M, Mjaaland S, Bovelstad HM, et al. Maternal immunoreactivity to herpes simplex virus 2 and risk of autism spectrum disorder in male offspring. *mSphere* 2017; **2**(1).

71. Malkin JE, Morand P, Malvy D, et al. Seroprevalence of HSV-1 and HSV-2 infection in the general French population. *Sex Transm Infect* 2002; **78**(3): 201-3.

72. Maral I, Biri A, Korucuoglu U, Bakar C, Cirak M, Ali Bumin M. Seroprevalences of herpes simplex virus type 2 and Chlamydia trachomatis in Turkey. *Archives of gynecology and obstetrics* 2009; **280**(5): 739-43.

73. Marchesi S, Lupattelli G, Sensini A, et al. Racial difference in endothelial function: Role of the infective burden. *Atherosclerosis* 2007; **191**(1): 227-34.

74. Marchi S, Trombetta CM, Gasparini R, Temperton N, Montomoli E. Epidemiology of herpes simplex virus type 1 and 2 in Italy: a seroprevalence study from 2000 to 2014. *Journal of preventive medicine and hygiene* 2017; **58**(1): E27-e33.

75. Miskulin M, Miskulin I, Milas J, Antolovic-Pozgain A, Rudan S, Vuksic M. Prevalence and risk factors for herpes simplex virus type 2 infections in East Croatia. *Collegium antropologicum* 2011; **35**(1): 9-14.

76. Morris-Cunnington M, Brown D, Pimenta J, Robinson NJ, Miller E. New estimates of herpes simplex virus type 2 seroprevalence in England: 'high' but stable seroprevalence over the last decade. *Sex Transm Dis* 2004; **31**(4): 243-6.

77. Munoz N, Kato I, Bosch FX, et al. Cervical cancer and Herpes Simplex Virus type 2: Case-control studies in spain and colombia, with special reference to immunoglobulin-G sub-classes. *International Journal of Cancer* 1995; **60**(4): 438-42.

78. Nilsen A, Ulvestad E, Marsden H, et al. Performance characteristics of a glycoprotein G based oligopeptide (peptide 55) and two different methods using the complete glycoprotein as assays for detection of anti-HSV-2 antibodies in human sera. *Journal of Virological Methods* 2003; **107**(1): 21-7.

79. Nilsen A, Mwakagile D, Marsden H, Langeland N, Matre R, Haarr L. Prevalence of, and risk factors for, HSV-2 antibodies in sexually transmitted disease patients, healthy pregnant females, blood donors and medical students in Tanzania and Norway. *Epidemiol Infect* 2005; **133**(5): 915-25.

80. NIMH group. Sexually transmitted disease and HIV prevalence and risk factors in concentrated and generalized HIV epidemic settings. *Aids* 2007; **21 Suppl 2**: S81-90.

81. Ohana B, Lipson M, Vered N, Srugo I, Ahdut M, Morag A. Novel approach for specific detection of herpes simplex virus type 1 and 2 antibodies and immunoglobulin G and M antibodies. *Clin Diagn Lab Immunol* 2000; **7**(6): 904-8.

82. Olsson J, Kok E, Adolfsson R, Lovheim H, Elgh F. Herpes virus seroepidemiology in the adult Swedish population. *Immunity and Ageing* 2017; **14**(1): 10.

83. Opaneye AA, Bashford J. Seroprevalence of antibodies to herpes simplex virus types 1 and 2 among two sexually active female populations in Middlesbrough, England. *The journal of the Royal Society for the Promotion of Health* 2002; **122**(2): 108-11.

84. Ozdemir R, Hakan ER, Baran N, Vural A, Demircl M. HSV-1 and HSV-2 seropositivity rates in pregnant women admitted to Izmir Ataturk Research and Training Hospital, Turkey. *Mikrobiyoloji Bulteni* 2009; **43**(4): 709-11.

85. Papadogeorgakis H, Caroni C, Katsambas A, et al. Herpes simplex virus seroprevalence among children, adolescents and adults in Greece. *Int J STD AIDS* 2008; **19**(4): 272-8.

86. Pasquini P, Mele A, Franco E, Ippolito G, Svennerholm B. Prevalence of herpes simplex virus type 2 antibodies in selected population groups in Italy. *Eur J Clin Microbiol Infect Dis* 1988; **7**(1): 54-6.

87. Pebody RG, Andrews N, Brown D, et al. The seroepidemiology of herpes simplex virus type 1 and 2 in Europe. *Sex Transm Infect* 2004; **80**(3): 185-91.

88. Persson K, Mansson A, Jonsson E, Nordenfelt E. Decline of herpes simplex virus type 2 and Chlamydia trachomatis infections from 1970 to 1993 indicated by a similar change in antibody pattern. *Scand J Infect Dis* 1995; **27**(3): 195-9.

89. Petersen CS, Larsen FG, Zachariae C, Heidenheim M. Herpes simplex virus-type 2 seropositivity in a Danish adult population denying previous episodes of genital herpes. *Acta Derm Venereol* 2000; **80**(2): 158.

90. Puhakka L, Sarvikivi E, Lappalainen M, Surcel HM, Saxen H. Decrease in seroprevalence for herpesviruses among pregnant women in Finland: cross-sectional study of three time points 1992, 2002 and 2012. *Infectious diseases (London, England)* 2016; **48**(5): 406-10.

91. Rabenau H, Buxbaum S, Preiser W, Weber B, Doerr H. Seroprevalence of herpes simplex virus types 1 and type 2 in the Frankfurt am Main area, Germany. *Medical Microbiology and Immunology* 2002; **190**(4): 153-60.

92. Rode OD, Lepej SZ, Begovac J. Seroprevalence of herpes simplex virus type 2 in adult HIV-infected patients and blood donors in Croatia. *Collegium antropologicum* 2008; **32**(3): 693-5.

93. Sauerbrei A, Schmitt S, Scheper T, et al. Seroprevalence of herpes simplex virus type 1 and type 2 in Thuringia, Germany, 1999 to 2006. *Euro surveillance : bulletin Europeen sur les maladies transmissibles = European communicable disease bulletin* 2011; **16**(44).

94. Shev S, Hermodsson S, Lindholm A, Malm E, Widell A, Norkrans G. Risk factor exposure among hepatitis C virus RNA positive Swedish blood donors--the role of parenteral and sexual transmission. *Scand J Infect Dis* 1995; **27**(2): 99-104.

95. Silins I, Kallings I, Dillner J. Correlates of the spread of human papillomavirus infection. *Cancer Epidemiol Biomarkers Prev* 2000; **9**(9): 953-9.

96. Slomka MJ, Ashley RL, Cowan FM, Cross A, Brown DW. Monoclonal antibody blocking tests for the detection of HSV-1- and HSV-2-specific humoral responses: comparison with western blot assay. *J Virol Methods* 1995; **55**(1): 27-35.

97. Smith JS, Rosinska M, Trzcinska A, Pimenta JM, Litwinska B, Siennicka J. Type specific seroprevalence of HSV-1 and HSV-2 in four geographical regions of Poland. *Sexually Transmitted Infections* 2006; **82**(2): 159-63.

98. Snijders G, van Mierlo HC, Boks MP, et al. The association between antibodies to neurotropic pathogens and bipolar disorder : A study in the Dutch Bipolar (DB) Cohort and meta-analysis. *Transl Psychiatry* 2019; **9**(1): 311.

99. Stock C, Guillén-Grima F, de Mendoza JH, Marin-Fernandez B, Aguinaga-Ontoso I, Krämer A. Risk factors of herpes simplex type 1 (HSV-1) infection and lifestyle factors associated with HSV-1 manifestations. *Eur J Epidemiol* 2001; **17**(9): 885-90.

100. Strandberg TE, Pitkala KH, Linnavuori KH, Tilvis RS. Impact of viral and bacterial burden on cognitive impairment in elderly persons with cardiovascular diseases. *Stroke* 2003; **34**(9): 2126-31.

101. Suligoi B, Cusan M, Santopadre P, et al. HSV-2 specific seroprevalence among various populations in Rome, Italy. *Sexually Transmitted Infections* 2000; **76**(3): 213-4.

102. Suligoi B, Quaglio G, Regine V, et al. Seroprevalence of HIV, HSV-2, and Treponema pallidum in the Kosovarian population. *Scand J Infect Dis* 2009; **41**(8): 608-13.

103. Topbas M, Can E, Kaklikkaya N, Yavuzyilmaz A, Ozkan G, Can G. Herpes simplex virus type-2 seroprevalence among adults aged 20-49 in Trabzon. *Nobel Medicus* 2012; **8**(2): 85-90.

104. Tunback P, Bergstrom T, Andersson A, Nordin P, Krantz I, Lowhagen GB. Prevalence of herpes simplex virus antibodies in childhood and adolescence: A cross-sectional study. *Scandinavian Journal of Infectious Diseases* 2003; **35**(8): 498-502.

105. Vass-Sorensen M, Abeler V, Berle E, et al. Prevalence of antibodies to herpes simplex virus and frequency of HLA antigens in patients with preinvasive and invasive cervical cancer. *Gynecologic oncology* 1984; **18**(3): 349-58.

106. Vilibić-Čavlek T, Kolarić B, Bogdanić M, Tabain I, Beader N. Herpes Group Viruses: a Seroprevalence Study in Hemodialysis Patients. *Acta Clin Croat* 2017; **56**(2): 255-61.

107. Vilibic-Cavlek T, Ljubin-Sternak S, Ban M, Kolaric B, Sviben M, Mlinaric-Galinovic G. Seroprevalence of TORCH infections in women of childbearing age in Croatia. *The journal of maternal-fetal & neonatal medicine : the official journal of the European Association of Perinatal Medicine, the Federation of Asia and Oceania Perinatal Societies, the International Society of Perinatal Obstet* 2011; **24**(2): 280-3.

108. Vilibic-Cavlek T, Kolaric B, Ljubin-Sternak S, Mlinaric-Galinovic G. Herpes simplex virus infection in the Croatian population. *Scand J Infect Dis* 2011; **43**(11-12): 918-22.

109. Vyse AJ, Gay NJ, Slomka MJ, et al. The burden of infection with HSV-1 and HSV-2 in England and Wales: implications for the changing epidemiology of genital herpes. *Sex Transm Infect* 2000; **76**(3): 183-7.

110. Wang H, Yolken RH, Hoekstra PJ, Burger H, Klein HC. Antibodies to infectious agents and the positive symptom dimension of subclinical psychosis: The TRAILS study. *Schizophr Res* 2011; **129**(1): 47-51.

111. Warnecke JM, Pollmann M, Borchardt-Loholter V, et al. Seroprevalences of antibodies against ToRCH infectious pathogens in women of childbearing age residing in Brazil, Mexico, Germany, Poland, Turkey and China. *Epidemiology and Infection* 2020.

112. Werler MM, Parker SE, Hedman K, Gissler M, Ritvanen A, Surcel HM. Maternal Antibodies to Herpes Virus Antigens and Risk of Gastroschisis in Offspring. *Am J Epidemiol* 2016; **184**(12): 902-12.

113. Woestenberg PJ, Tjhie JH, de Melker HE, et al. Herpes simplex virus type 1 and type 2 in the Netherlands: seroprevalence, risk factors and changes during a 12-year period. *BMC Infect Dis* 2016; **16**: 364.

114. Wutzler P, Doerr HW, Farber I, et al. Seroprevalence of herpes simplex virus type 1 and type 2 in selected German populations-relevance for the incidence of genital herpes. *J Med Virol* 2000; **61**(2): 201-7.

115. Chacowry Pala K, Baggio S, Tran NT, Girardin F, Wolff H, Gétaz L. Blood-borne and sexually transmitted infections: a cross-sectional study in a Swiss prison. *BMC Infect Dis* 2018; **18**(1): 539.

116. Christensen PB, Engle RE, Jacobsen SE, Krarup HB, Georgsen J, Purcell RH. High prevalence of hepatitis E antibodies among Danish prisoners and drug users. *J Med Virol* 2002; **66**(1): 49-55.

117. Sarmati L, Babudieri S, Longo B, et al. Human herpesvirus 8 and human herpesvirus 2 infections in prison population. *J Med Virol* 2007; **79**(2): 167-73.

118. Bozicevic I, Rode OD, Lepej SZ, et al. Prevalence of sexually transmitted infections among men who have sex with men in Zagreb, Croatia. *AIDS Behav* 2009; **13**(2): 303-9.

119. Bozicevic I, Lepej SZ, Rode OD, et al. Prevalence of HIV and sexually transmitted infections and patterns of recent HIV testing among men who have sex with men in Zagreb, Croatia. *Sex Transm Infect* 2012; **88**(7): 539-44.

120. Bystricka M, Gasparovicova L, Stanekova D, Mokras M, Solarikova L, Russ G. Prevalence of antibodies to herpes simplex virus 2 among homosexual men either positive or negative for human immunodeficiency viruses in Slovakia. *Acta Virol* 2000; **44**(3): 163-7.

121. Bystricka M, Krikova Z, Krcova M, et al. Sexually transmitted infections among prostitutes in Bratislava, Slovakia. *Acta Virol* 2003; **47**(3): 167-72.

122. Eing BR, Lippelt L, Lorentzen EU, et al. Evaluation of confirmatory strategies for detection of type-specific antibodies against herpes simplex virus type 2. *Journal of Clinical Microbiology* 2002; **40**(2): 407-13.

123. Fox J, Taylor GP, Day S, Parry J, Ward H. How safe is safer sex? High levels of HSV-1 and HSV-2 in female sex workers in London. *Epidemiol Infect* 2006; **134**(5): 1114-9.

124. Hill C, McKinney E, Lowndes CM, et al. Epidemiology of herpes simplex virus types 2 and 1 amongst men who have sex with men attending sexual health clinics in England and Wales: implications for HIV prevention and management. *Euro surveillance : bulletin Europeen sur les maladies transmissibles = European communicable disease bulletin* 2009; **14**(47).

125. Linhart Y, Shohat T, Amitai Z, et al. Sexually transmitted infections among brothel-based sex workers in Tel-Aviv area, Israel: high prevalence of pharyngeal gonorrhoea. *Int J STD AIDS* 2008; **19**(10): 656-9.

126. Papadogeorgaki H, Caroni C, Frangouli E, Flemetakis A, Katsambas A, Hadjivassiliou M. Prevalence of sexually transmitted infections in female sex workers in Athens, Greece - 2005. *European journal of dermatology : EJD* 2006; **16**(6): 662-5.

127. Smit C, Pfrommer C, Mindel A, et al. Rise in seroprevalence of herpes simplex virus type 1 among highly sexual active homosexual men and an increasing association between herpes simplex virus type 2 and HIV over time (1984-2003). *Eur J Epidemiol* 2007; **22**(12): 937-44.

128. Al-Sulaiman AM, Vallely PJ, Klapper PE. Comparative performance of a novel herpes simplex virus type 2-specific enzyme-linked immunosorbent assay using a targeted chain oligopeptide, peptide 55. *Clinical and vaccine immunology : CVI* 2009; **16**(6): 931-4.

129. Bamberger E, Madeb R, Steinberg J, et al. Detection of sexually transmitted pathogens in patients with hematospermia. *Israel Medical Association Journal* 2005; **7**(4): 224-7.

130. Cowan FM, Johnson AM, Ashley R, Corey L, Mindel A. Antibody to herpes simplex virus type 2 as serological marker of sexual lifestyle in populations. *Bmj* 1994; **309**(6965): 1325-9.

131. Cusini M, Cusan M, Parolin C, et al. Seroprevalence of herpes simplex virus type 2 infection among attendees of a sexually transmitted disease clinic in Italy. *Sexually Transmitted Diseases* 2000; **27**(5): 292-5.

132. de Ory F, Guisasola ME, Balfagón P, Sanz JC. Comparison of commercial methods of immunoblot, ELISA, and chemiluminescent immunoassay for detecting type-specific herpes simplex viruses-1 and -2 IgG. *J Clin Lab Anal* 2018; **32**(1).

133. Enbom M, Strand A, Falk KI, Linde A. Detection of Epstein-Barr virus, but not human herpesvirus 8, DNA in cervical secretions from Swedish women by real-time polymerase chain reaction. *Sex Transm Dis* 2001; **28**(5): 300-6.

134. Evans BA, Kell PD, Bond RA, MacRae KD, Slomka MJ, Brown DW. Predictors of seropositivity to herpes simplex virus type 2 in women. *Int J STD AIDS* 2003; **14**(1): 30-6.

135. Feldman PA, Steinberg J, Madeb R, et al. Herpes simplex virus type 2 seropositivity in a sexually transmitted disease clinic in Israel. *The Israel Medical Association journal : IMAJ* 2003; **5**(9): 626-8.

136. Glinsek Biskup U, Ursic T, Petrovec M. Laboratory diagnosis and epidemiology of herpes simplex 1 and 2 genital infections. *Acta dermatovenerologica Alpina, Pannonica, et Adriatica* 2015; **24**(2): 31-5.

137. Groen J, van Dijk G, Niesters HG, van Der Meijden WI, Osterhaus AD. Comparison of two enzyme-linked immunosorbent assays and one rapid immunoblot assay for detection of herpes simplex virus type 2-specific antibodies in serum. *Journal of Clinical Microbiology* 1998; **36**(3): 845-7.

138. Janier M, Agbalika F, de La Salmoniere P, Lassau F, Lagrange P, Morel P. Human herpesvirus 8 seroprevalence in an STD clinic in Paris: a study of 512 patients. *Sex Transm Dis* 2002; **29**(11): 698-702.

139. Janier M, Scieux C, Meouchi R, et al. Virological, serological and epidemiological study of 255 consecutive cases of genital herpes in a sexually transmitted disease clinic of Paris (France): a prospective study. *Int J STD AIDS* 2006; **17**(1): 44-9.

140. Joffe H, Bamberger E, Nurkin S, et al. Sexually transmitted diseases amon patients with human immunodeficiency virus in Northern Israel. *Israel Medical Association Journal* 2006; **8**(5): 333-6.

141. Lowhagen GB, Tunback P, Andersson K, Bergstrom T, Johannisson G. First episodes of genital herpes in a Swedish STD population: a study of epidemiology and transmission by the use of herpes simplex virus (HSV) typing and specific serology. *Sex Transm Infect* 2000; **76**(3): 179-82.

142. Lowhagen GB, Tunback P, Andersson K, Johannisson G. Recurrent genital herpes in a population attending a clinic for sexually transmitted diseases. *Acta Derm Venereol* 2001; **81**(1): 35-7.

143. Lowhagen GB, Berntsson M, Bonde E, Tunback P, Krantz I. Acceptance and outcome of herpes simplex virus type 2 antibody testing in patients attending an STD clinic - Recognized and unrecognized infections. *Acta Dermato-Venereologica* 2005; **85**(3): 248-52.

144. Mele A, Franco E, Caprilli F, et al. Genital herpes infection in outpatients attending a sexually transmitted disease clinic in Italy. *Eur J Epidemiol* 1988; **4**(3): 386-8.

145. Mullan HM, Munday PE. The acceptability of the introduction of a type specific herpes antibody screening test into a genitourinary medicine clinic in the United Kingdom. *Sex Transm Infect* 2003; **79**(2): 129-33.

146. Narouz N, Allan PS, Wade AH, Wagstaffe S. Genital herpes serotesting: a study of the epidemiology and patients' knowledge and attitude among STD clinic attenders in Coventry, UK. *Sex Transm Infect* 2003; **79**(1): 35-41.

147. Ramaswamy M, McDonald C, Sabin C, Tenant-Flowers M, Smith M, Geretti AM. The epidemiology of genital infection with herpes simplex virus types 1 and 2 in genitourinary medicine attendees in inner London. *Sex Transm Infect* 2005; **81**(4): 306-8.

148. Roest RW, van der Meijden WI, van Dijk G, et al. Prevalence and association between herpes simplex virus types 1 and 2-specific antibodies in attendees at a sexually transmitted disease clinic. *Int J Epidemiol* 2001; **30**(3): 580-8.

149. Smith NA, Sabin CA, Gopal R, et al. Serologic evidence of human herpesvirus 8 transmission by homosexual but not heterosexual sex. *J Infect Dis* 1999; **180**(3): 600-6.

150. Svennerholm B, Olofsson S, Jeansson S, Vahlne A, Lycke E. Herpes simplex virus type-selective enzyme-linked immunosorbent assay with Helix pomatia lectin-purified antigens. *Journal of Clinical Microbiology* 1984; **19**(2): 235-9.

151. van de Laar MJ, Termorshuizen F, Slomka MJ, et al. Prevalence and correlates of herpes simplex virus type 2 infection: evaluation of behavioural risk factors. *Int J Epidemiol* 1998; **27**(1): 127-34.

152. van Rooijen MS, Roest W, Hansen G, Kwa D, de Vries HJ. False-negative type-specific glycoprotein G antibody responses in STI clinic patients with recurrent HSV-1 or HSV-2 DNA positive genital herpes, The Netherlands. *Sex Transm Infect* 2016; **92**(4): 257-60.

153. Woolley PD, Chandiok S, Pumphrey J, Sharratt S, Shanley L, Bennett S. Serological prevalence of herpes simplex virus type 2 amongst GUM clinic attenders in a district general hospital setting. *Int J STD AIDS* 2000; **11**(6): 379-82.

154. Allan PS, Das S. Prevalence of HSV-1/HSV-2 antibodies in HIV seropositive patients in Coventry, United Kingdom [6]. *Sexually Transmitted Infections* 2004; **80**(1): 77.

155. Andreoletti L, Piednoir E, Legoff J, et al. High seroprevalence of herpes simplex virus type 2 infection in French human immunodeficiency virus type 1-infected outpatients. *J Clin Microbiol* 2005; **43**(8): 4215-7.

156. Lidon F, Padilla S, Garcia JA, et al. Contribution of human herpesvirus 8 and herpes simplex type 2 to progression of carotid intima-media thickness in people living with HIV. *Open Forum Infectious Diseases* 2019; **6**(2).

157. Pere H, Rascanu A, LeGoff J, et al. Herpes simplex virus type 2 (HSV-2) genital shedding in HSV-2-/HIV-1-co-infected women receiving effective combination antiretroviral therapy. *International Journal of STD and AIDS* 2016; **27**(3): 178-85.

158. Spielmann N, Munstermann D, Hagedorn HJ, et al. Time trends of syphilis and HSV-2 co-infection among men who have sex with men in the German HIV-1 seroconverter cohort from 1996-2007. *Sex Transm Infect* 2010; **86**(5): 331-6.

159. Sprenger K, Evison JM, Zwahlen M, et al. Sexually transmitted infections in HIV-infected people in Switzerland: cross-sectional study. *PeerJ* 2014; **2**: e537.

160. Suligoi B, Dorrucci M, Volpi A, Andreoni M, Zerboni R, Rezza G. Prevalence and determinants of herpes simplex virus type 2 infection in a cohort of HIV-positive individuals in Italy. *Sex Transm Dis* 2002; **29**(11): 665-7.

161. van Benthem BH, Spaargaren J, van Den Hoek JA, Merks J, Coutinho RA, Prins M. Prevalence and risk factors of HSV-1 and HSV-2 antibodies in European HIV infected women. *Sex Transm Infect* 2001; **77**(2): 120-4.

162. Ardizzoni A, Manca L, Capodanno F, et al. Detection of follicular fluid and serum antibodies by protein microarrays in women undergoing in vitro fertilization treatment. *Journal of Reproductive Immunology* 2011; **89**(1): 62-9.

163. Berglov A, Hallager S, Panum I, Weis N. Prevalence of herpes -, measles morbillivirus-, parvovirus B19 - and rubella viruses immunoglobulin G among women with chronic hepatitis B of reproductive age in Denmark: A cross-sectional study. *International Journal of Infectious Diseases* 2020; **101**: 269-75.

164. Buxbaum S, Geers M, Gross G, Schofer H, Rabenau HF, Doerr HW. Epidemiology of herpes simplex virus types 1 and 2 in Germany: what has changed? *Medical microbiology and immunology* 2003; **192**(3): 177-81.

165. de Sanjose S, Munoz N, Bosch FX, et al. Sexually transmitted agents and cervical neoplasia in Colombia and Spain. *Int J Cancer* 1994; **56**(3): 358-63.

166. Gentile I, Zappulo E, Bonavolta R, et al. Prevalence of herpes simplex virus 1 and 2 antibodies in patients with autism spectrum disorders. *In Vivo* 2014; **28**(4): 667-71.

167. Lehtinen M, Dillner J, Knekt P, et al. Serologically diagnosed infection with human papillomavirus type 16 and risk for subsequent development of cervical carcinoma: nested case-control study. *Bmj* 1996; **312**(7030): 537-9.

168. Przybylski M, Majewska A, Dzieciatkowski T, et al. Infections due to alphaherpesviruses in early post-transplant period after allogeneic haematopoietic stem cell transplantation: Results of a 5-year survey. *J Clin Virol* 2017; **87**: 67-72.

169. Reinheimer C, Doerr HW. Prevalence of herpes simplex virus type 2 in different risk groups: thirty years after the onset of HIV. *Intervirology* 2012; **55**(6): 395-400.

170. Aldea C, Alvarez CP, Folgueira L, Delgado R, Otero JR. Rapid detection of herpes simplex virus DNA in genital ulcers by real-time PCR using SYBR green I dye as the detection signal. *J Clin Microbiol* 2002; **40**(3): 1060-2.

171. Bruisten SM, Cairo I, Fennema H, et al. Diagnosing genital ulcer disease in a clinic for sexually transmitted diseases in Amsterdam, The Netherlands. *J Clin Microbiol* 2001; **39**(2): 601-5.

172. Doric A, Hadzisejdic I, Hruskar Z, Grahovac M, Grahovac B. Frequency of HSV type 1 and HSV type 2 genital infection in patients attending a sexually transmitted diseases counseling clinic in Zagreb, Croatia. *Clinical Chemistry and Laboratory Medicine* 2012; **50**(4): A85.

173. Grange PA, Jary A, Isnard C, et al. Use of a Multiplex Pcr Assay to Assess the Presence of TREPONEMA PALLIDUM in Mucocutaneous Ulcerations in Patients with Suspected Syphilis. *Journal of clinical microbiology* 2020.

174. Hope-Rapp E, Anyfantakis V, Fouere S, et al. Etiology of genital ulcer disease. A prospective study of 278 cases seen in an STD clinic in Paris. *Sex Transm Dis* 2010; **37**(3): 153-8.

175. Kortekangas-Savolainen O, Orhanen E, Puodinketo T, Vuorinen T. Epidemiology of genital herpes simplex virus type 1 and 2 infections in southwestern Finland during a 10-year period (2003-2012). *Sex Transm Dis* 2014; **41**(4): 268-71.

176. Pérez-Torralba C, Ruiz-Olivares M, Sanbonmatsu-Gámez S, Expósito-Ruíz M, Navarro-Marí JM, Gutiérrez-Fernández J. [Increased infections by herpes simplex virus type 1 and polymicrobials of the genital tract, in the general population of a Spanish middle city]. *Rev Esp Quimioter* 2021; **34**(4): 320-9.

177. Scieux C, Barnes R, Bianchi A, Casin I, Morel P, Perol Y. Lymphogranuloma venereum: 27 cases in Paris. *J Infect Dis* 1989; **160**(4): 662-8.

178. Slomka MJ, Emery L, Munday PE, Moulsdale M, Brown DW. A comparison of PCR with virus isolation and direct antigen detection for diagnosis and typing of genital herpes. *J Med Virol* 1998; **55**(2): 177-83.

179. Al-Hasani AM, Barton IG, Al-Omer LS. Susceptibility of HSV strains from patients with genital herpes treated with various formulations of acyclovir. *Journal of Antimicrobial Chemotherapy* 1986; **18**(SUPPL. B): 113-9.

180. Barton IG, Kinghorn GR, Najem S, Al-Omar LS, Potter CW. Incidence of herpes simplex virus types 1 and 2 isolated in patients with herpes genitalis in Sheffield. *Br J Vener Dis* 1982; **58**(1): 44-7.

181. Challenor R, Theobald N, Pinsent S, Mullan H. The management of first episode genital herpes in genitourinary medicine clinics: a national audit in 2006. *Int J STD AIDS* 2007; **18**(10): 711-4.

182. Christie SN, McCaughey C, McBride M, Coyle PV. Herpes simplex type 1 and genital herpes in Northern Ireland. *Int J STD AIDS* 1997; **8**(1): 68-9.

183. Coyle PV, O'Neill HJ, Wyatt DE, McCaughey C, Quah S, McBride MO. Emergence of herpes simplex type 1 as the main cause of recurrent genital ulcerative disease in women in Northern Ireland. *J Clin Virol* 2003; **27**(1): 22-9.

184. Dundarov S, Andonov P, Bakalov B. Characterization of herpes simplex virus strains isolated from patients with various diseases. *Archives of virology* 1980; **63**(2): 115-21.

185. Edwards S, White C. Genital herpes simplex virus type 1 in women. *Genitourin Med* 1994; **70**(6): 426.

186. Filen F, Strand A, Allard A, Blomberg J, Herrmann B. Duplex real-time polymerase chain reaction assay for detection and quantification of herpes simplex virus type 1 and herpes simplex virus type 2 in genital and cutaneous lesions. *Sex Transm Dis* 2004; **31**(6): 331-6.

187. Grillner L, Landqvist M. Enzyme-linked immunosorbent assay for detection and typing of herpes simplex virus. *Eur J Clin Microbiol* 1983; **2**(1): 39-42.

188. Harrison BL, Anderson ER, Connolly N, et al. Age- and sex-specific incidence of first episode genital herpes simplex virus (HSV) type 1 and 2 in attendees at sexual health services in the North West Coast area in 2016-18. *International Journal of STD and AIDS* 2020; **31**(SUPPL 12): 113.

189. Kortekangas-Savolainen O, Vuorinen T. Trends in herpes simplex virus type 1 and 2 infections among patients diagnosed with genital herpes in a Finnish sexually transmitted disease clinic, 1994-2002. *Sex Transm Dis* 2007; **34**(1): 37-40.

190. Löwhagen G-B, Tunbäck P, Bergström T. Proportion of herpes simplex virus (HSV) type 1 and type 2 among genital and extragenital HSV isolates. *Acta Dermato-Venereologica* 2002; **82**(2).

191. Macho-Aizpurua M, Imaz-Perez M, Alava-Menica JA, et al. Characteristics of genital herpes in Bilbao (Northern Spain): 12-year retrospective study. *Enfermedades Infecciosas y Microbiologia Clinica* 2020.

192. Magdaleno-Tapial J, Hernandez-Bel P, Valenzuela-Onate C, et al. Genital Infection With Herpes Simplex Virus Type 1 and Type 2 in Valencia, Spain: A Retrospective Observational Study. *Actas Dermosifiliogr* 2020; **111**(1): 53-8.

193. Manavi K, McMillan A, Ogilvie M. Herpes simplex virus type 1 remains the principal cause of initial anogenital herpes in Edinburgh, Scotland. *Sex Transm Dis* 2004; **31**(5): 322-4.

194. Matondo P, Stocker DI, Sivapalan S. Herpes simplex virus infection in women: viral subtypes and epidemiological features in a district hospital. *Genitourin Med* 1996; **72**(1): 71-3.

195. Melaugh L, Perry M. HSV serotypes in first episode genital herpes in North West Northern Ireland. *Sexually Transmitted Infections* 2016; **92**(Supplement 1): A100.

196. Nieuwenhuis RF, van Doornum GJ, Mulder PG, Neumann HA, van der Meijden WI. Importance of herpes simplex virus type-1 (HSV-1) in primary genital herpes. *Acta Derm Venereol* 2006; **86**(2): 129-34.

197. Nilsen A, Myrmel H. Changing trends in genital herpes simplex virus infection in Bergen, Norway. *Acta Obstet Gynecol Scand* 2000; **79**(8): 693-6.

198. Nouchi A, Caby F, Palich R, Monsel G, Caumes AE. Travel-associated STI amongst HIV and non-HIV infected travellers. *J Travel Med* 2019; **26**(8).

199. Ooi LJ, Clarke E, Patel R, Nyein S. Epidemiology of genital herpes simplex virus type 1 in the UK has changed significantly in the last decade. *International Journal of STD and AIDS* 2015; **26**(11 SUPPL. 1): 81-2.

200. Parra-Sanchez M, Marcuello Lopez A, Garcia-Rey S, et al. Performance of the HSV OligoGen kit for the diagnosis of herpes simplex virus type 1 and 2. *Diagnostic microbiology and infectious disease* 2016; **85**(3): 315-7.

201. Peutherer JF, Smith IW, Robertson DH. Genital infection with herpes simplex virus type I. *The Journal of infection* 1982; **4**(1): 33-5.

202. Reina J, Gutierrez O, Ruiz de Gopegui E, Padilla E. [Incidence of genital infections caused by herpes simplex virus type 1 (HSV-1) from 1995 to 2003]. *Enferm Infecc Microbiol Clin* 2005; **23**(8): 482-4.

203. Ross JD, Smith IW, Elton RA. The epidemiology of herpes simplex types 1 and 2 infection of the genital tract in Edinburgh 1978-1991. *Genitourin Med* 1993; **69**(5): 381-3.

204. Samra Z, Scherf E, Dan M. Herpes simplex virus type 1 is the prevailing cause of genital herpes in the Tel Aviv area, Israel. *Sex Transm Dis* 2003; **30**(10): 794-6.

205. Sundaram SS, Alderson S, Patel R. HSV-1 genital infection rare in men over the age of 35 years. *Int J STD AIDS* 2009; **20**(1): 68.

206. Scoular A, Norrie J, Gillespie G, Mir N, Carman WF. Longitudinal study of genital infection by herpes simplex virus type 1 in Western Scotland over 15 years. *Bmj* 2002; **324**(7350): 1366-7.

207. Strutt M, Bailey J, Tenant-Flowers M, Graham D, Zuckerman M. Ethnic variation in type of genital herpes simplex virus infection in a South London genitourinary medicine clinic. *J Med Virol* 2003; **69**(1): 108-10.

208. Tarin-Vicente EJ, Sendagorta Cudos E, Servera Negre G, et al. Sexually Transmitted Infections During the First Wave of the COVID-19 Pandemic in Spain. *Actas dermo-sifiliograficas* 2021.

209. Thompson C. Genital herpes simplex typing in genitourinary medicine: 1995-1999. *Int J STD AIDS* 2000; **11**(8): 501-2.

210. van Oeffelen L, Biekram M, Poeran J, et al. Update on neonatal herpes simplex epidemiology in the Netherlands. *Pediatric Infectious Disease Journal* 2018; **37**(8): 806-13.

211. Wilson P, Cropper L, Sharp I, Fink C. Apparent increase in the prevalence of herpes simplex virus type 1 genital infections among women. *Genitourinary medicine* 1994; **70**(3): 228.

212. Wolontis S, Jeansson S. Correlation of herpes simplex virus types 1 and 2 with clinical features of infection. *J Infect Dis* 1977; **135**(1): 28-33.

213. Woolley PD, Kudesia G. Incidence of herpes simplex virus type-1 and type-2 from patients with primary (first-attack) genital herpes in Sheffield. *Int J STD AIDS* 1990; **1**(3): 184-6.
